# Synthesis and Evaluation of Novel Triterpene Analogues of Ursolic Acid as Potential Antidiabetic Agent

**DOI:** 10.1371/journal.pone.0138767

**Published:** 2015-09-25

**Authors:** Panpan Wu, Jie Zheng, Tianming Huang, Dianmeng Li, Qingqing Hu, Anming Cheng, Zhengyun Jiang, Luoying Jiao, Suqing Zhao, Kun Zhang

**Affiliations:** 1 Department of Pharmaceutical Engineering, Faculty of Chemical Engineering and Light Industry, Guangdong University of Technology, Guangzhou, China; 2 Guangxi Institute of Botany, Guangxi Zhuang Autonomous Region and Chinese Academy of Sciences, Guilin, China; University of Akron, UNITED STATES

## Abstract

Ursolic acid (**UA**) is a naturally bioactive compound that possesses potential anti-diabetic activity. The relatively safe and effective molecule intrigued us to further explore and to improve its anti-diabetic activity. In the present study, a series of novel **UA** analogues was synthesized and their structures were characterized. Their bioactivities against the α-glucosidase from baker's yeast were determined *in vitro*. The results suggested that most of the analogues exhibited significant inhibitory activity, especially analogues **8b** and **9b** with the IC_50_ values of 1.27 ± 0.27 μM (**8b**) and 1.28 ± 0.27 μM (**9b**), which were lower than the other analogues and the positive control. The molecular docking and 2D-QSAR studies were carried out to prove that the C-3 hydroxyl could interact with the hydrophobic region of the active pocket and form hydrogen bonds to increase the binding affinity of ligand and the homology modelling protein. Thus, these results will be helpful for understanding the relationship between binding mode and bioactivity and for designing better inhibitors from **UA** analogues.

## Introduction

November 14th in every year, launched by the World Health Organization (WHO) and the International Diabetes Federation (IDF) in 1991, is the United Nations Diabetes Day, which is commonly known as the World Diabetes Day and aims to arouse the global awareness and disillusion of diabetes mellitus (DM) [1]. DM is a complex metabolic disorder characterized by persistent hyperglycaemia, which may cause the patient to acute or chronic complications, including blindness, heart disease, stroke, kidney failure and amputations [2]. WHO projects that DM will be the 7th leading cause of death in 2030 [3]. More than 80% of DM deaths occur in middle- and low-income countries [4]. Type 2 diabetes (T2D) is a chief form of DM which results from the body’s ineffective use of insulin, it comprises 90% of people with diabetes around the world [5,6]. There are an enormous number of therapies available for the treatment and the prevention of DM and its complications, such as insulin therapy, α-glucosidase inhibition, protein tyrosine phosphatase 1B inhibition, along with metabolism adjustment [7–10].

Ursolic acid (**UA**, 3β-hydroxy-urs-12-en-28-oic acid, **1**) is a well-known natural pentacyclic triterpenoid carboxylic acid which is ubiquitous in some traditional medicinal herbs. **UA** and its analogues exhibit a wide range of biological activities, including antibacterial [11], antitumor [12,13], antiviral [14], anti-HIV [15], antioxidative [16] and antimalarial activities [17]. Among them, the anti-diabetic activity is one of the most prominent in both *in vitro* and *in vivo* according to our previous studies [18–20]. In recent years, more and more studies indicates that **UA** and its analogues are potential therapeutic agents for the treatment of DM and its complications [21–24]. In order to find new potential **UA** analogues with higher activities, considerable attempts on structural modification of **UA** have been made, especially at the 3-OH and/or 17-COOH positions [25,26]. However, few studies of **UA** analogues focus on the anti-diabetic.

According to our previous work, a series of halogen-containing **UA** analogues has been synthesized [18,20]. However, their efficacy on α-glucosidase inhibition was decreased while compared with the parent compound **UA**. Therefore, a series of new hydrolyzation analogues has been synthesized in our study. In an attempt to explore the activity and mechanisms of these new analogues, and to study their structure-activity relationships, the bioactivities of these new analogues against α-glucosidase were evaluated *in vitro*. Furthermore, molecular docking studies were also carried out with binding of **UA** analogues in the active site of α-glucosidase, to demonstrate that the hydrophilic moieties can interact with the hydrophobic group of the catalytic pocket and form hydrogen bonds. In addition, this study was also supported by 2D-QSAR model which was set up by partial least squares modelling with R software, in order to explore the structural requirements controlling the observed activities. This is the first study focusing on the anti-diabetic properties of these new hydrolysed analogues.

## Results and Discussion

### Chemistry

**Fig 1:**
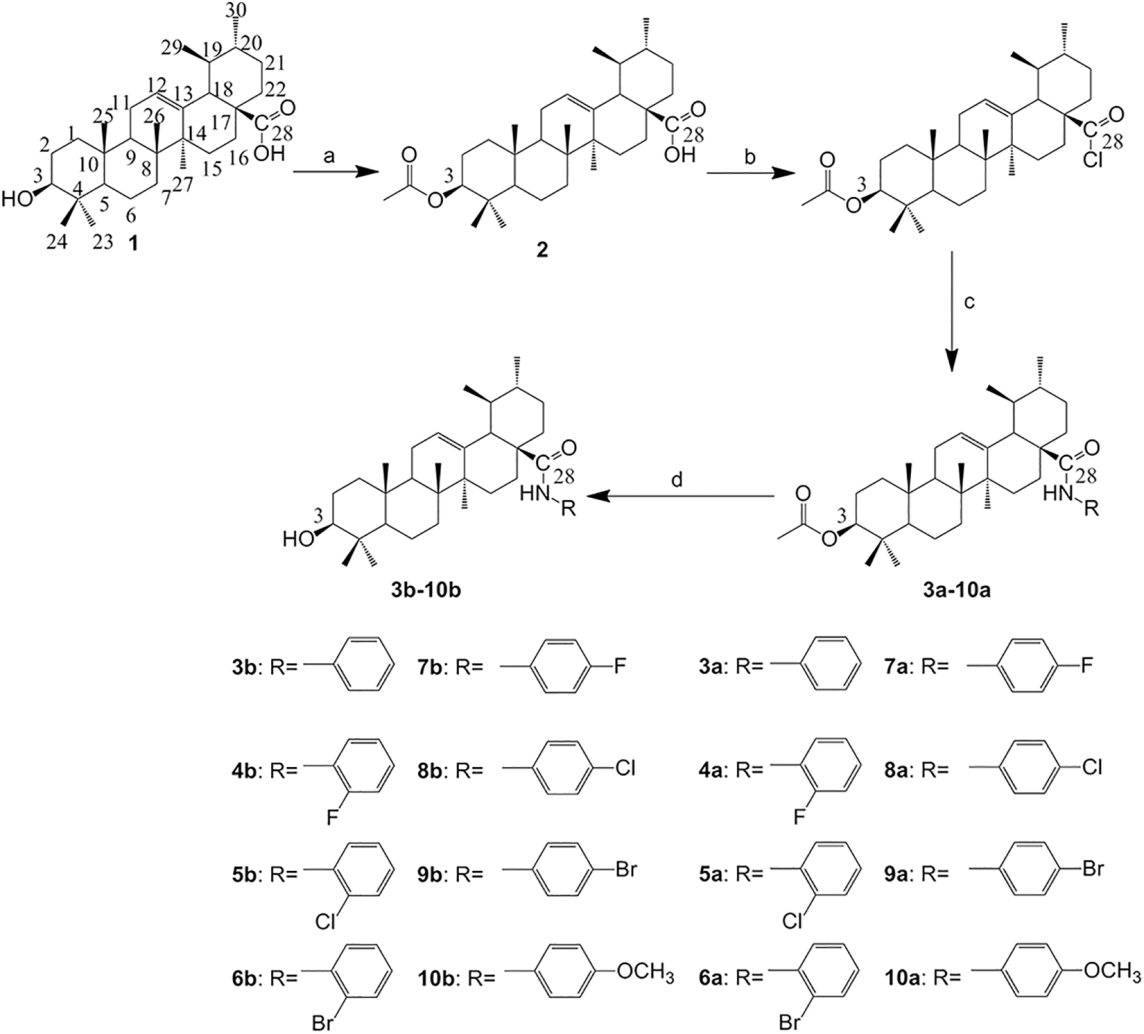
Synthesis of analogues 10a, 3b-10b from UA. Reagents and conditions: (a) acetic anhydride/Pyr/DMAP, r.t.; (b) (COCl)_2_, CH_2_Cl_2_, r.t.; (c) CH_2_Cl_2_, Et_3_N, R-NH_2_, r.t.; (d) NaOH, THF/CH_3_OH, r.t.

**Fig 2:**
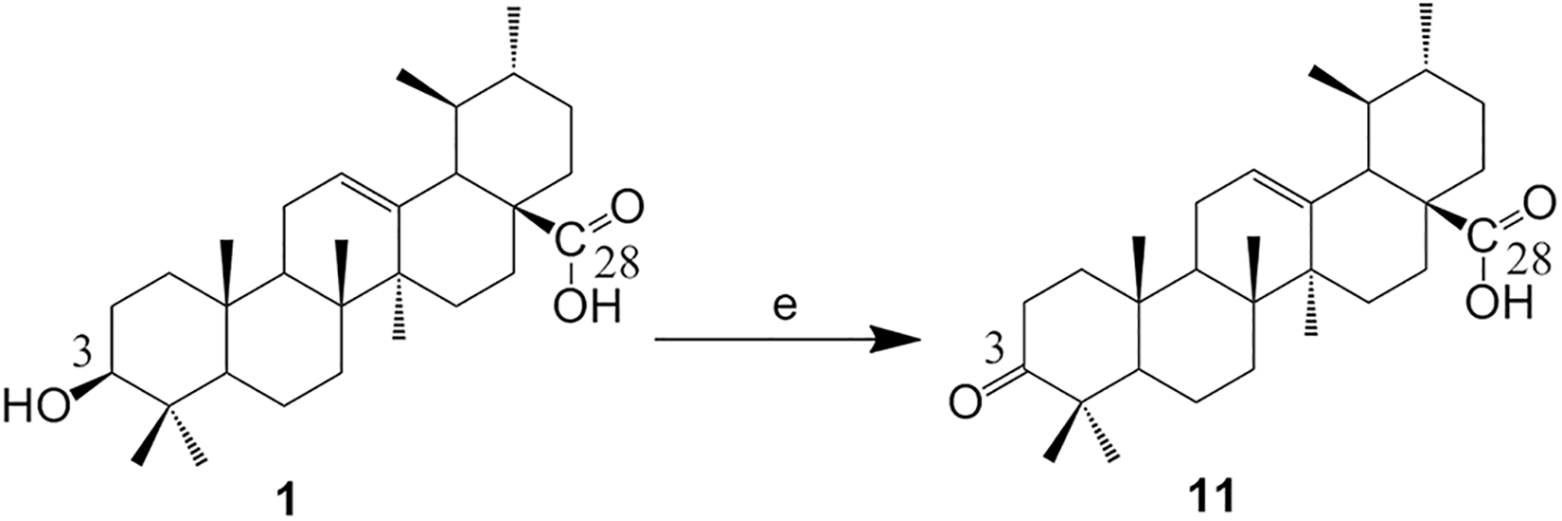
Synthesis of analogue 11 from UA. Reagent and condition: (e) PCC, r.t.

Based on the previous study, with a slight modification using **UA** as the lead compound, structural modifications were made at the 3-OH and/or 17-COOH positions to get a series of new **UA** analogues. The synthetic routes are presented in Fig 1 and Fig 2.

**UA** (**1**) was first esterified with acetic anhydride in anhydrous pyridine to produce its 3-*O*-acetate (**2**), which was then treated with oxalyl chloride to give the intermediate 28-acyl chloride [18]. This intermediate was dissolved in dichloromethane and then condensed with the appropriate amino compounds (aminobenzene, *o*-fluoroaniline, *o*-chloroaniline, *o*-bromobenzenamine, *p*-fluoroaniline, *p*-chloroaniline, *p*-bromobenzenamine, *p*-methoxylaniline) in the presence of triethylamine to produce analogues **3a-10a**, then the saponification analogues of **3a-10a** were hydrolyzed to give the corresponding analogues **3b-10b** (Fig 1) [27]. **UA** was oxidized with pyridinium chlorochromate (PCC) to give the 3-oxo analogue (**11**) [28]. All the target analogues were purified by column chromatography with petroleum ether/ethyl acetate and/or chloroform/methanol as the eluent. Their structures were confirmed by the application of ^1^H NMR (**S1 File**), ^13^C NMR (**S1 File**), mp, electrospray ionization mass spectrometry (ESI-MS) (**S2 File**), high resolution mass spectrometry (HRMS) (**S2 File**).

### *In vitro* α-glucosidase inhibition assay of the UA analogues

In this experiment, α-glucosidase from baker’s yeast was the model which has been widely chosen to determine the anti-diabetic activity of all tested analogues with a slight modification [29,30]. Acarbose was chosen as the positive control, it act by competitively inhibiting the α-glucosidase, a group of key intestinal enzymes involved in the digestion of carbohydrates. A stock solution of each sample, which has been dissolved in dimethylsulfoxide (DMSO) at the concentrations of 0.05 μM to 500 μM, was diluted with 0.1 M phosphate buffer solution (pH = 6.8) containing an appropriate concentration of enzyme solution (0.1 U/mL). After a 10 min pre-incubation at 37°C of the reactions, the substrate (1mM *p*-nitrophenyl-α-D-glucopyranoside) was added to initiate them. Then the reactions were incubated for 30 min at 37°C before they were terminated by adding 1 M Na_2_CO_3_, and their optical density values were measured at 405 nm by using a Multimodel Plate Reader (Infinite 200). Each experiment was repeated at least four times.

**Fig 3:**
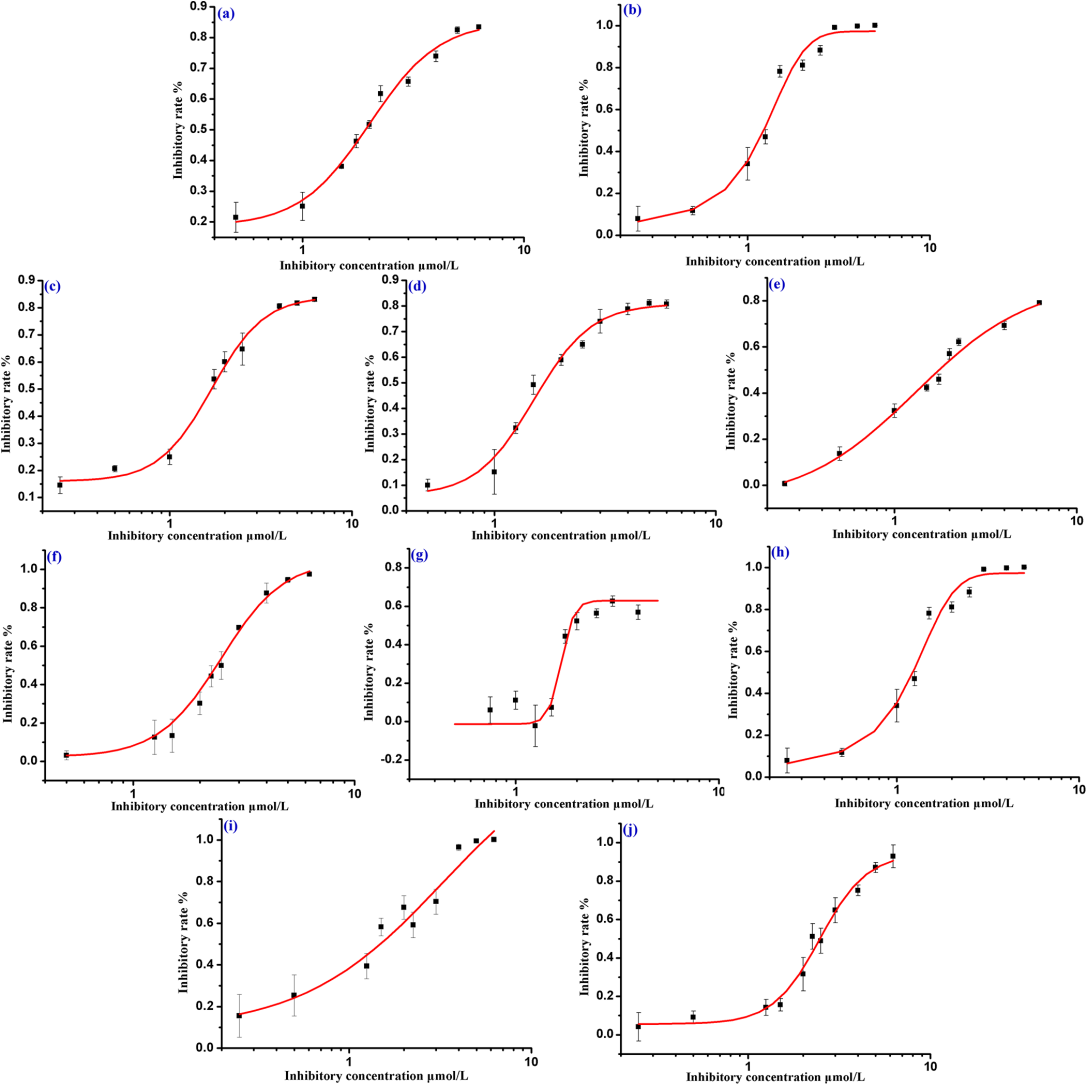
Inhibitory activities of UA analogues against α-glucosidase from baker’s yeast. (a) **10a**, (b-i) **3b-10b**, (j) **11**. The data reported represent the mean (n = 4) ± SD.

To determine the inhibition activity of each sample, the enzyme activity was measured at a fixed substrate concentration, in which a sequence of sample concentrations were tested. The IC_50_ value was calculated according to the curve fit to the sequence of concentrations versus the corresponding inhibition abilities. The results were illustrated in Table 1 and Fig 3. The IC_50_ values of tested samples against α-glucosidase from baker’s yeast ranged from 1.27 μM to 2.56 μM, and it could be concluded that all of them had lower IC_50_ than both positive control and **UA**, indicating that all the synthesized **UA** analogues had significant effect on α-glucosidase inhibition.

**Table 1:** Observed and predicted inhibitory effects of UA analogues against α-glucosidase from baker’s yeast.

Analogue	Polar moiety	IC_50_ ^*a*^ (μM)	Observed pIC_50_	Predicted pIC_50_	Binding free energy (kcal/mol)
**1**	1×OH, 1×COOH	5.08±0.70	5.294	5.282	-3.694
**3b**	1×OH	1.69±0.08	5.772	5.765	-4.092
**4b**	1×OH	1.51±0.10	5.821	5.821	-3.309
**5b**	1×OH	1.31±0.19	5.883	5.875	-3.157
**6b**	1×OH	2.56±0.06	5.592	5.591	-2.879
**7b**	1×OH	1.67±0.09	5.777	5.782	-3.375
**8b**	1×OH	1.27±0.27	5.896	5.903	-3.891
**9b**	1×OH	1.28±0.27	5.893	5.909	-3.488
**10a**	—	2.01±0.12	5.697	5.718	-4.482
**10b**	1×OH	2.55±1.32	5.593	5.599	-4.738
**11**	1×COOH	2.47±0.14	5.607	5.597	-4.332
**12** ^*b*^	13×OH	573.50±15.17	—	—	-9.134

Each experiment was performed in quadruplicate. The data presented represent the means (n = 4) ± SD.

^*a*^IC_50_ value representing the concentration that caused a 50% loss of activity.

^*b*^Acarbose, positive control.

### Structure activity relationship

A total of eleven analogues of **UA** (**10a**, **3b-10b** and **11**) were synthesized and their structure activity relationship (SAR) against α-glucosidase was derived. The **UA** (IC_50_ 5.04 ± 0.80 μM) into its ester 3-*O*-acetate (**2**, IC_50_ 5.27 ± 0.35 μM) at the position of C-3 slightly decreased the activity. Similarly, the analogue **2** into unsubstituted aryl amide analogue, **3a** (IC_50_ 5.64 ± 1.12 μM), showed a slight reduction in activity, and its halogen-containing amide analogues from **4a** to **9a** (IC_50_ ≥ 6.53 ± 1.33 μM) drastically reduced the activity except **8a** (IC_50_ 3.24 ± 0.21 μM) [18]. However, the activities of C-3 hydroxyl analogues **3b**-**10b** were more active than those of the corresponding ester analogues. Furthermore, electronegative (-F,-Cl,-Br) substitutions at the para position in the aryl amide analogues were more potential than the ortho position ones. It is worth noting that electronegative (-OCH_3_) substitution at the para position of the aryl amide analogue has a positive effect on α-glucosidase inhibition. As is indicated in Table 1 and Fig 3, among the C-3 hydroxyl analogues, analogues 8**b** and **9b** were better than the others in this enzyme inhibition model, the IC_50_ values of the two analogues were 1.27 ± 0.27 μM (**8b**) and 1.28 ± 0.27 μM (**9b**), which were about quadruple the starting material **UA**. According to the above results, it might be concluded that **8b** and **9b** possessed potential activity against α-glucosidase.

### Molecular docking mode

SYBYL 2.0, a molecular docking software was introduced to predict the enzyme inhibition of these **UA** analogues. It can not only expound how these **UA** analogues conjugate with the enzyme, but also can provide a guidance for the design of α-glucosidase inhibitors in the future. The molecular docking studies was carried out to survey the binging model of **UA** analogues within the binding pocket of α-glucosidase and to understand their structure-activity relationship.

According to the previous study, homology modelled structure of α-glucosidase has been used for molecular modelling study to identify the reasonable binding mode [20,31,32]. The structure of oligo-1,6-glucosidase from *Saccharomyces cerevisiae* (PDB: 1UOK) was selected as the template because the sequence similarity and identity between α-glucosidase and the template were around 62.0% and 38.0%, respectively [33].

**Fig 4:**
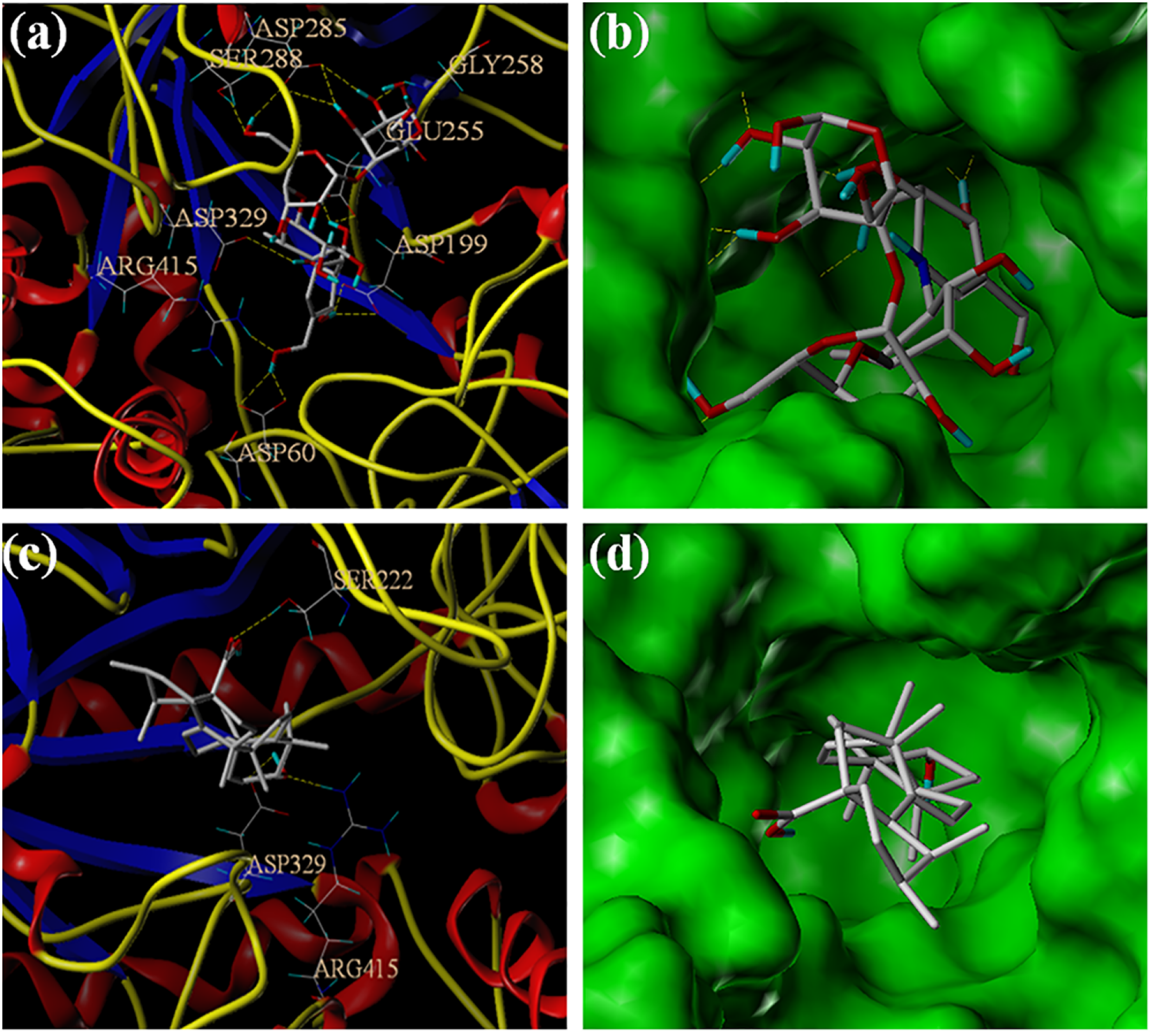
(a) The binding mode of acarbose docked with α-glucosidase. (b) Acarbose with the active site MOLCAD surface representation. (c) The binding mode of UA docked with α-glucosidase. (d) UA with the active site MOLCAD surface representation.

As is indicated in Fig 4, the positive control, acarbose showed higher binding affinity with the homology protein than the parent compound **UA**, and the binding free energy of the both analogues were -9.134 kcal/mol and -3.694 kcal/mol, respectively. From Fig 4A and 4C, acarbose could be formed into hydrogen bonds with ASP60, ASP199, GLU255, GLY258, ASP285, SER288, ASP329 and ARG415 residues in the active site. **UA** which could be interacted with SER222, ASP329 and ARG415 residues possessed lower binding affinity while compared with the positive control. It could be concluded that this binding mode might owning to the large number of hydroxyl groups and the hydrophobic interaction. Above all, as is depicted in Fig 4B and 4D, the analysis of interaction between **UA** and the catalytic pocket is similar with that of acarbose.

**Fig 5:**
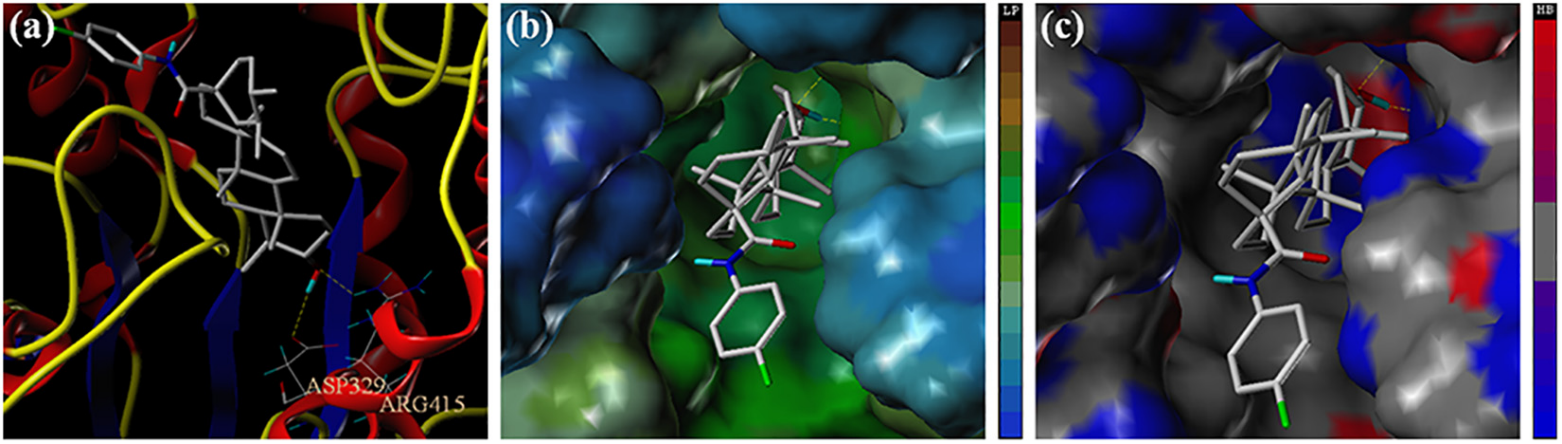
(a) The binding mode of analogue 8b docked with α-glucosidase. (b) Active site MOLCAD surface representation lipophilic potential. (c) Active site MOLCAD surface representation hydrogen bonding.

**Fig 6:**
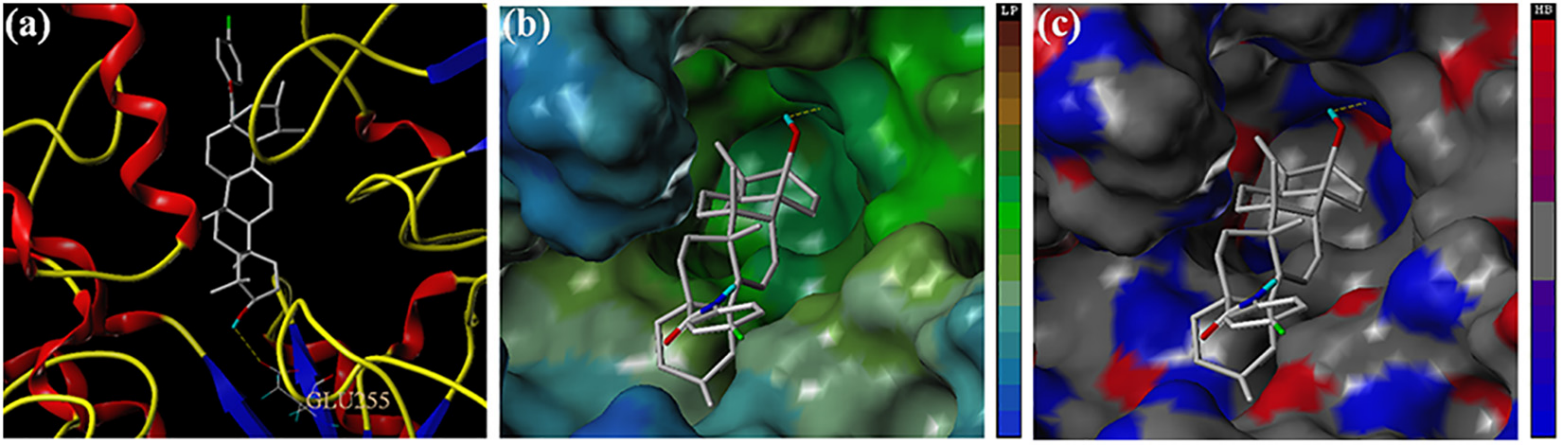
(a) The binding mode of analogue 9b docked with α-glucosidase. (b) Active site MOLCAD surface representation lipophilic potential. (c) Active site MOLCAD surface representation hydrogen bonding.

All of our synthesized **UA** analogues were docked with the developed homology model of α-glucosidase (PDB: 1UOK). The docking studies of two potential analogues (**8b** and **9b**) against α-glucosidase were presented in Figs 5 and 6. The binding free energy of analogues **8b** and **9b** was calculated as -3.891 kcal/mol and -3.488 kcal/mol, which were similar with that of **UA** itself. The two analogues were mainly surrounded by the residues of ASP329, ARG415 and GLU255 in the catalytic pocket. As is shown in Fig 5, analogue **8b** was formed into hydrogen bonds with the residues of ASP329 and ARG415 through the C-3 free hydroxyl group with the inside catalytic pocket. As is depicted in Fig 6, analogue **9b** was formed into hydrogen bonds with the residue of GLU255 through the C-3 free hydroxyl group with the inside catalytic pocket. The MOLCAD lipophilic potential study revealed that the free hydroxyl group at C-3 position of analogues **8b** and **9b** were closed to the hydrophobic region of the active pocket, and it also indicated that more hydrophilic group could improve the inhibitory activity. Besides, the MOLCAD hydrogen bonding study of the binding surface exhibited that several hydrogen bond donors were presented in the hydrophobic pocket while analogues **8b** and **9b** were served as an acceptor by forming two and one hydrogen bonds, respectively. Analogues **8b** and **9b** have significant inhibitory activity through the interaction with the α-glucosidase, which presumably *via* competitively binding active site [20]. Thus, the release of the C-3 free hydroxyl group and the modification on **UA** with more hydrophilic moieties could be of great importance for improving the inhibitory activity of the new **UA** analogues.

**Fig 7:**
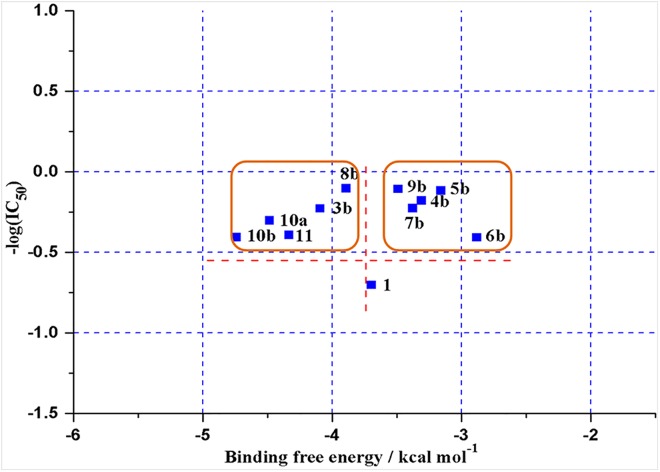
Correlation of binding free energies with inhibitory activities for UA and its analogues.

In order to have a deep insight into the relationship between the *in vitro* inhibitory activity and the docking study, the predicted binding free energies of all tested analogues were calculated by the docking procedures [20,34], presented in Table 1. According to the correlations of predicted binding free energies and the inhibitory activities (see Fig 7), analogues **3b**, **8b**, **10a**, **10b** and **11** exhibited lower predicted binding free energy which were lower than -3.891 kcal/mol, while the other analogues showed a slight higher binding free energy than that of the parent compound **UA**. However, there is no significant difference between the result of predicted binding free energy and *in vitro* enzyme inhibition. It also indicated that choosing the homology protein of α-glucosidase as the docking model could afford some guidance for the selection of α-glucosidase inhibitor.

### 2D-QSAR study

About 30 **UA** analogues (**S3 File**) were studied in the QSAR model, their structures (**S3 File**) and bioactivities could be obtained in our present and previous studies [18,20]. QSAR analyses of these analogues for anti-diabetic activities were performed to correlate the bioactivities with the synthesized analogues, and to identify positive and negative structural features within the four series. The analysis was run by means of Sybyl molecular modelling package, version 2.0 (Tripos, shanghai, China).

**Fig 8:**
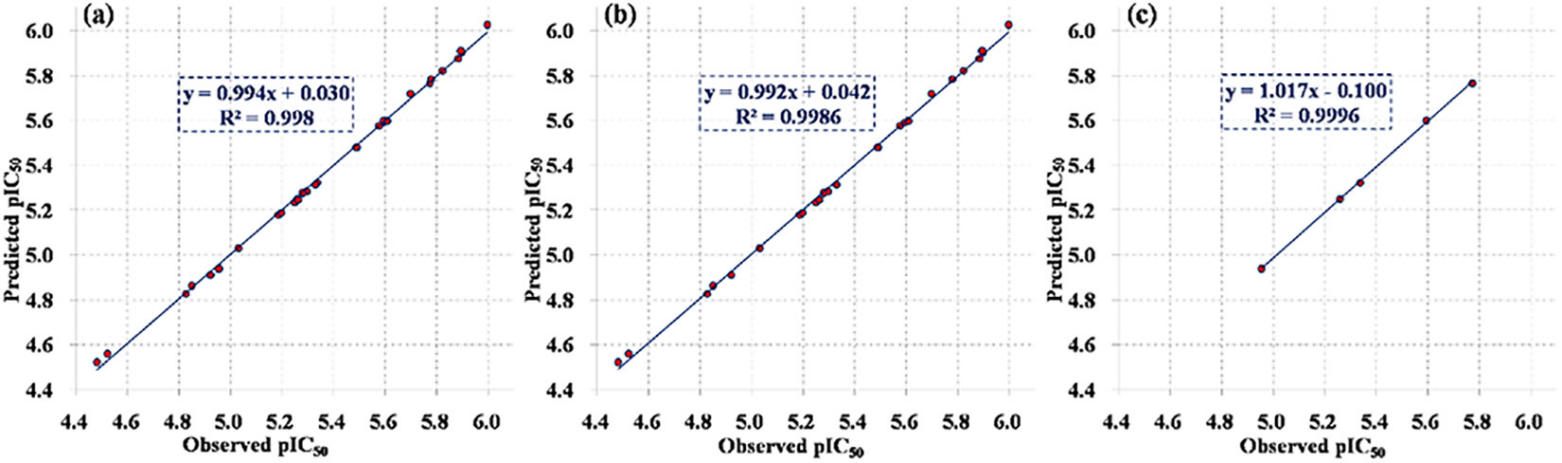
Observed pIC_50_ values versus predicted pIC_50_ values of UA and its analogues against α-glucosidase by 2D-QSAR model. (a) the integration of training and test set, (b) training set, (c) test set.

The 2D-QSAR model was constructed by partial least squares modelling with R software and then evaluated using a training set of 25 analogues and a test set of 5 analogues. The observed pIC_50_ values were validated by measuring the residuals between the observed and the predicted pIC_50_ values of the training set. As shown interestingly in Table 1 and **S4 File**, the predicted pIC_50_ values which were measured by the QSAR model were very close to those observed with very low error (**S4 File**). In addition, from the QSAR model results of predicted pIC_50_ values versus Observed pIC_50_ values (see Fig 8), the root mean square errors (RMSE) of training and test sets were 0.0158 and 0.0113, respectively. The R-squared of this two sets were 0.9986 and 0.9996, respectively. It was indicated that this model could be applied for prediction of more effective hits having the same skeletal framework. Furthermore, from the calculation of the other 2D Descriptors (**S4 File**), each analogue in series b has one more 'hydrogen bond donor' than the others, but one less 'hydrogen bond donor acceptor' than the others (except **UA**), together with their bioactivities, indicating that the C-3 hydroxyl was necessary for their activities against α-glucosidase.

## Conclusions

In summary, we have reported a series of **UA** analogues with a free hydroxyl group at the C-3 position, and their potential *in vitro* inhibitory activity against α-glucosidase has been investigated. All tested **UA** analogues exhibited greater potency than the acarbose and the parent compound **UA** in this α-glucosidase inhibition assay, the bioactivity of C-3 hydroxyl analogues were more potential than their corresponding ester analogues. Among them, electronegative (-F,-Cl,-Br) substitutions at the para position analogues were more active than the ortho ones, especially analogues **8b** and **9b**, whose IC_50_ were 1.27 ± 0.27 μM and 1.28 ± 0.27 μM, respectively. Molecular docking was also studied to identify the binding mode and to afford some guidance for α-glucosidase inhibitor development. The results indicated that the hydrogen bonds formation with the residues of SER222, GLU415, ASP329 and ARG415 between ligands and protein might play an important role in enhancing their inhibitory activity by improving the binding affinity. In addition, validation was employed by measuring the residuals between the observed and the predicted pIC_50_ values by the 2D-QSAR model, indicated that **UA** new analogues which were retained the C-3 hydroxyl and modified with more hydrophilic groups in other positions could be a class of promising analogues as potential α-glucosidase inhibitor.

## Materials and Methods

### General Remarks

**UA** was supplied by Nanjing Zelang Medical Technology Co., Ltd., with a purity of over 98%. Silica gel (100–200 or 200–300 mesh) used in column chromatography was bought from Tsingtao Marine Chemistry Co., Ltd. Other reagents were purchased from commercial suppliers in their chemically or analytically pure grade without further purification unless otherwise noted.

^1^H NMR and ^13^C NMR spectra were recorded on a Bruker AVANCE 400 or Mercury-Plus 300 NMR spectrometers under a standard condition, chemical shifts were measured in ppm downfield from TMS as internal standard (**S1 File**). The melting points were determined on a Fischer-Johns apparatus and are uncorrected. Electrospray ionization (ESI) mass spectra were measured on an LC-MS-2010A and reported as m/z (**S2 File**). High resolution mass spectra of analogues **10a**, **3b-10b**, and **11** were measured on a Bruker maXis impact (**S2 File**). The enzyme inhibition activity was measured using a Multimodel Plate Reader (Infinite 200).

### Synthesis

#### General procedure for the preparation of analogues (2, 3a-10a)

Through the procedure of our previous study, analogue **2** could be obtained after **UA** was treated with acetic anhydride and purified on a silica gel column with petroleum ether/ethyl acetate (v/v 10:1). Then analogue **2** was treated with oxalyl chloride to get the intermediate of 3-*O*-acetylursolyl chloride, analogues **3a-10a** could be obtained after an equal amount of appropriate amino was added and purified on a silica gel column with petroleum ether/ethyl acetate (v/v 10:1) as the eluent. Eight analogues of **UA** were synthesized according to the procedure reported in our earlier publications [18].

N-[3β-acetoxy-urs-12-en-28-oyl]-p-methoxyamiline (**10a**)

Yield 95%; amorphous white powder; ^1^H NMR (300 MHz, DMSO) δ 7.39 (d, *J* = 9.0 Hz, 2H), 6.81 (d, *J* = 9.1 Hz, 2H), 5.26 (s, 1H), 4.38 (dd, *J* = 11.0, 4.7 Hz, 1H), 3.69 (s, 3H), 3.34 (s, 1H), 2.35 (d, *J* = 10.7 Hz, 1H), 1.99 (s, 3H), 1.93–1.73 (m, 4H), 1.73–1.38 (m, 10H), 1.30 (ddd, *J* = 29.2, 12.8, 6.0 Hz, 3H), 1.08 (s, 3H), 1.00 (d, *J* = 1.5 Hz, 2H), 0.94 (d, *J* = 6.0 Hz, 4H), 0.87 (d, *J* = 6.5 Hz, 7H), 0.80 (s, 3H), 0.79 (s, 3H), 0.65 (s, 3H); ^13^C NMR (100 MHz, CDCl_3_) δ 176.1, 171.1, 156.3, 140.3, 131.5, 126.1, 121.5, 114.2, 81.0, 55.6, 55.3, 54.4, 48.5, 47.6, 42.8, 40.0, 39.7, 39.3, 38.5, 37.8, 37.2, 36.9, 32.8, 31.1, 28.5, 28.0, 25.2, 23.68, 23.66, 23.4, 21.4, 21.3, 18.2, 17.4, 17.1, 16.8, 15.7; ESI-MS *m*/*z* 602.2 [M-H]^-^; HRMS (ESI) calculated for C_39_H_58_NO_4_ [M+H]^+^ = 604.4360, found: 604.4383.

#### General procedure for the preparation of analogues (3b-10b)

Analogue **3a** or (**4a-10a**), which was obtained from our laboratory, was dissolved in CH_3_OH/THF (1:1.5, 10 mL) and treated with aqueous NaOH (4N), the reaction was stirred for 4h at room temperature and concentrated in vacuo. The residue was suspended in distilled water and neutralized with 2 N HCl to pH 3, filtered. The filter was washed with distilled water to pH 6, and dried to get an amorphous white powder.

N-[3β-Hydroxy-urs-12-en-28-oyl]-aminobenzene (**3b**)

Yield 98%; amorphous white powder; ^1^H NMR (300 MHz, DMSO) δ 7.51 (d, *J* = 7.5 Hz, 2H), 7.23 (t, *J* = 7.9 Hz, 2H), 6.99 (t, *J* = 7.3 Hz, 1H), 5.27 (d, *J* = 3.4 Hz, 1H), 4.28 (d, *J* = 5.2 Hz, 1H), 3.04–2.92 (m, 1H), 2.37 (d, *J* = 10.8 Hz, 1H), 2.02 (dt, *J* = 14.3, 7.1 Hz, 1H), 1.95–1.64 (m, 5H), 1.60–1.32 (m, 9H), 1.25 (dd, *J* = 21.8, 11.1 Hz, 3H), 1.06 (s, 3H), 0.96 (t, *J* = 12.0 Hz, 5H), 0.87 (d, *J* = 6.5 Hz, 6H), 0.81 (s, 3H), 0.65 (t, *J* = 5.4 Hz, 6H); ^13^C NMR (100 MHz, CDCl_3_) δ 176.4, 140.2, 138.3, 129.0, 126.3, 124.1, 119.8, 79.0, 55.2, 54.4, 48.7, 47.7, 42.8, 40.0, 39.7, 39.2, 38.9, 38.8, 37.2, 37.0, 32.9, 31.0, 28.2, 28.1, 27.3, 25.2, 23.7, 23.4, 21.3, 18.3, 17.4, 17.0, 15.7, 15.6; ESI-MS *m*/*z* 530.3 [M-H]^-^; HRMS (ESI) calculated for C_36_H_54_NO_2_ [M+H]^+^ = 532.4149, found: 532.4163.

N-[3β-Hydroxy-urs-12-en-28-oyl]-o-fluoroaniline (**4b**)

Yield 96%; amorphous white powder; ^1^H NMR (300 MHz, DMSO) δ 7.55 (ddd, *J* = 7.9, 4.2, 2.9 Hz, 1H), 7.25–7.06 (m, 3H), 5.29 (s, 1H), 4.29 (d, *J* = 5.1 Hz, 1H), 3.05–2.94 (m, 1H), 2.30 (d, *J* = 10.9 Hz, 1H), 2.11–1.96 (m, 1H), 1.91–1.67 (m, 5H), 1.65–1.32 (m, 9H), 1.26 (t, *J* = 11.8 Hz, 3H), 1.08 (s, 3H), 1.05–0.91 (m, 5H), 0.88 (d, *J* = 5.7 Hz, 4H), 0.84 (d, *J* = 4.4 Hz, 5H), 0.68 (s, 3H), 0.66 (s, 4H); ^13^C NMR (100 MHz, CDCl_3_) δ 176.8, 153.6, 139.0, 127.2, 126.9, 124.7, 123.8, 121.5, 114.7, 79.1, 55.3, 54.3, 49.4, 47.7, 42.6, 40.0, 39.6, 39.2, 38.9, 38.8, 37.3, 37.0, 32.9, 31.1, 28.3, 28.1, 27.4, 25.3, 23.6, 23.5, 21.3, 18.4, 17.4, 16.6, 15.7, 15.6; ESI-MS *m*/*z*548.2 [M-H]^-^; HRMS (ESI) calculated for C_36_H_53_FNO_2_ [M+H]^+^ = 550.4055, found: 550.4067.

N-[3β-Hydroxy-urs-12-en-28-oyl]-o-chloroaniline (**5b**)

Yield 96%; amorphous white powder; ^1^H NMR (300 MHz, DMSO) δ 7.72 (d, *J* = 8.0 Hz, 1H), 7.45 (d, *J* = 8.0 Hz, 1H), 7.28 (t, *J* = 7.7 Hz, 1H), 7.13 (t, *J* = 7.7 Hz, 1H), 5.31 (s, 1H), 4.27 (d, *J* = 5.1 Hz, 1H), 2.98 (dd, *J* = 14.8, 6.2 Hz, 1H), 2.25 (d, *J* = 10.8 Hz, 1H), 2.15–1.97 (m, 1H), 1.97–1.69 (m, 5H), 1.68–1.33 (m, 9H), 1.33–1.16 (m, 3H), 1.08 (s, 3H), 1.05–0.91 (m, 5H), 0.87 (d, *J* = 8.2 Hz, 5H), 0.82 (s, 4H), 0.67 (s, 3H), 0.66 (s, 4H); ^13^C NMR (100 MHz, CDCl_3_) δ 176.6, 138.3, 135.2, 129.1, 127.8, 127.4, 124.2, 122.8, 121.5, 79.1, 55.3, 54.1, 49.6, 47.7, 42.4, 40.0, 39.7, 39.2, 38.9, 38.8, 37.6, 37.0, 32.8, 31.0, 28.2, 28.0, 27.3, 25.3, 23.8, 23.5, 21.3, 18.4, 17.4, 16.8, 15.7, 15.6; ESI-MS *m*/*z* 564.8 [M-H]^-^; HRMS (ESI) calculated for C_36_H_53_ClNO_2_ [M+H]^+^ = 566.3759, found: 566.3768.

N-[3β-Hydroxy-urs-12-en-28-oyl]-o-bromoaniline (**6b**)

Yield 95%; amorphous white powder; ^1^H NMR (300 MHz, DMSO) δ 7.69 (d, *J* = 8.0 Hz, 1H), 7.61 (d, *J* = 8.0 Hz, 1H), 7.33 (t, *J* = 7.2 Hz, 1H), 7.06 (t, *J* = 7.6 Hz, 1H), 5.32 (s, 1H), 4.28 (d, *J* = 4.7 Hz, 1H), 3.06–2.92 (m, 1H), 2.24 (d, *J* = 10.9 Hz, 1H), 2.17–1.99 (m, 1H), 1.81 (dd, *J* = 24.4, 13.1 Hz, 5H), 1.70–1.34 (m, 9H), 1.24 (dd, *J* = 20.7, 12.6 Hz, 3H), 1.17–1.06 (m, 3H), 0.98 (dd, *J* = 23.8, 8.1 Hz, 5H), 0.88 (d, *J* = 8.3 Hz, 6H), 0.83 (s, 3H), 0.80–0.74 (m, 1H), 0.68 (s, 3H), 0.66 (s, 3H); ^13^C NMR (100 MHz, CDCl_3_) δ 176.4, 138.2, 136.3, 132.4, 128.5, 127.4, 124.8, 122.0, 113.5, 79.1, 55.3, 54.0, 49.6, 47.7, 42.4, 40.0, 39.7, 39.2, 38.9, 38.7, 37.6, 37.1, 32.8, 31.0, 28.2, 28.0, 27.3, 25.3, 23.8, 23.4, 21.3, 18.4, 17.4, 16.9, 15.7, 15.6; ESI-MS *m*/*z* 608.1 [M-H]^-^; HRMS (ESI) calculated for C_36_H_53_BrNO_2_ [M+H]^+^ = 610.3254, found: 610.3262.

N-[3β-Hydroxy-urs-12-en-28-oyl]-p-fluoroaniline (**7b**)

Yield 97%; amorphous white powder; ^1^H NMR (300 MHz, DMSO) δ 7.51 (dd, *J* = 9.0, 5.1 Hz, 2H), 7.08 (t, *J* = 8.9 Hz, 2H), 5.26 (s, 1H), 4.28 (d, *J* = 5.1 Hz, 1H), 3.06–2.91 (m, 1H), 2.35 (d, *J* = 10.7 Hz, 1H), 2.12–1.61 (m, 7H), 1.61–1.32 (m, 9H), 1.32–1.16 (m, 3H), 1.06 (s, 3H), 0.96 (t, *J* = 11.9 Hz, 5H), 0.87 (d, *J* = 7.9 Hz, 6H), 0.81 (s, 3H), 0.65 (t, *J* = 6.0 Hz, 6H); ^13^C NMR (100 MHz, CDCl_3_) δ 176.4, 159.3, 140.3, 134.3, 126.3, 121.5, 121.4, 115.7, 79.1, 55.3, 54.4, 48.7, 47.7, 42.8, 40.0, 39.7, 39.3, 38.9, 38.8, 37.2, 37.0, 32.9, 31.0, 28.3, 28.0, 27.3, 25.3, 23.7, 23.4, 21.3, 18.3, 17.4, 17.0, 15.71, 15.65; ESI-MS *m*/*z* 548.3 [M-H]^-^; HRMS (ESI) calculated for C_36_H_53_FNO_2_ [M+H]^+^ = 550.4055, found: 550.4065.

N-[3β-Hydroxy-urs-12-en-28-oyl]-p-chloroaniline (**8b**)

Yield 97%; amorphous white powder; ^1^H NMR (300 MHz, DMSO) δ 7.56 (d, *J* = 8.9 Hz, 2H), 7.29 (d, *J* = 8.8 Hz, 2H), 5.26 (s, 1H), 4.27 (s, 1H), 3.05–2.91 (m, 1H), 2.36 (d, *J* = 10.7 Hz, 1H), 2.03 (td, *J* = 14.2, 3.8 Hz, 1H), 1.94–1.62 (m, 5H), 1.59–1.32 (m, 9H), 1.24 (dd, *J* = 23.0, 11.4 Hz, 3H), 1.06 (s, 3H), 0.96 (t, *J* = 11.8 Hz, 5H), 0.87 (d, *J* = 7.4 Hz, 6H), 0.81 (s, 3H), 0.66 (d, *J* = 5.9 Hz, 4H), 0.61 (s, 3H); ^13^C NMR (100 MHz, CDCl_3_) δ 176.6, 140.4, 137.4, 132.0, 126.4, 121.2, 116.6, 79.1, 55.3, 54.5, 48.8, 47.7, 42.8, 40.0, 39.7, 39.2, 38.9, 38.8, 37.1, 37.0, 32.8, 31.0, 28.3, 28.0, 27.3, 25.3, 23.7, 23.4, 21.3, 18.3, 17.4, 17.0, 15.71, 15.65; ESI-MS *m*/*z* 564.2 [M-H]^-^; HRMS (ESI) calculated for C_36_H_53_ClNO_2_ [M+H]^+^ = 566.3759, found: 566.3767.

N-[3β-Hydroxy-urs-12-en-28-oyl]-p-bromoaniline (**9b**)

Yield 94%; amorphous white powder; ^1^H NMR(300 MHz, DMSO) δ 7.52 (d, *J* = 8.8 Hz, 2H), 7.42 (d, *J* = 8.8 Hz, 2H), 5.26 (s, 1H), 4.28 (d, *J* = 5.1 Hz, 1H), 2.98 (dd, *J* = 13.1, 7.5 Hz, 1H), 2.35 (d, *J* = 10.5 Hz, 1H), 2.11–1.95 (m, 1H), 1.95–1.61 (m, 5H), 1.61–1.33 (m, 9H), 1.24 (dd, *J* = 23.5, 11.0 Hz, 3H), 1.06 (s, 3H), 0.96 (t, *J* = 11.6 Hz, 5H), 0.87 (d, *J* = 7.6 Hz, 6H), 0.80 (s, 3H), 0.65 (s, 4H), 0.60 (s, 3H); ^13^C NMR (100 MHz, CDCl_3_) δ 176.5, 140.3, 137.4, 132.0, 126.3, 121.2, 116.6, 79.0, 55.2, 54.4, 48.8, 47.6, 42.8, 40.0, 39.7, 39.2, 38.9, 38.8, 37.1, 37.0, 32.8, 31.0, 28.2, 28.0, 27.3, 25.3, 23.7, 23.4, 21.3, 18.3, 17.4, 16.9, 15.7, 15.6. ESI-MS *m*/*z* 610.1 [M-H]^-^; HRMS (ESI) calculated for C_36_H_53_BrNO_2_ [M+H]^+^ = 610.3254, found: 610.3260.

N-[3β-Hydroxy-urs-12-en-28-oyl]-p-methoxylaniline (**10b**)

Yield 96%; amorphous white powder; ^1^H NMR (300 MHz, DMSO) δ 7.38 (d, *J* = 8.9 Hz, 2H), 6.81 (d, *J* = 8.9 Hz, 2H), 5.75 (s, 1H), 5.26 (s, 1H), 4.29 (d, *J* = 5.0 Hz, 1H), 3.69 (s, 3H), 2.99 (dd, *J* = 13.2, 7.8 Hz, 1H), 2.35 (d, *J* = 11.3 Hz, 1H), 2.01 (t, *J* = 13.0 Hz, 1H), 1.89–1.64 (m, 5H), 1.62–1.34 (m, 10H), 1.26 (dd, *J* = 26.7, 13.0 Hz, 3H), 1.06 (s, 3H), 0.94 (d, *J* = 5.7 Hz, 4H), 0.87 (d, *J* = 9.2 Hz, 7H), 0.81 (s, 3H), 0.65 (s, 3H), 0.64 (s, 3H); ^13^C NMR (100 MHz, CDCl_3_) δ 176.1, 156.3, 140.3, 131.5, 126.2, 121.5, 114.2, 79.1, 55.6, 55.3, 54.4, 48.5, 47.7, 42.8, 40.0, 39.7, 39.2, 38.9, 38.8, 37.2, 37.0, 32.9, 31.1, 28.2, 28.1, 27.3, 25.2, 23.7, 23.4, 21.3, 18.3, 17.4, 17.1, 15.7, 15.6; ESI-MS *m*/*z* 560.3 [M-H]^-^; HRMS (ESI) calculated for C_37_H_56_NO_3_ [M+H]^+^ = 562.4255, found: 562.4274.

#### Preparation of 3-Oxo-urs-12-en-28-oic acid (11)

PCC (1.42 g, 6.6 mmol) was added to a solution of **UA** (1.0 g, 2.2 mmol) in acetone-CH_2_Cl_2_ (15 mL), after stirred at room temperature for 8 h, the reaction was concentrated and partitioned with distilled water and CHCl_3_. The organic layer was concentrated and purified by silica gel column chromatography using *n*-hexane-acetone (v/v 95:5) as the eluent to give analogue **11**.

Yield 55%; amorphous white powder; ^1^H NMR (300 MHz, DMSO) δ 5.14 (t, *J* = 3.5 Hz, 1H), 2.36–2.24 (m, 1H), 2.12 (d, *J* = 10.8 Hz, 1H), 2.04–1.70 (m, 4H), 1.68–1.15 (m, 13H), 1.06 (s, 3H), 1.02–0.93 (m, 10H), 0.92 (s, 5H), 0.82 (d, *J* = 8.5 Hz, 7H); ^13^C NMR (100 MHz, CDCl_3_) δ 217.9, 184.0, 138.2, 125.7, 55.4, 52.7, 48.2, 47.5, 46.9, 42.2, 39.6, 39.4, 39.2, 39.0, 36.9, 36.8, 34.3, 32.6, 30.7, 28.1, 26.7, 24.2, 23.7, 23.6, 21.6, 21.3, 19.7, 17.2, 17.1, 15.4; ESI-MS *m*/*z* 453.2 [M-H]^-^.

### α-glucosidase inhibitory activity

The α-glucosidase inhibition assay was performed according to the method of Worawalai et al [30]. with a slight modification. The α-glucosidase enzyme (0.1 U/mL) and substrate (1 mM *p*-nitrophenyl-α-D-glucopyranoside) were dissolved in 0.1 M phosphate buffer, pH 6.8. 10 μL of each synthesized analogue (1 mg/mL in DMSO) was pre-incubated with 8 μL of α-glucosidase at 37°C for 10 min. A 100 μL of substrate solution was then added to the reaction mixture, which was further incubated at 37°C for 30 min. Then, the reaction was terminated by adding 100 μL of 1 M Na_2_CO_3_ solution. Enzymatic activity was quantified by measuring the absorbance at 405 nm with a use of a Multimodel Plate Reader (Infinite 200). The percentage of inhibition was calculated by using [(*A*_*0*_-*A*_*1*_)/*A*_*0*_]×100%, where *A*_*0*_ was the absorbance without the sample, and *A*_*1*_ was the absorbance with the sample. The IC_50_ value was determined from a plot of the percentage of inhibition versus the sample concentration. Acarbose was used as the standard control and the experiment was performed in duplicate.

### Molecular modelling

The molecular minimizing of **UA** analogues were built by use of the Sybyl molecular modelling package, version 2.0 (Tripos, shanghai, China). All structures of test analogues were minimized with the Tripos force field, and the hydrogen atoms were added. Powel optimize the energy gradient, the maximum times to 1000 times the energy convergence criterion reached 0.005 kcal/mol, using Gasteiger–Hückle charges. Ligand-protein docking was performed by the Surflex-Dock in Sybyl 2.0. The crystal structure of α-glucosidase was retrieved from RCSB Protein Data Bank (PDB: 1UOK). Biopolymer module was then used to repair the crystal structure of the protein termini treatment, fix side chain amides, residues and add charges. The potent **UA** analogues docking with the α-glucosidase selected catalytic pocket of acarbose as active site. The active pocket form by computing, the others are the default settings. The binding free energies (*ΔG*_*binding*_) were estimated by an extended model of the unbound states of the receptor and the ligands (1):

$$\Delta G_{binding}=RT\ln K_{d}$$ (1)

## Supporting Information

S1 File Characterization of UA analogues 10a, 3b-10b, and 11 by application of ^1^H NMR and^13^C NMR. (PDF)

S2 File Characterization of UA analogues 10a, 3b-10b, and 11 by application of ESI-MS and HRMS. (PDF)

S3 File The structure of UA and its analogues which were studied in QSAR model. (DOCX)

S4 File Computer descriptors and pIC_50_ of UA analogues. (XLSX)
