# Supplementary material for: Synthesis and Evaluation of Novel Triterpene Analogues of Ursolic Acid as Potential Antidiabetic Agent
Source: PLoS One. 2015 Sep 25;10(9):e0138767. doi: 10.1371/journal.pone.0138767 (PMC4583267; doi:10.1371/journal.pone.0138767)

Synthesis and evaluation of novel triterpene analogues of ursolic acid as potential antidiabetic agent

Pan-Pan Wu, Tian-Ming Huang, Qing-Qing Hu, An-Ming Cheng, Zheng-Yun Jiang, Luo-Ying Jiao, Su-Qing Zhao*, Kun Zhang*

Supplementary Data

Copies of ^{1}H NMR and ^{13}C NMR spectra of compounds **10a**, **3b-10b** and **11**.

Compound **10a:** ***N-[3β-Acetoxy-urs-12-en-28-oyl]-p-methoxyamiline***

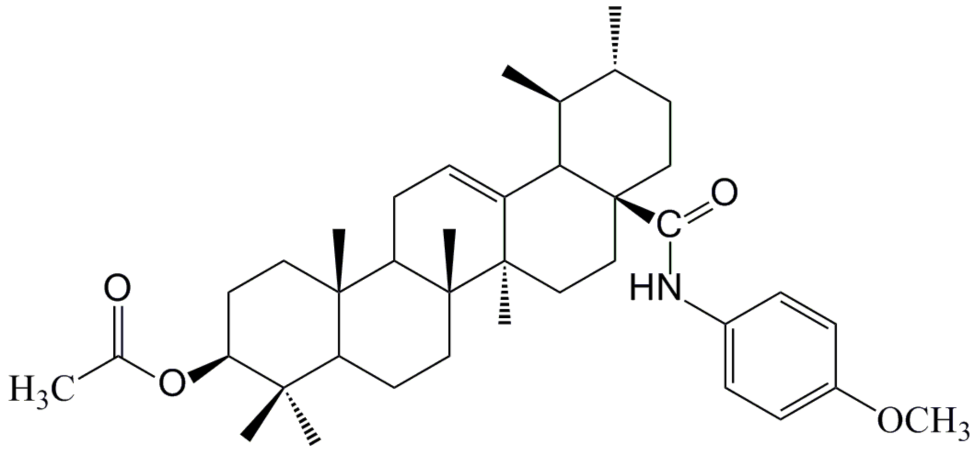

Figure 1. The structure of compound **10a**.

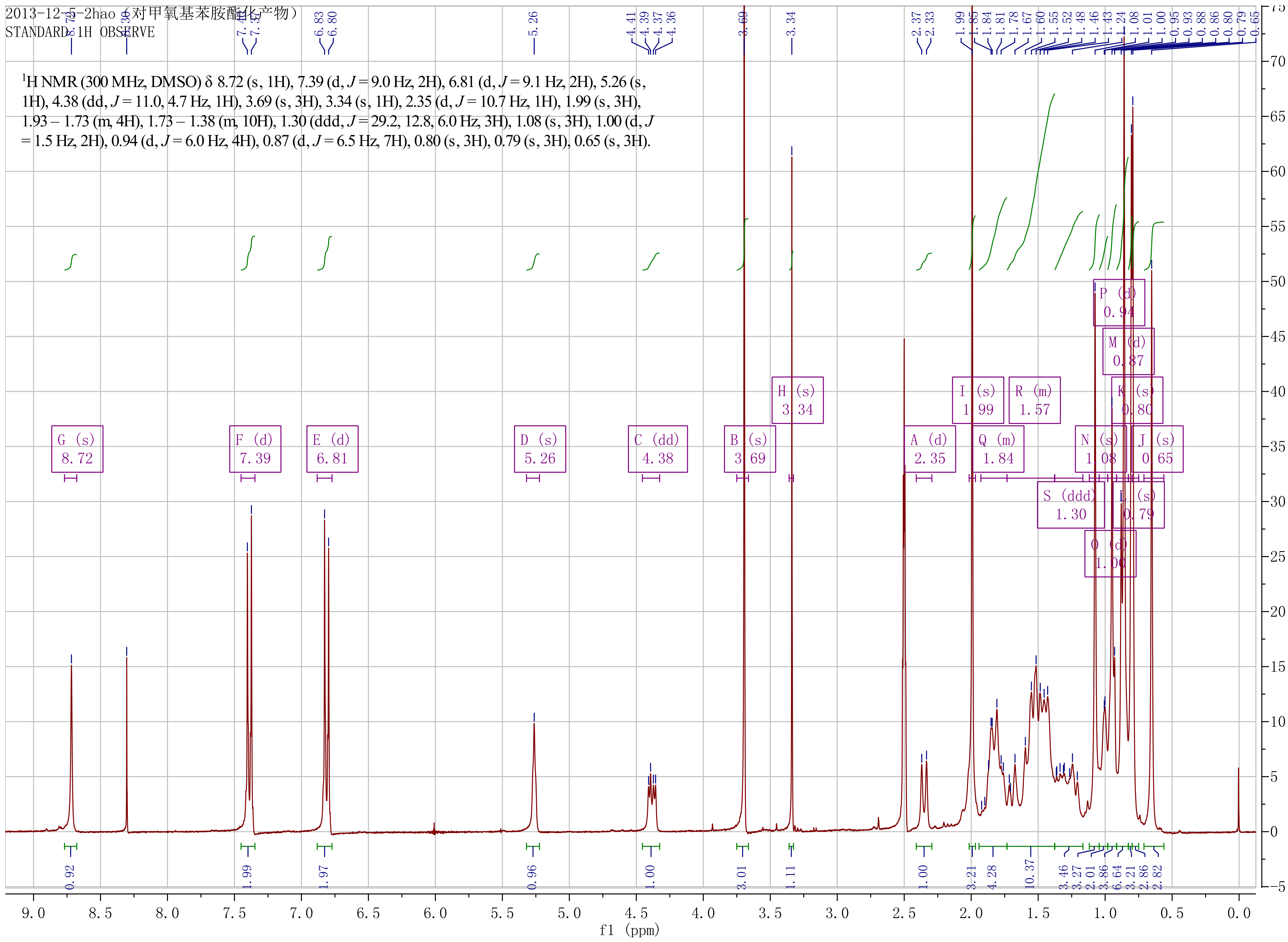
2013-12-5-2hao（对甲氧基苯胺酯化产物）
STANDARD 1H OBSERVE
^{1}H NMR (300 MHz, DMSO) δ 8.72 (s, 1H), 7.39 (d, J = 9.0 Hz, 2H), 6.81 (d, J = 9.1 Hz, 2H), 5.26 (s, 1H), 4.38 (dd, J = 11.0, 4.7 Hz, 1H), 3.69 (s, 3H), 3.34 (s, 1H), 2.35 (d, J = 10.7 Hz, 1H), 1.99 (s, 3H), 1.93 – 1.73 (m, 4H), 1.73 – 1.38 (m, 10H), 1.30 (ddd, J = 29.2, 12.8, 6.0 Hz, 3H), 1.08 (s, 3H), 1.00 (d, J = 1.5 Hz, 2H), 0.94 (d, J = 6.0 Hz, 4H), 0.87 (d, J = 6.5 Hz, 7H), 0.80 (s, 3H), 0.79 (s, 3H), 0.65 (s, 3H).
f1 (ppm)

2015-05-12 WU-FA-02

^{13}C NMR (101 MHz, $CDCl_3$) δ 176.14, 171.13, 156.27, 140.31, 131.52, 126.05, 121.49, 114.18, 80.95, 55.58, 55.34, 54.43, 48.54, 47.61, 42.76, 40.03, 39.71, 39.26, 38.48, 37.79, 37.21, 36.94, 32.82, 31.05, 28.18, 28.04, 25.21, 23.68, 23.66, 23.38, 21.43, 21.33, 18.23, 17.44, 17.08, 16.82, 15.71.

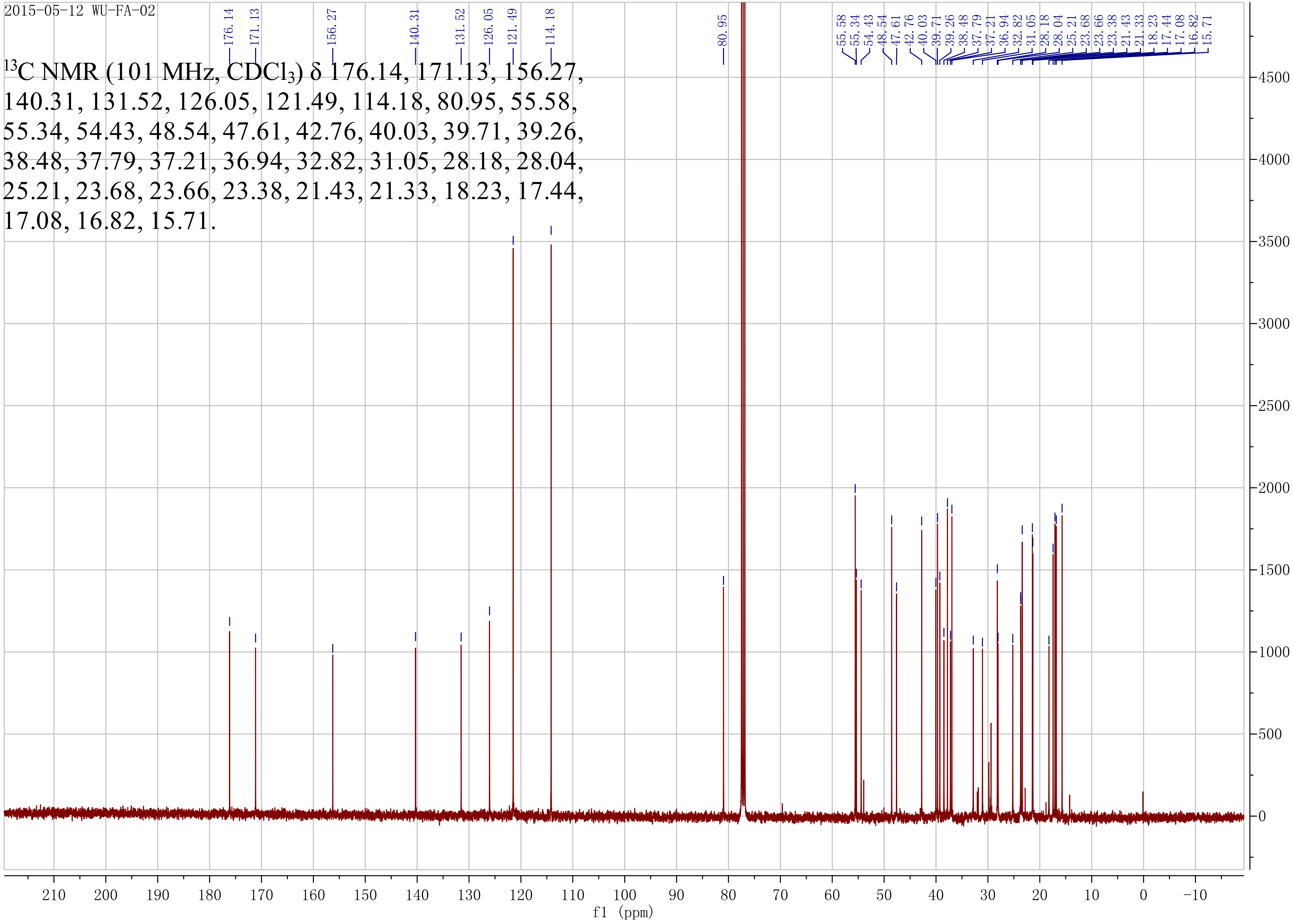

Compound **3b:** ***N-[3β-Hydroxy-urs-12-en-28-oyl]-aminobenzene***

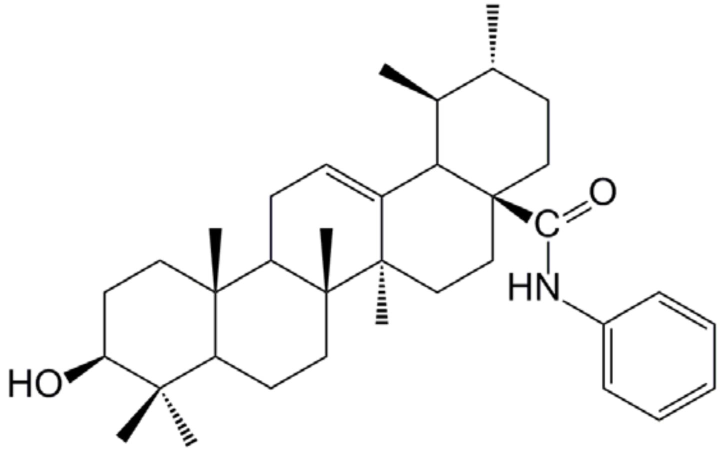

Figure 2. The structure of compound **3b**.

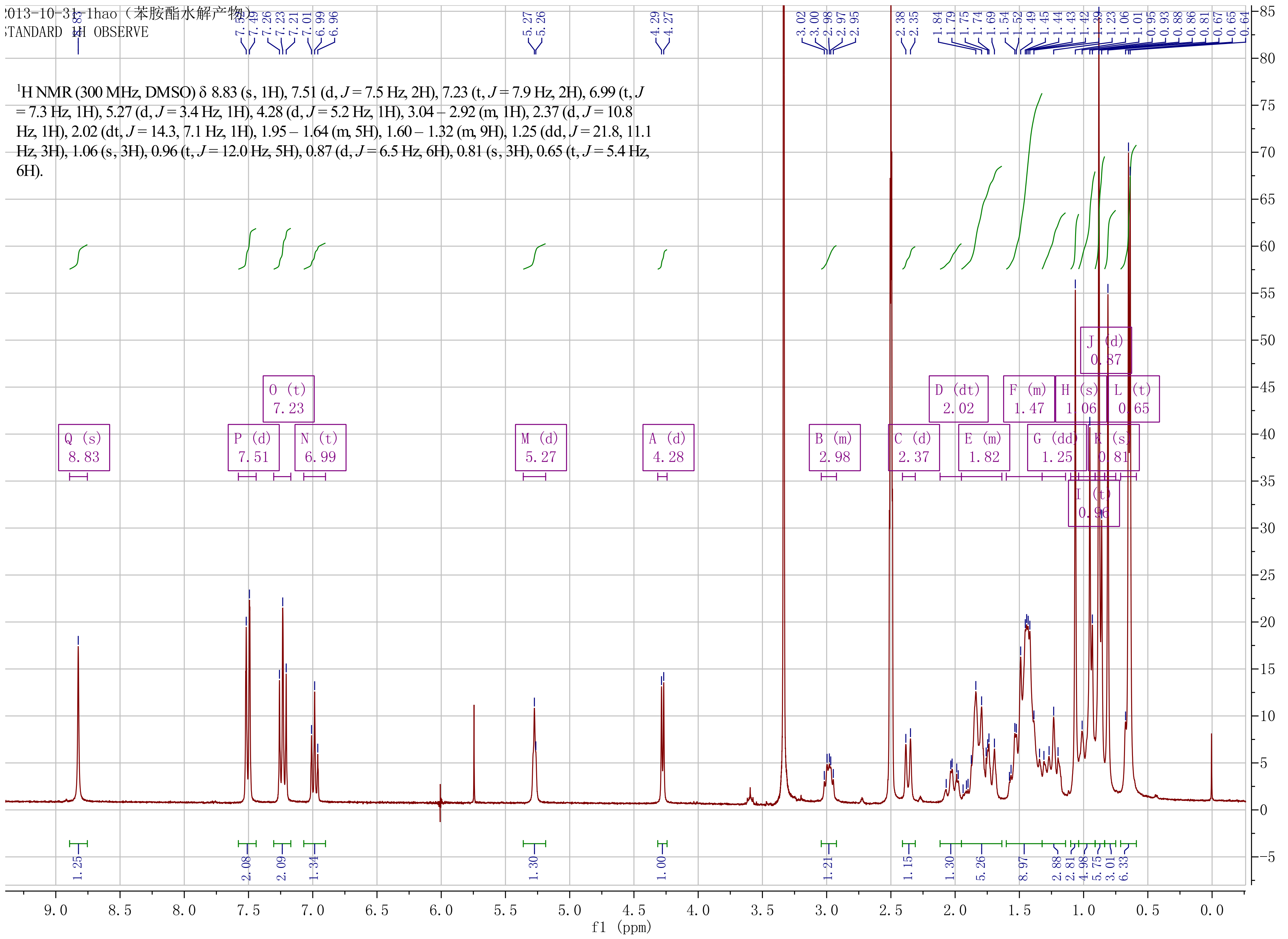
2013-10-31-1hao（苯胺酯水解产物）
STANDARD 1H OBSERVE
^{1}H NMR (300 MHz, DMSO) δ 8.83 (s, 1H), 7.51 (d, J = 7.5 Hz, 2H), 7.23 (t, J = 7.9 Hz, 2H), 6.99 (t, J = 7.3 Hz, 1H), 5.27 (d, J = 3.4 Hz, 1H), 4.28 (d, J = 5.2 Hz, 1H), 3.04 – 2.92 (m, 1H), 2.37 (d, J = 10.8 Hz, 1H), 2.02 (dt, J = 14.3, 7.1 Hz, 1H), 1.95 – 1.64 (m, 5H), 1.60 – 1.32 (m, 9H), 1.25 (dd, J = 21.8, 11.1 Hz, 3H), 1.06 (s, 3H), 0.96 (t, J = 12.0 Hz, 5H), 0.87 (d, J = 6.5 Hz, 6H), 0.81 (s, 3H), 0.65 (t, J = 5.4 Hz, 6H).
f1 (ppm)

2015-05-12 WU-UA-09

^{13}C NMR (101 MHz, $CDCl_3$) δ 176.40, 140.24, 138.28, 129.01, 126.28, 124.08, 119.77, 79.04, 55.24, 54.44, 48.71, 47.66, 42.76, 40.03, 39.66, 39.24, 38.85, 38.81, 37.16, 37.02, 32.87, 31.04, 28.24, 28.05, 27.31, 25.24, 23.69, 23.42, 21.31, 18.32, 17.39, 16.98, 15.70, 15.63.

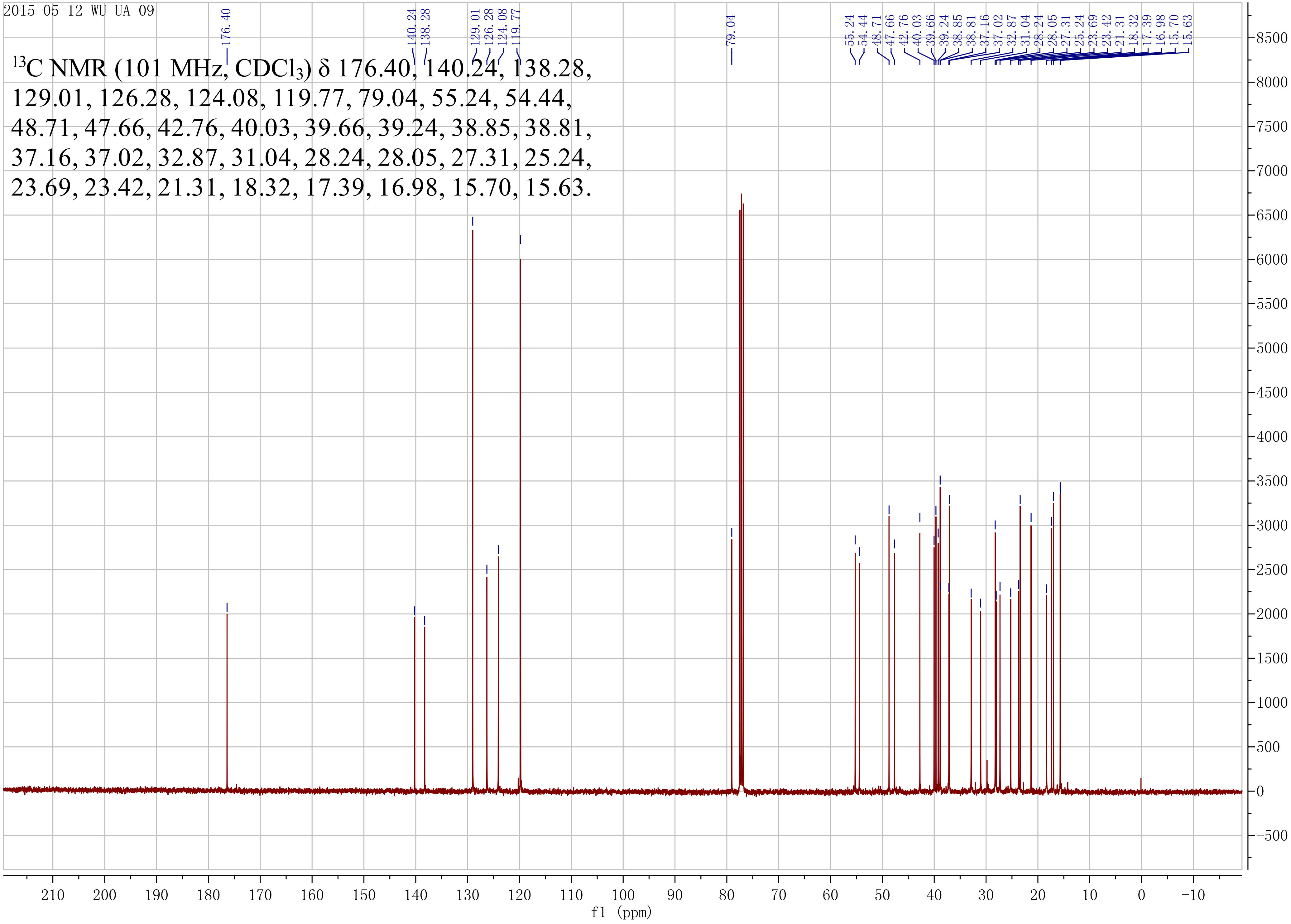

Compound **4b:** ***N-[3β-Hydroxy-urs-12-en-28-oyl]-o-fluoroaniline***

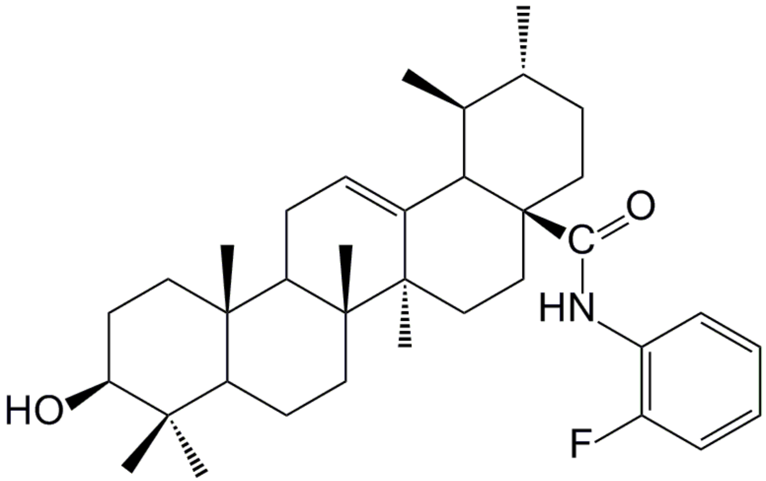

Figure 3. The structure of compound **4b**.

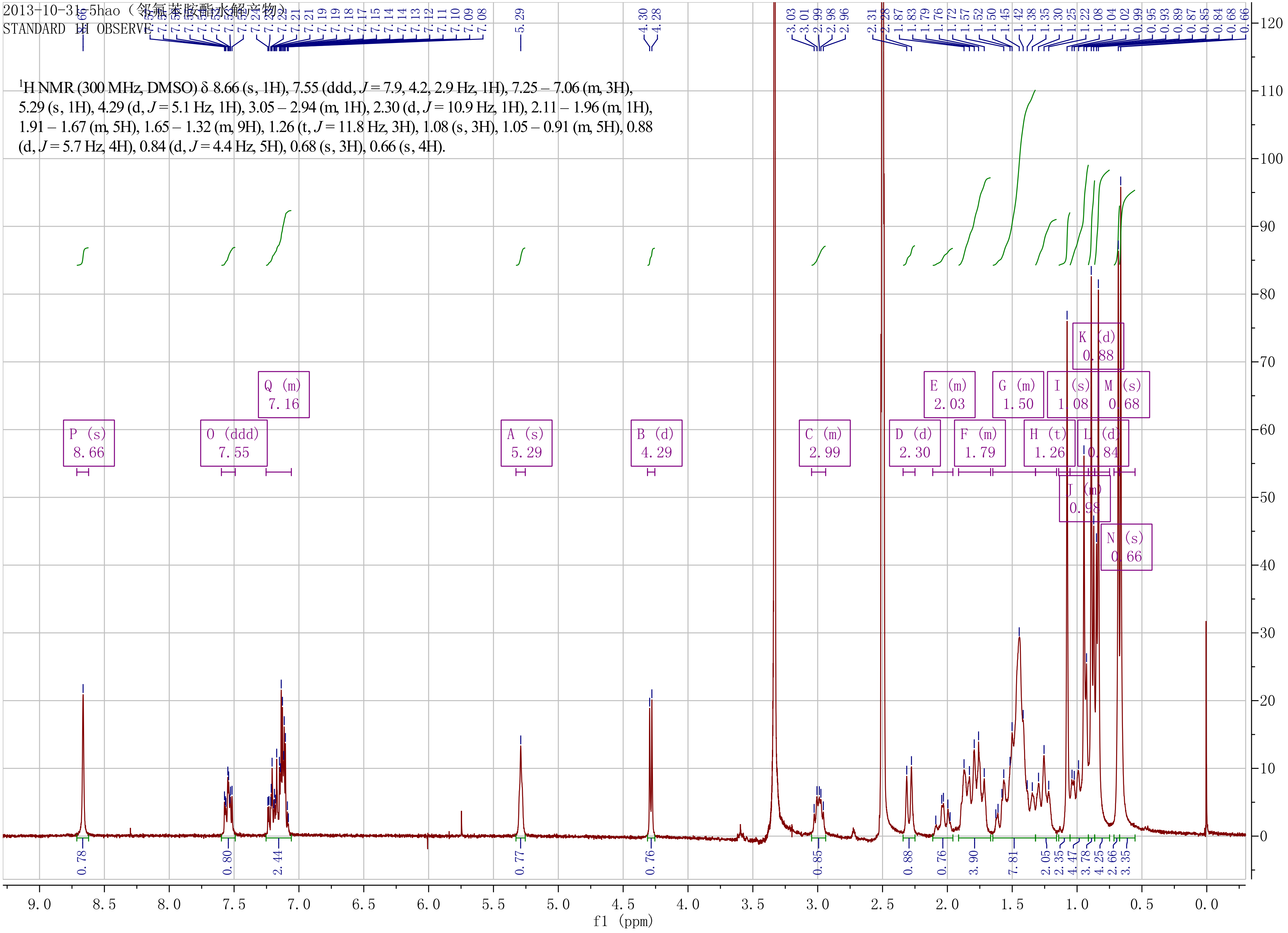
2013-10-31-5hao（邻氟苯胺酯水解产物）
STANDARD 1H OBSERVE
^{1}H NMR (300 MHz, DMSO) δ 8.66 (s, 1H), 7.55 (ddd, J = 7.9, 4.2, 2.9 Hz, 1H), 7.25 – 7.06 (m, 3H), 5.29 (s, 1H), 4.29 (d, J = 5.1 Hz, 1H), 3.05 – 2.94 (m, 1H), 2.30 (d, J = 10.9 Hz, 1H), 2.11 – 1.96 (m, 1H), 1.91 – 1.67 (m, 5H), 1.65 – 1.32 (m, 9H), 1.26 (t, J = 11.8 Hz, 3H), 1.08 (s, 3H), 1.05 – 0.91 (m, 5H), 0.88 (d, J = 5.7 Hz, 4H), 0.84 (d, J = 4.4 Hz, 5H), 0.68 (s, 3H), 0.66 (s, 4H).
f1 (ppm)

^{13}C NMR (101 MHz, $CDCl_3$) δ 176.75, 153.83, 151.42, 139.00, 127.20, 126.98, 126.89, 124.71, 124.68, 123.81, 123.74, 121.51, 121.50, 114.81, 114.62, 79.12, 55.27, 54.31, 49.39, 47.71, 42.61, 40.03, 39.63, 39.22, 38.88, 38.82, 37.28, 37.03, 32.87, 31.07, 28.25, 28.07, 27.35, 25.33, 23.61, 23.52, 21.32, 18.36, 17.35, 16.61, 15.70, 15.63.

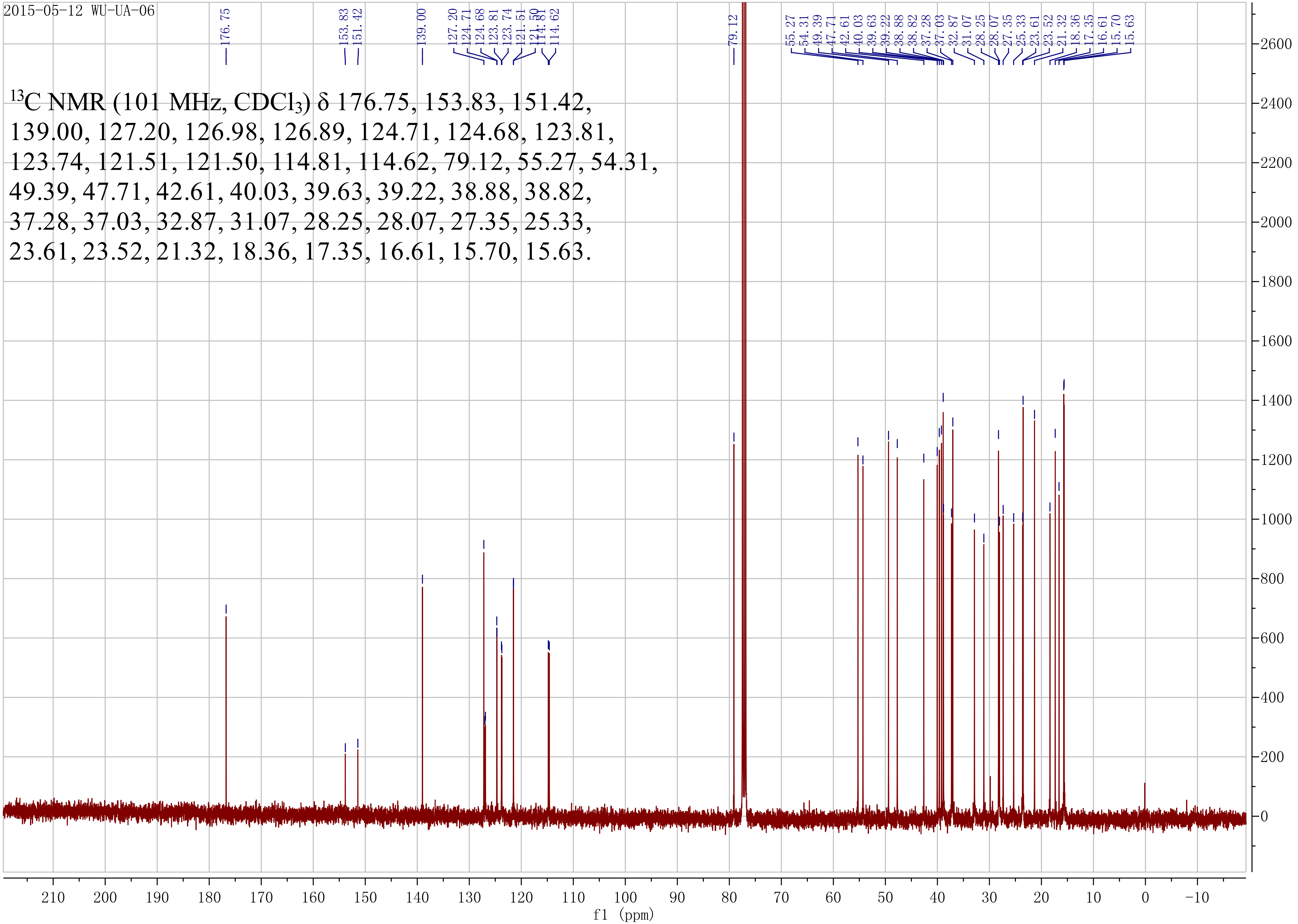

Compound **5b:** ***N-[3β-Hydroxy-urs-12-en-28-oyl]-o-chloroaniline***

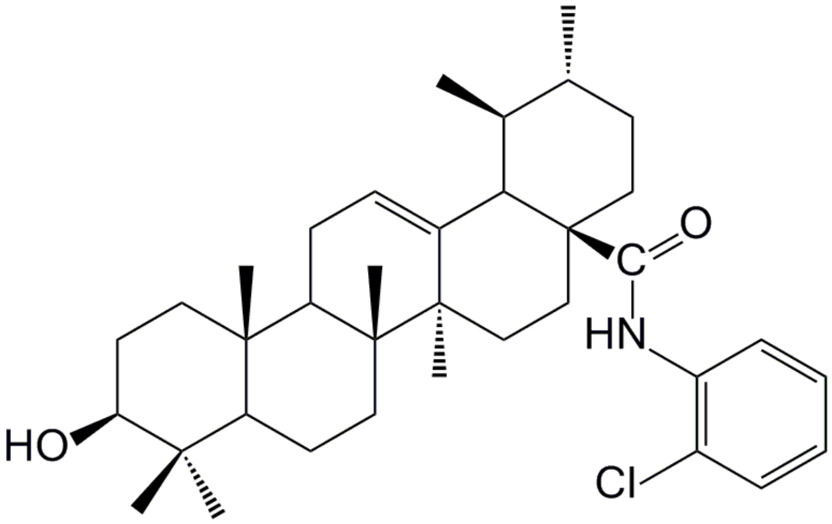

Figure 4. The structure of compound **5b**.

2013-10-31-6hao（邻氯苯胺酯水解产物）
STANDARD 1H OBSERVE

^{1}H NMR (300 MHz, DMSO) δ 8.59 (s, 1H), 7.72 (d, J = 8.0 Hz, 1H), 7.45 (d, J = 8.0 Hz, 1H), 7.28 (t, J = 7.7 Hz, 1H), 7.13 (t, J = 7.7 Hz, 1H), 5.31 (s, 1H), 4.27 (d, J = 5.1 Hz, 1H), 2.98 (dd, J = 14.8, 6.2 Hz, 1H), 2.25 (d, J = 10.8 Hz, 1H), 2.15 – 1.97 (m, 1H), 1.97 – 1.69 (m, 5H), 1.68 – 1.33 (m, 9H), 1.33 – 1.16 (m, 3H), 1.08 (s, 3H), 1.05 – 0.91 (m, 5H), 0.87 (d, J = 8.2 Hz, 5H), 0.82 (s, 4H), 0.67 (s, 3H), 0.66 (s, 4H).

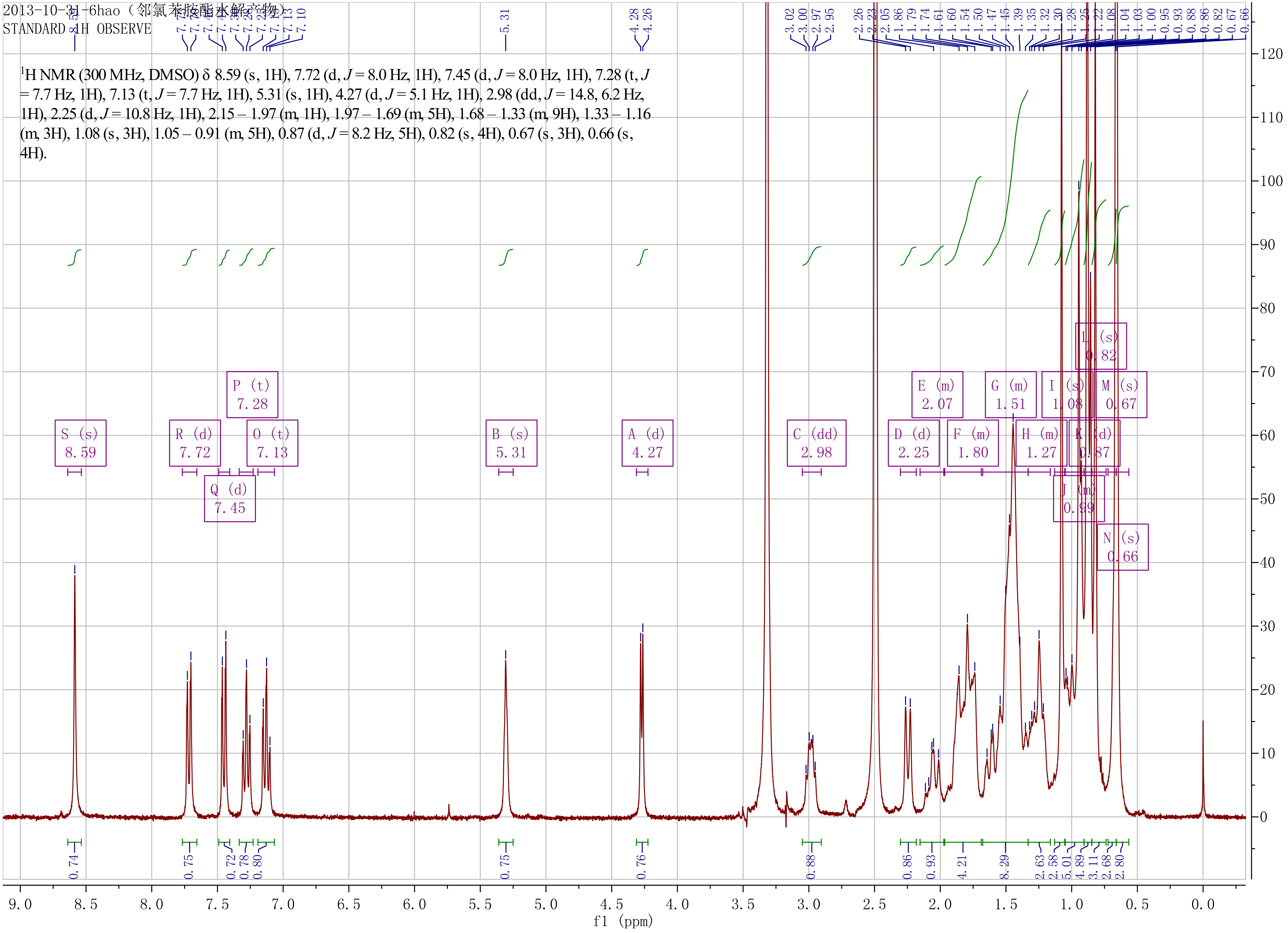

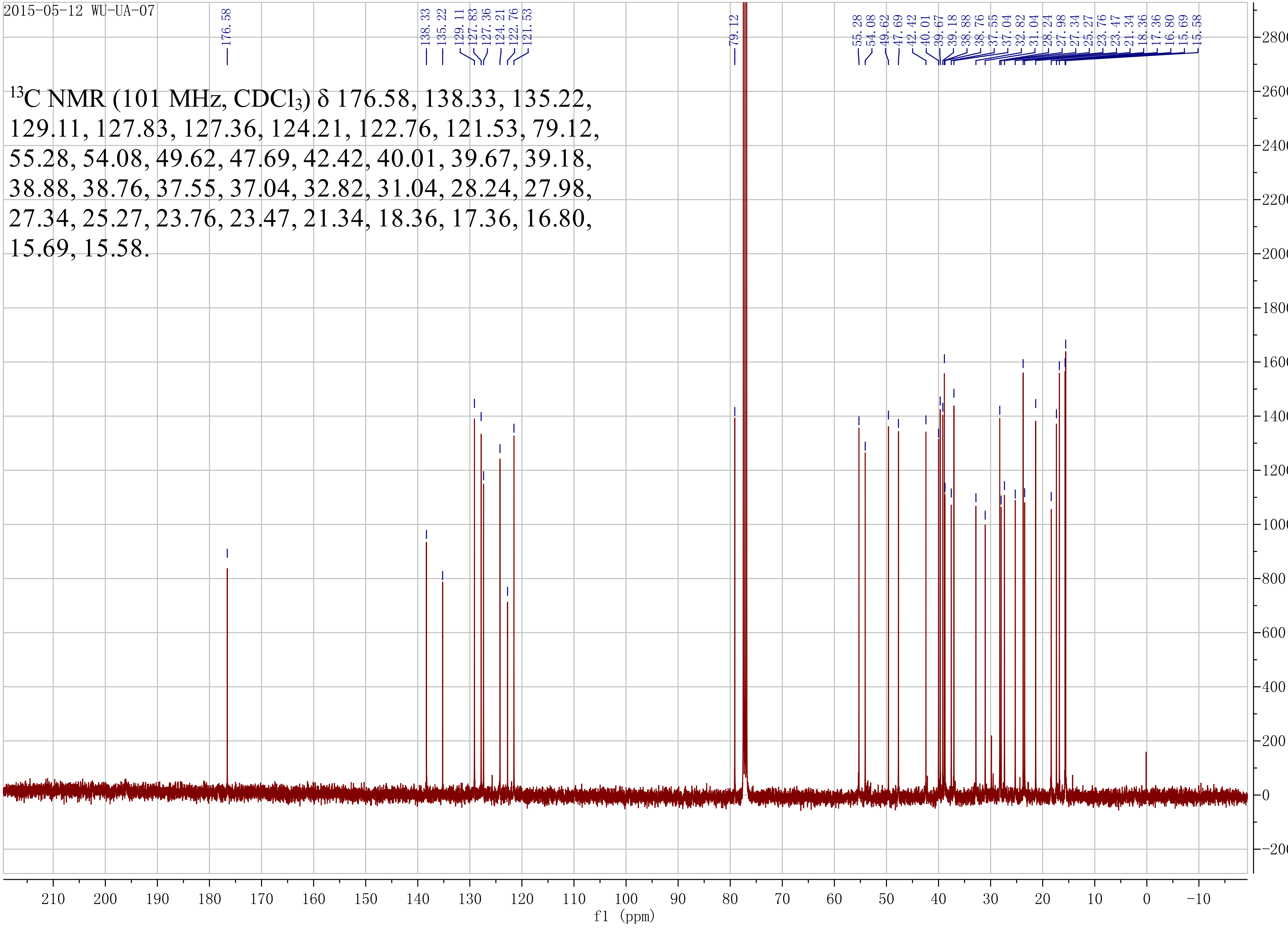

2015-05-12 WU-UA-07
^{13}C NMR (101 MHz, CDCl3) δ 176.58, 138.33, 135.22, 129.11, 127.83, 127.36, 124.21, 122.76, 121.53, 79.12, 55.28, 54.08, 49.62, 47.69, 42.42, 40.01, 39.67, 39.18, 38.88, 38.76, 37.55, 37.04, 32.82, 31.04, 28.24, 27.98, 27.34, 25.27, 23.76, 23.47, 21.34, 18.36, 17.36, 16.80, 15.69, 15.58.
f1 (ppm)

Compound **6b:** ***N-[3β-Hydroxy-urs-12-en-28-oyl]-o-bromoaniline***

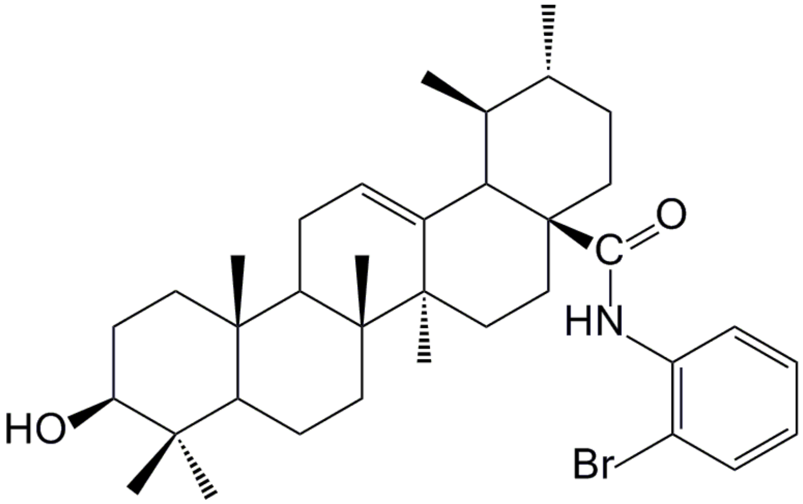

Figure 5. The structure of compound **6b**.

2013-10-31-7hao（邻溴苯胺酯水解产物）

STANDARD 1H OBSERVE

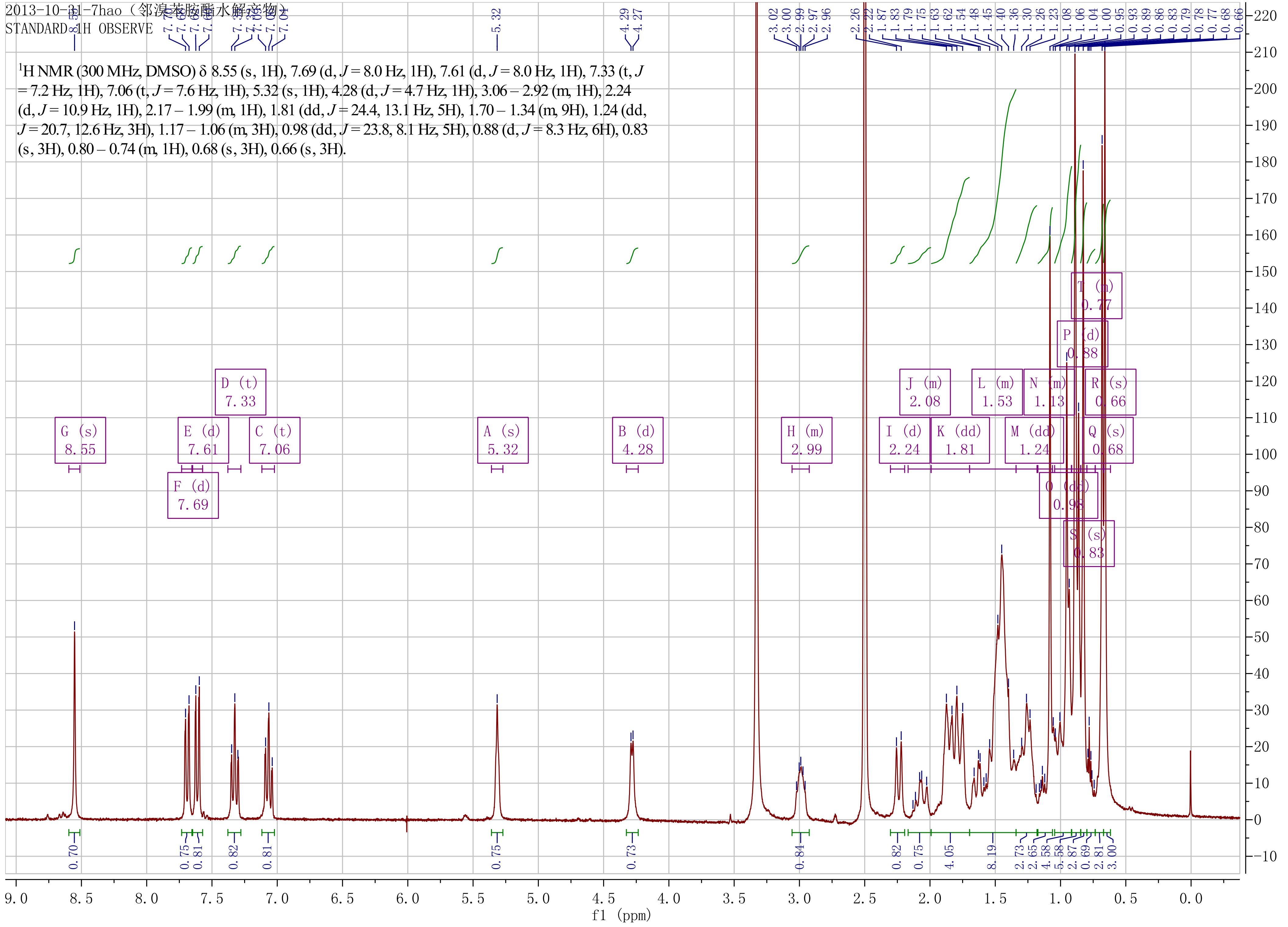

^{1}H NMR (300 MHz, DMSO) δ 8.55 (s, 1H), 7.69 (d, J = 8.0 Hz, 1H), 7.61 (d, J = 8.0 Hz, 1H), 7.33 (t, J = 7.2 Hz, 1H), 7.06 (t, J = 7.6 Hz, 1H), 5.32 (s, 1H), 4.28 (d, J = 4.7 Hz, 1H), 3.06 – 2.92 (m, 1H), 2.24 (d, J = 10.9 Hz, 1H), 2.17 – 1.99 (m, 1H), 1.81 (dd, J = 24.4, 13.1 Hz, 5H), 1.70 – 1.34 (m, 9H), 1.24 (dd, J = 20.7, 12.6 Hz, 3H), 1.17 – 1.06 (m, 3H), 0.98 (dd, J = 23.8, 8.1 Hz, 5H), 0.88 (d, J = 8.3 Hz, 6H), 0.83 (s, 3H), 0.80 – 0.74 (m, 1H), 0.68 (s, 3H), 0.66 (s, 3H).

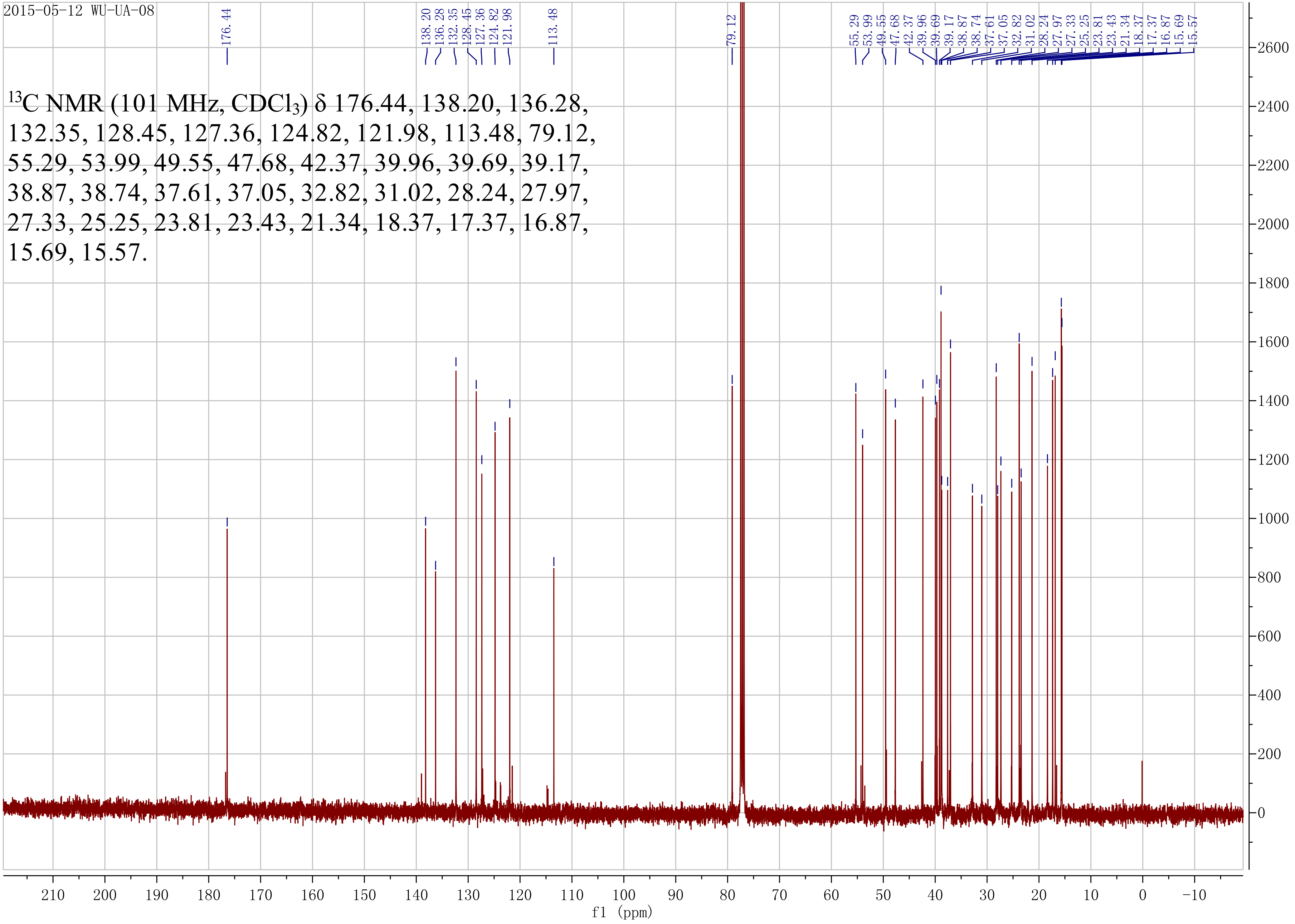

^{13}C NMR (101 MHz, $CDCl_3$) δ 176.44, 138.20, 136.28, 132.35, 128.45, 127.36, 124.82, 121.98, 113.48, 79.12, 55.29, 53.99, 49.55, 47.68, 42.37, 39.96, 39.69, 39.17, 38.87, 38.74, 37.61, 37.05, 32.82, 31.02, 28.24, 27.97, 27.33, 25.25, 23.81, 23.43, 21.34, 18.37, 17.37, 16.87, 15.69, 15.57.

Compound **7b:** ***N-[3β-Hydroxy-urs-12-en-28-oyl]-p-fluoroaniline***

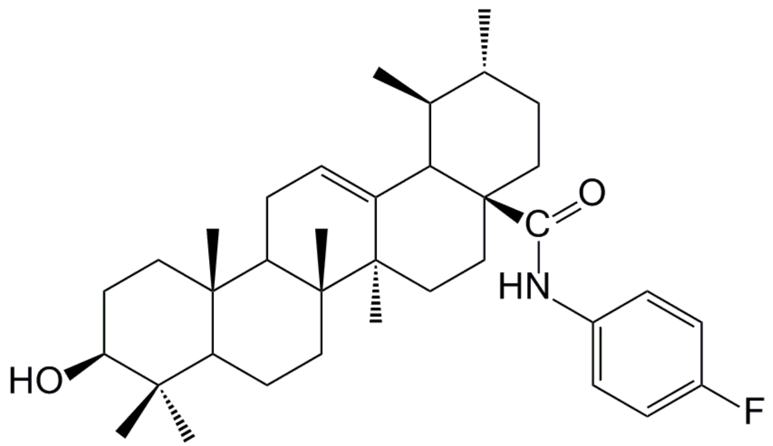

Figure 6. The structure of compound **7b**.

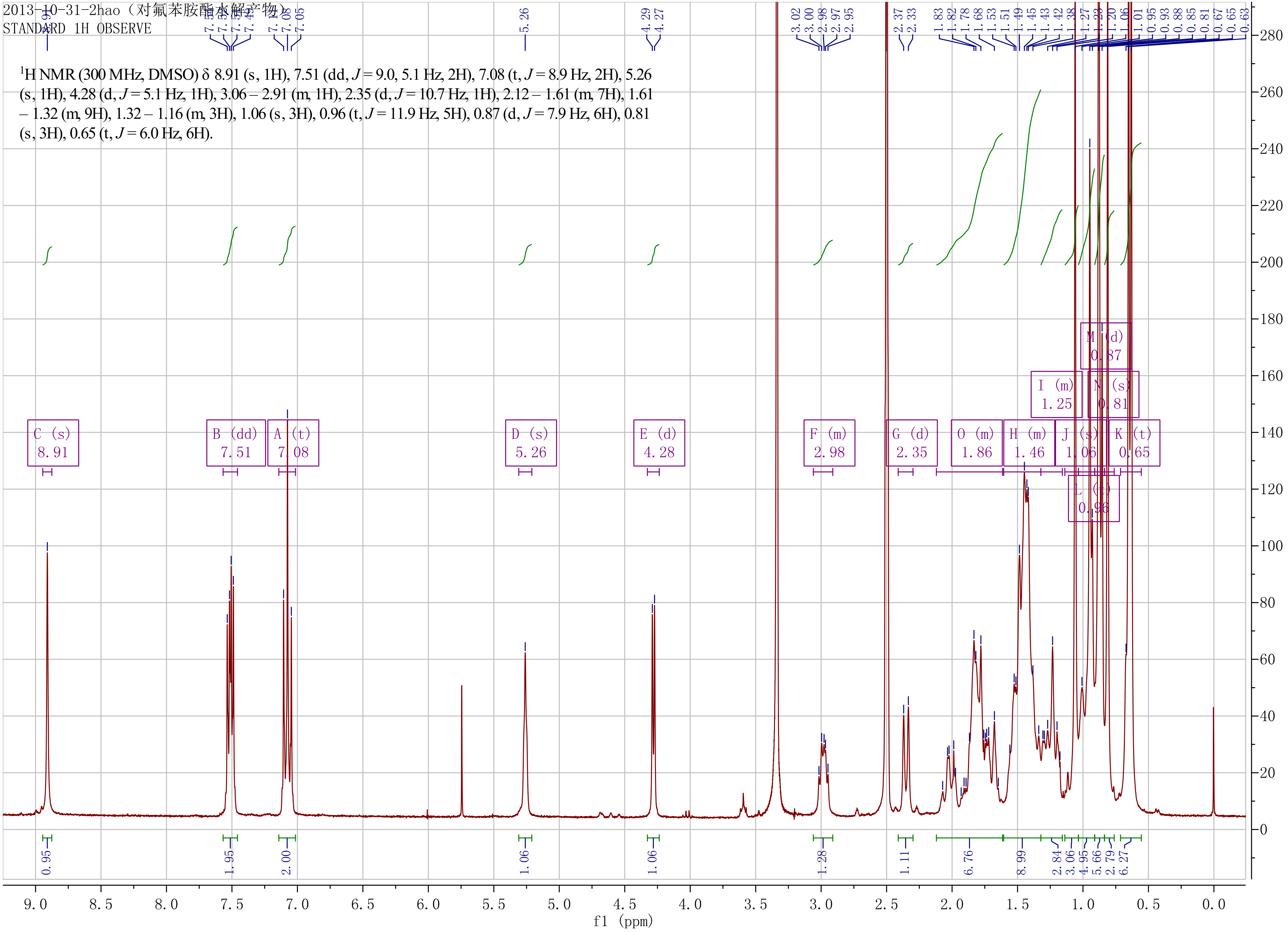
2013-10-31-2hao（对氟苯胺酯水解产物）
STANDARD 1H OBSERVE
^{1}H NMR (300 MHz, DMSO) δ 8.91 (s, 1H), 7.51 (dd, J = 9.0, 5.1 Hz, 2H), 7.08 (t, J = 8.9 Hz, 2H), 5.26 (s, 1H), 4.28 (d, J = 5.1 Hz, 1H), 3.06 – 2.91 (m, 1H), 2.35 (d, J = 10.7 Hz, 1H), 2.12 – 1.61 (m, 7H), 1.61 – 1.32 (m, 9H), 1.32 – 1.16 (m, 3H), 1.06 (s, 3H), 0.96 (t, J = 11.9 Hz, 5H), 0.87 (d, J = 7.9 Hz, 6H), 0.81 (s, 3H), 0.65 (t, J = 6.0 Hz, 6H).
f1 (ppm)

2015-05-12 WU-UA-03

^{13}C NMR (101 MHz, $CDCl_3$) δ 176.42, 160.52, 158.11, 140.33, 134.33, 134.31, 126.26, 121.50, 121.42, 115.77, 115.55, 79.07, 55.25, 54.44, 48.69, 47.65, 42.79, 40.04, 39.69, 39.26, 38.88, 38.81, 37.17, 37.04, 32.87, 31.02, 28.25, 28.04, 27.32, 25.25, 23.70, 23.43, 21.32, 18.34, 17.40, 17.01, 15.71, 15.65.

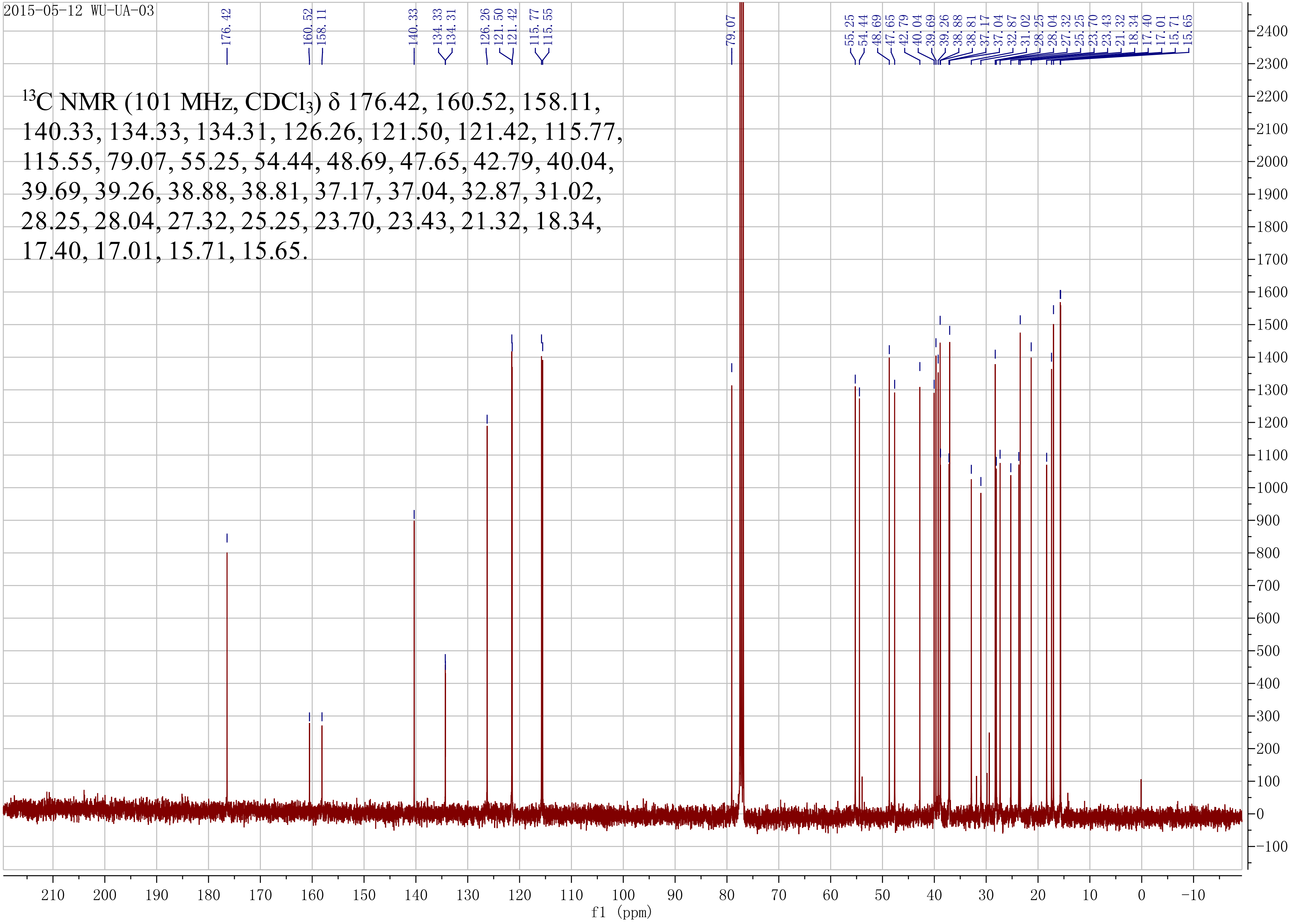

Compound **8b:** ***N-[3β-Hydroxy-urs-12-en-28-oyl]-p-chloroaniline***

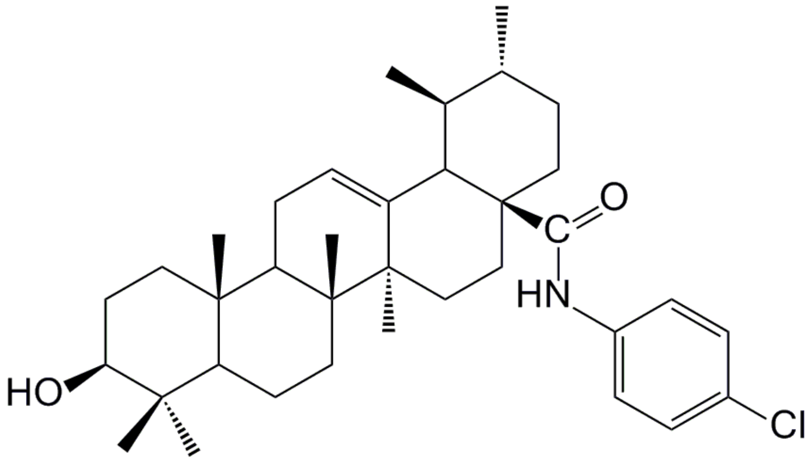

Figure 7. The structure of compound **8b**.

2013-10-31-3hao（对氯苯胺酯水解产物）
STANDARD 1H OBSERVE

^{1}H NMR (300 MHz, DMSO) δ 8.98 (s, 1H), 7.56 (d, J = 8.9 Hz, 2H), 7.29 (d, J = 8.8 Hz, 2H), 5.26 (s, 1H), 4.27 (s, 1H), 3.05 – 2.91 (m, 1H), 2.36 (d, J = 10.7 Hz, 1H), 2.03 (td, J = 14.2, 3.8 Hz, 1H), 1.94 – 1.62 (m, 5H), 1.59 – 1.32 (m, 9H), 1.24 (dd, J = 23.0, 11.4 Hz, 3H), 1.06 (s, 3H), 0.96 (t, J = 11.8 Hz, 5H), 0.87 (d, J = 7.4 Hz, 6H), 0.81 (s, 3H), 0.66 (d, J = 5.9 Hz, 4H), 0.61 (s, 3H).

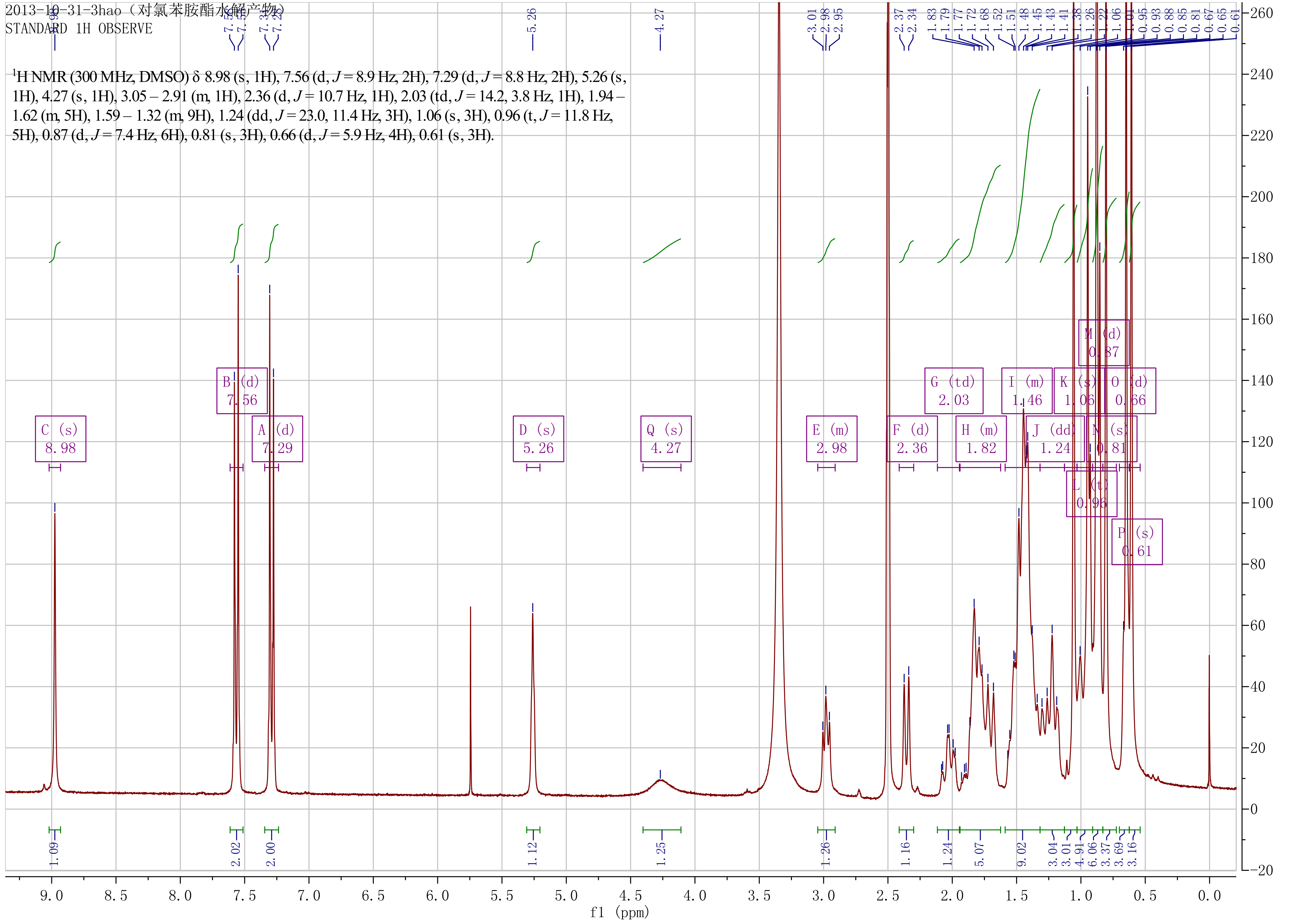

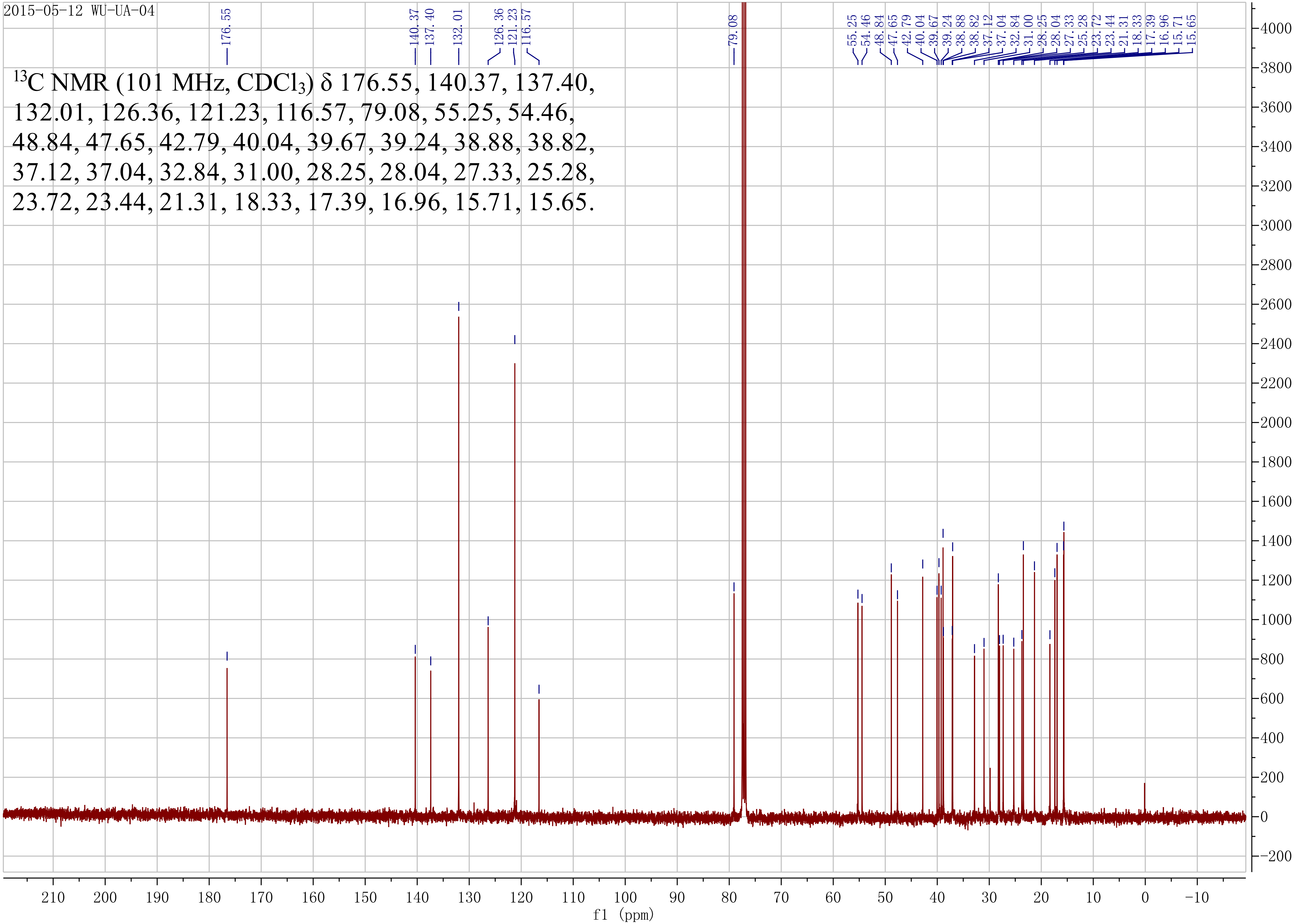

^{13}C NMR (101 MHz, $CDCl_3$) δ 176.55, 140.37, 137.40, 132.01, 126.36, 121.23, 116.57, 79.08, 55.25, 54.46, 48.84, 47.65, 42.79, 40.04, 39.67, 39.24, 38.88, 38.82, 37.12, 37.04, 32.84, 31.00, 28.25, 28.04, 27.33, 25.28, 23.72, 23.44, 21.31, 18.33, 17.39, 16.96, 15.71, 15.65.

Compound **9b:** ***N-[3β-Hydroxy-urs-12-en-28-oyl]-p-bromoaniline***

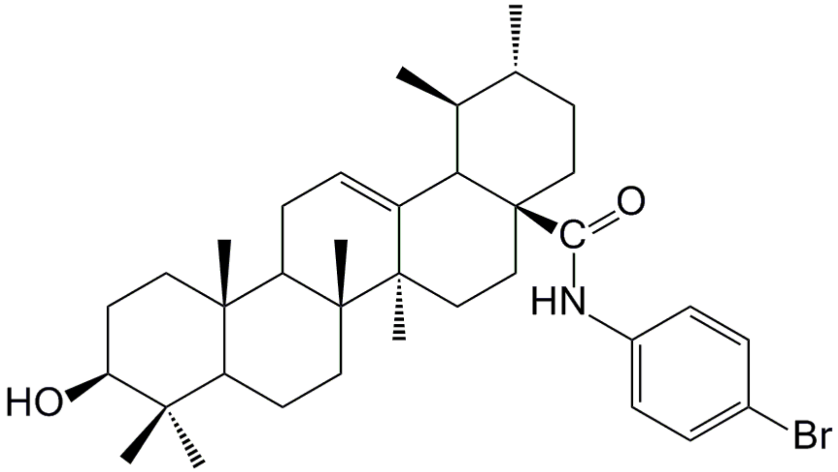

Figure 8. The structure of compound **9b**.

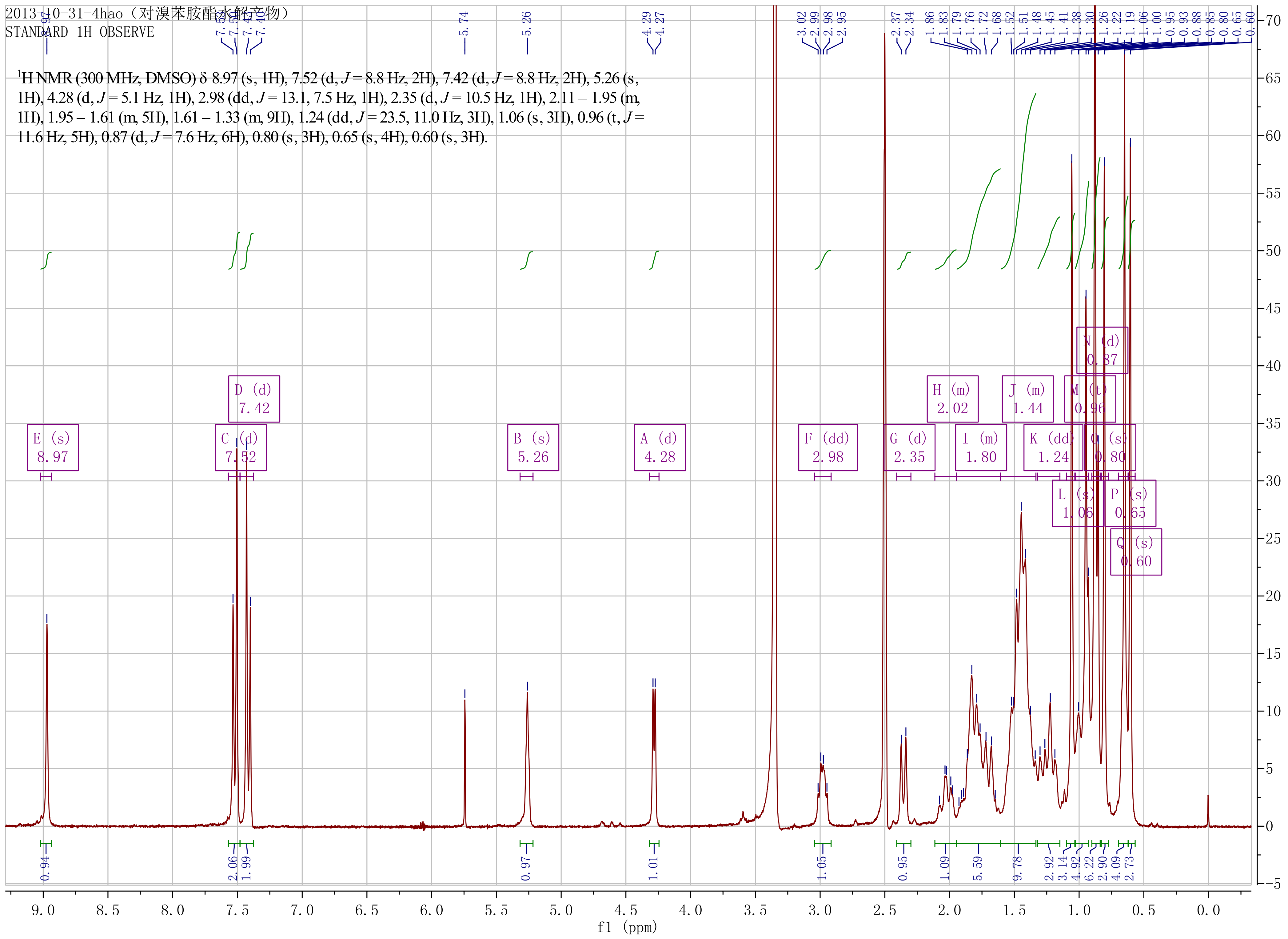
2013-10-31-4hao（对溴苯胺酯水解产物）
STANDARD 1H OBSERVE
^{1}H NMR (300 MHz, DMSO) δ 8.97 (s, 1H), 7.52 (d, J = 8.8 Hz, 2H), 7.42 (d, J = 8.8 Hz, 2H), 5.26 (s, 1H), 4.28 (d, J = 5.1 Hz, 1H), 2.98 (dd, J = 13.1, 7.5 Hz, 1H), 2.35 (d, J = 10.5 Hz, 1H), 2.11 – 1.95 (m, 1H), 1.95 – 1.61 (m, 5H), 1.61 – 1.33 (m, 9H), 1.24 (dd, J = 23.5, 11.0 Hz, 3H), 1.06 (s, 3H), 0.96 (t, J = 11.6 Hz, 5H), 0.87 (d, J = 7.6 Hz, 6H), 0.80 (s, 3H), 0.65 (s, 4H), 0.60 (s, 3H).
f1 (ppm)

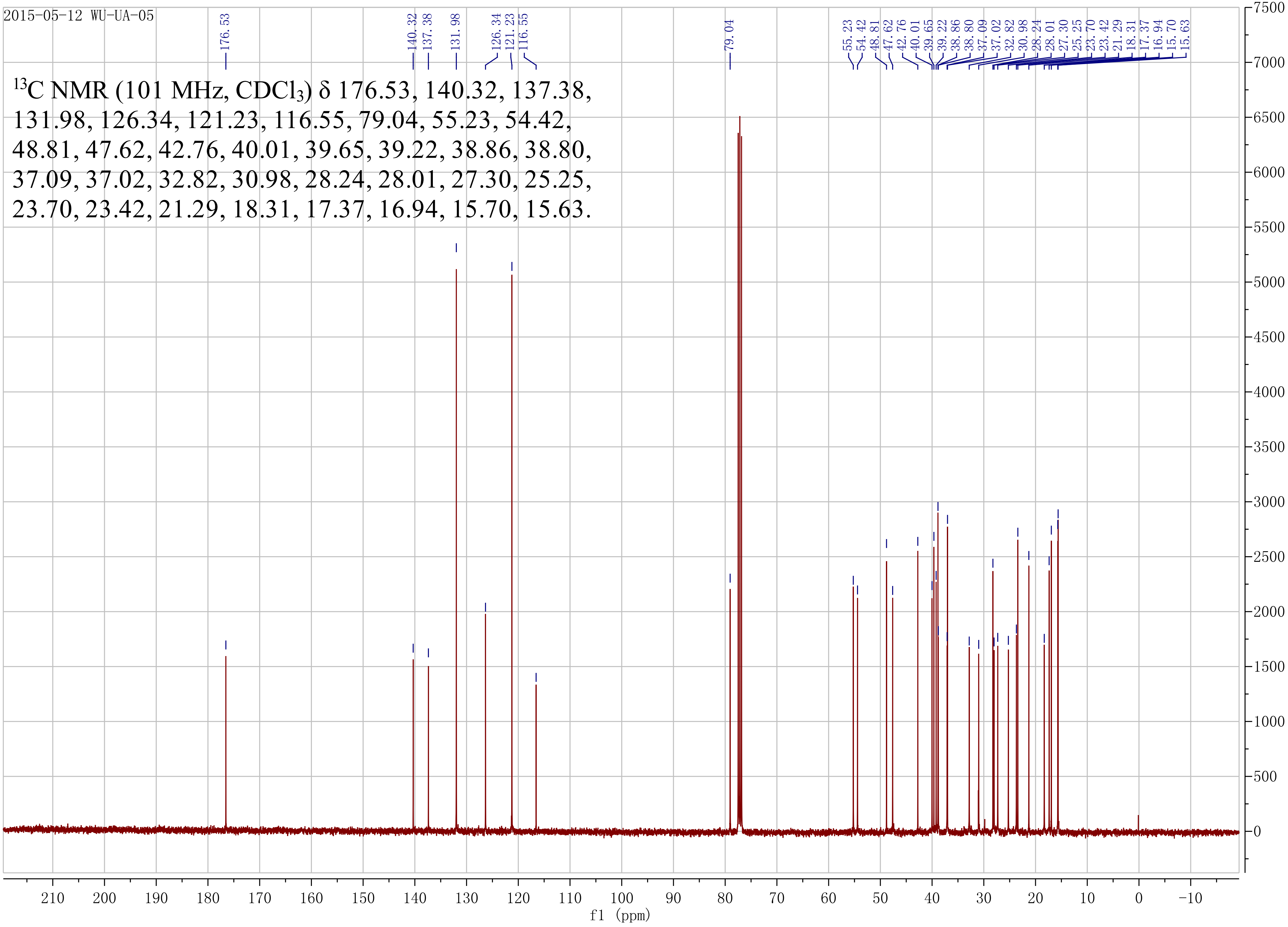
2015-05-12 WU-UA-05
13C NMR (101 MHz, CDCl3) δ 176.53, 140.32, 137.38, 131.98, 126.34, 121.23, 116.55, 79.04, 55.23, 54.42, 48.81, 47.62, 42.76, 40.01, 39.65, 39.22, 38.86, 38.80, 37.09, 37.02, 32.82, 30.98, 28.24, 28.01, 27.30, 25.25, 23.70, 23.42, 21.29, 18.31, 17.37, 16.94, 15.70, 15.63.
f1 (ppm)

Compound **10b:** ***N-[3β-Hydroxy-urs-12-en-28-oyl]-p-methoxylaniline***

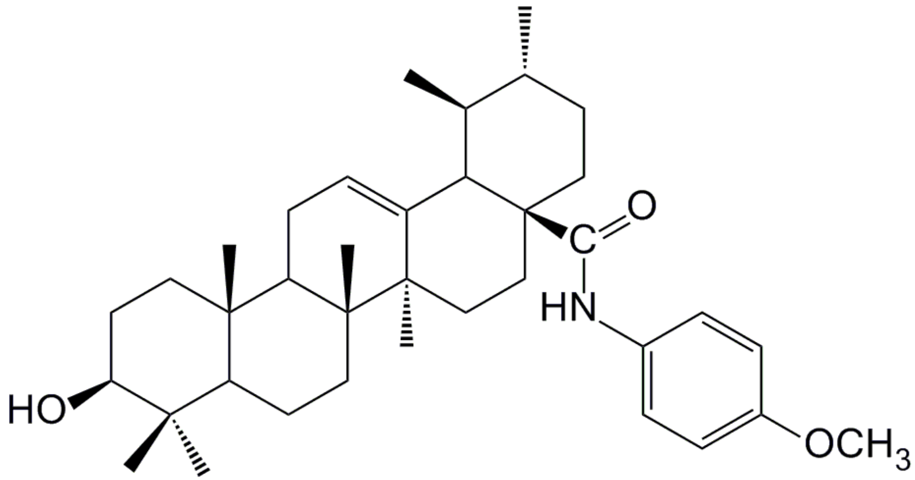

Figure 9. The structure of compound **10b**.

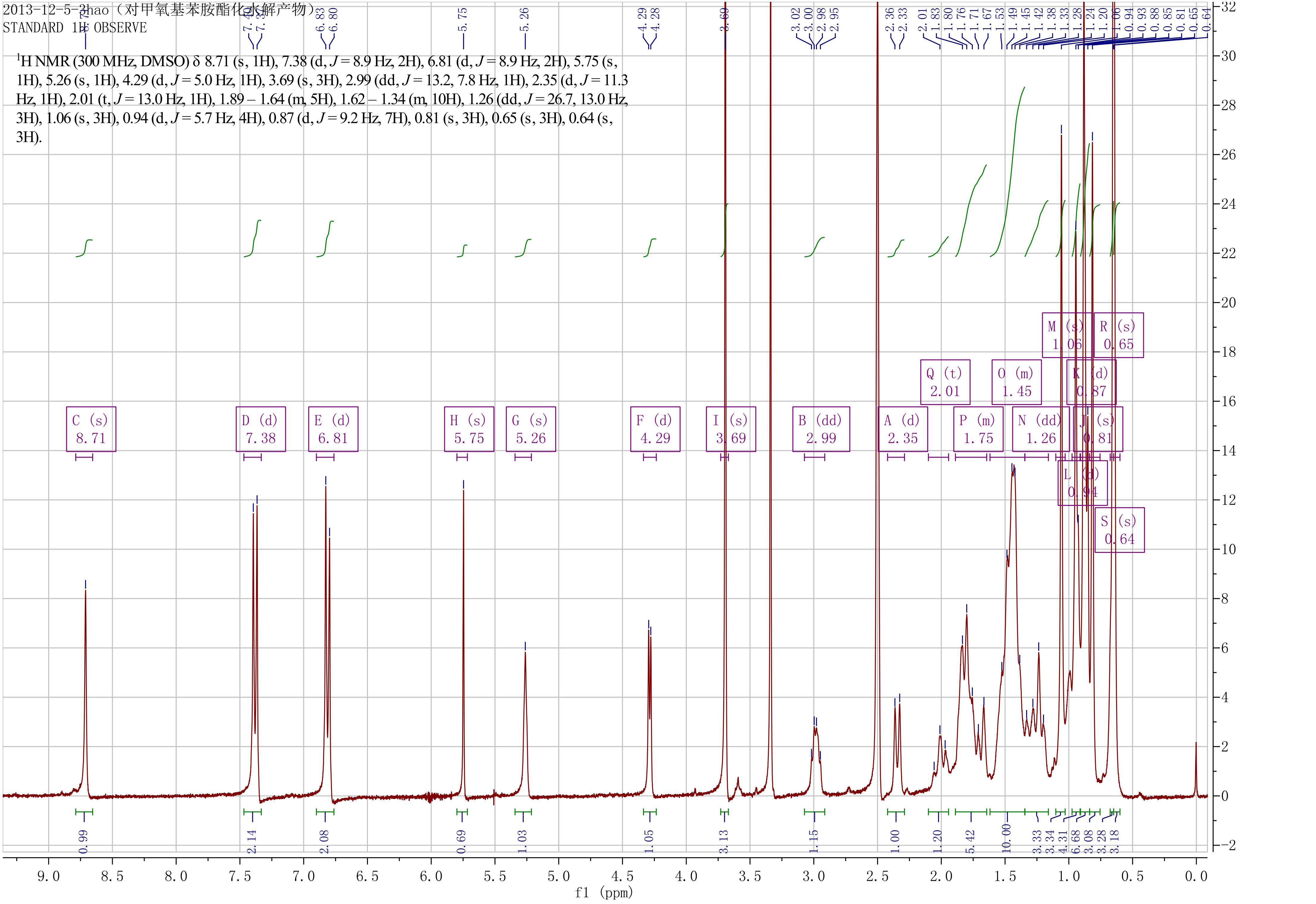

^{1}H NMR (300 MHz, DMSO) δ 8.71 (s, 1H), 7.38 (d, *J* = 8.9 Hz, 2H), 6.81 (d, *J* = 8.9 Hz, 2H), 5.75 (s, 1H), 5.26 (s, 1H), 4.29 (d, *J* = 5.0 Hz, 1H), 3.69 (s, 3H), 2.99 (dd, *J* = 13.2, 7.8 Hz, 1H), 2.35 (d, *J* = 11.3 Hz, 1H), 2.01 (t, *J* = 13.0 Hz, 1H), 1.89 – 1.64 (m, 5H), 1.62 – 1.34 (m, 10H), 1.26 (dd, *J* = 26.7, 13.0 Hz, 3H), 1.06 (s, 3H), 0.94 (d, *J* = 5.7 Hz, 4H), 0.87 (d, *J* = 9.2 Hz, 7H), 0.81 (s, 3H), 0.65 (s, 3H), 0.64 (s, 3H).

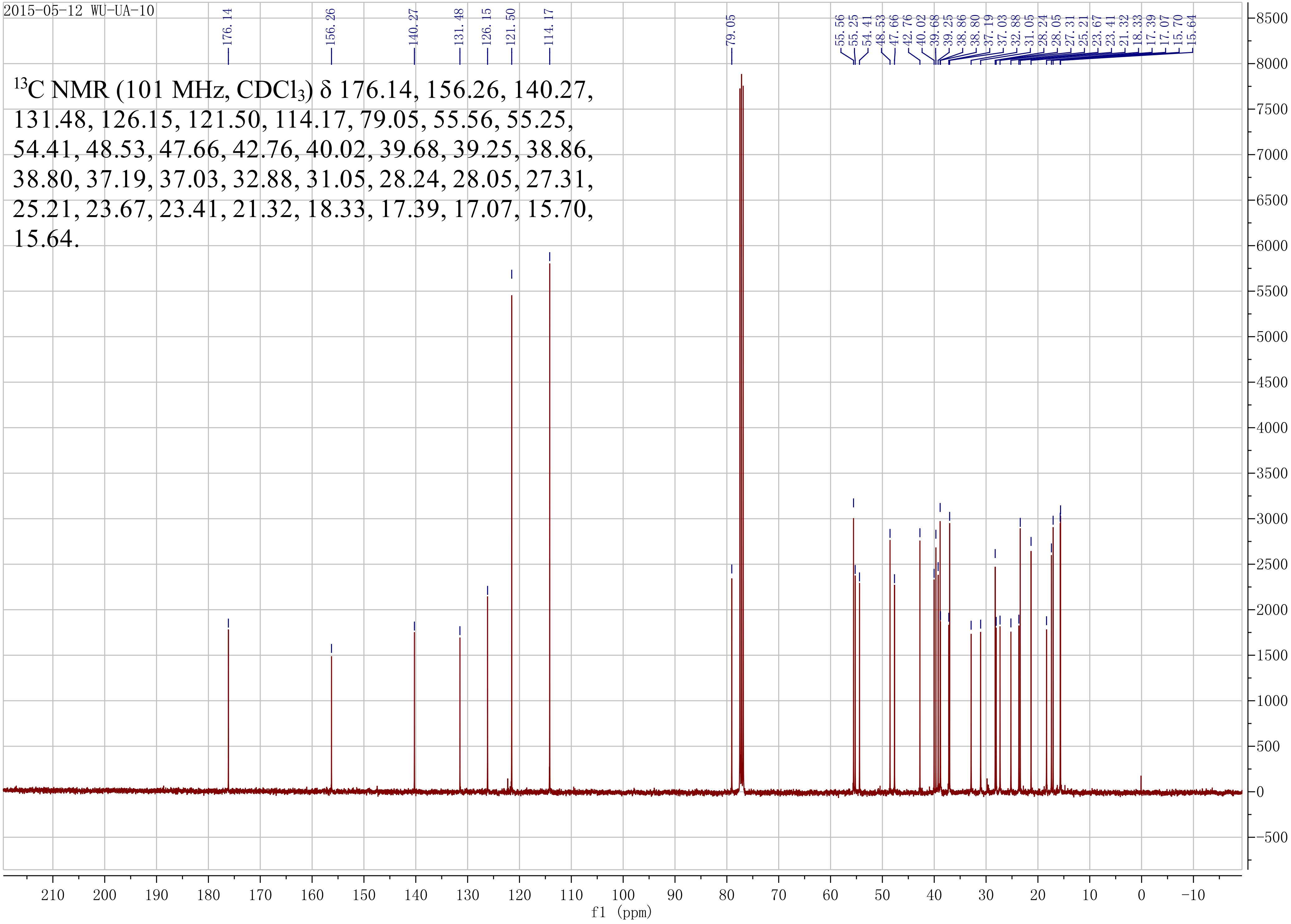
2015-05-12 WU-UA-10
^{13}C NMR (101 MHz, CDCl$_3$) δ 176.14, 156.26, 140.27, 131.48, 126.15, 121.50, 114.17, 79.05, 55.56, 55.25, 54.41, 48.53, 47.66, 42.76, 40.02, 39.68, 39.25, 38.86, 38.80, 37.19, 37.03, 32.88, 31.05, 28.24, 28.05, 27.31, 25.21, 23.67, 23.41, 21.32, 18.33, 17.39, 17.07, 15.70, 15.64.
f1 (ppm)

Compound **11:** ***3-Oxo-urs-12-en-28-oic acid***

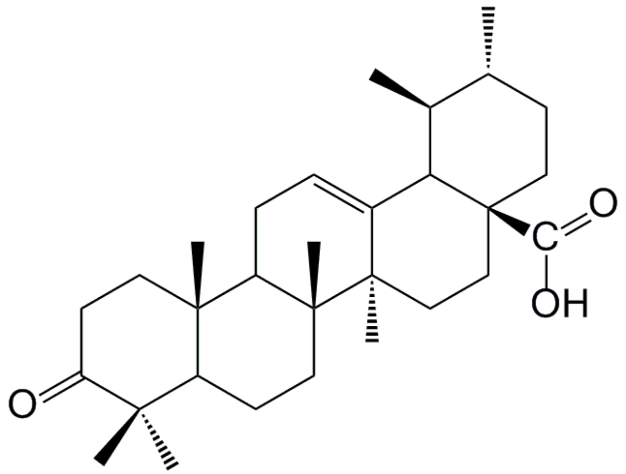

Figure 10. The structure of compound **11**.

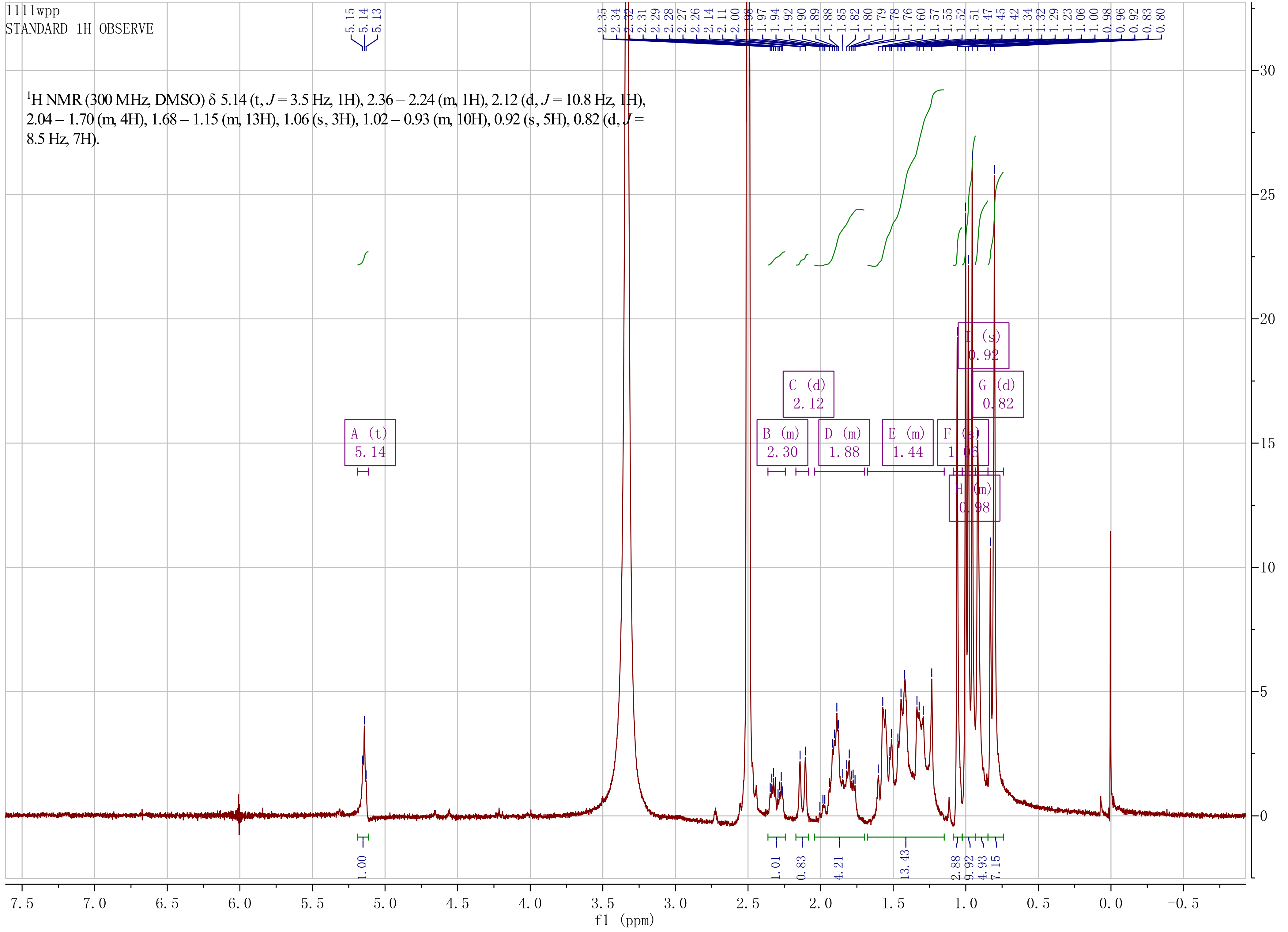
1111wpp
STANDARD 1H OBSERVE
1H NMR (300 MHz, DMSO) δ 5.14 (t, J = 3.5 Hz, 1H), 2.36 – 2.24 (m, 1H), 2.12 (d, J = 10.8 Hz, 1H), 2.04 – 1.70 (m, 4H), 1.68 – 1.15 (m, 13H), 1.06 (s, 3H), 1.02 – 0.93 (m, 10H), 0.92 (s, 5H), 0.82 (d, J = 8.5 Hz, 7H).
A (t) 5.14
B (m) 2.30
C (d) 2.12
D (m) 1.88
E (m) 1.44
F (s) 1.06
G (d) 0.82
H (m) 0.98
I (s) 0.92
1.00
1.01
0.83
4.21
13.43
2.88
9.92
4.93
7.15
f1 (ppm)

2015-05-12 WU-UA-yanghua

^{13}C NMR (101 MHz, $CDCl_3$) δ 217.93, 184.02, 138.17, 125.71, 55.38, 52.71, 48.16, 47.54, 46.90, 42.20, 39.61, 39.42, 39.17, 38.95, 36.85, 36.82, 34.30, 32.58, 30.74, 28.12, 26.68, 24.18, 23.67, 23.56, 21.58, 21.29, 19.68, 17.15, 17.12, 15.36.

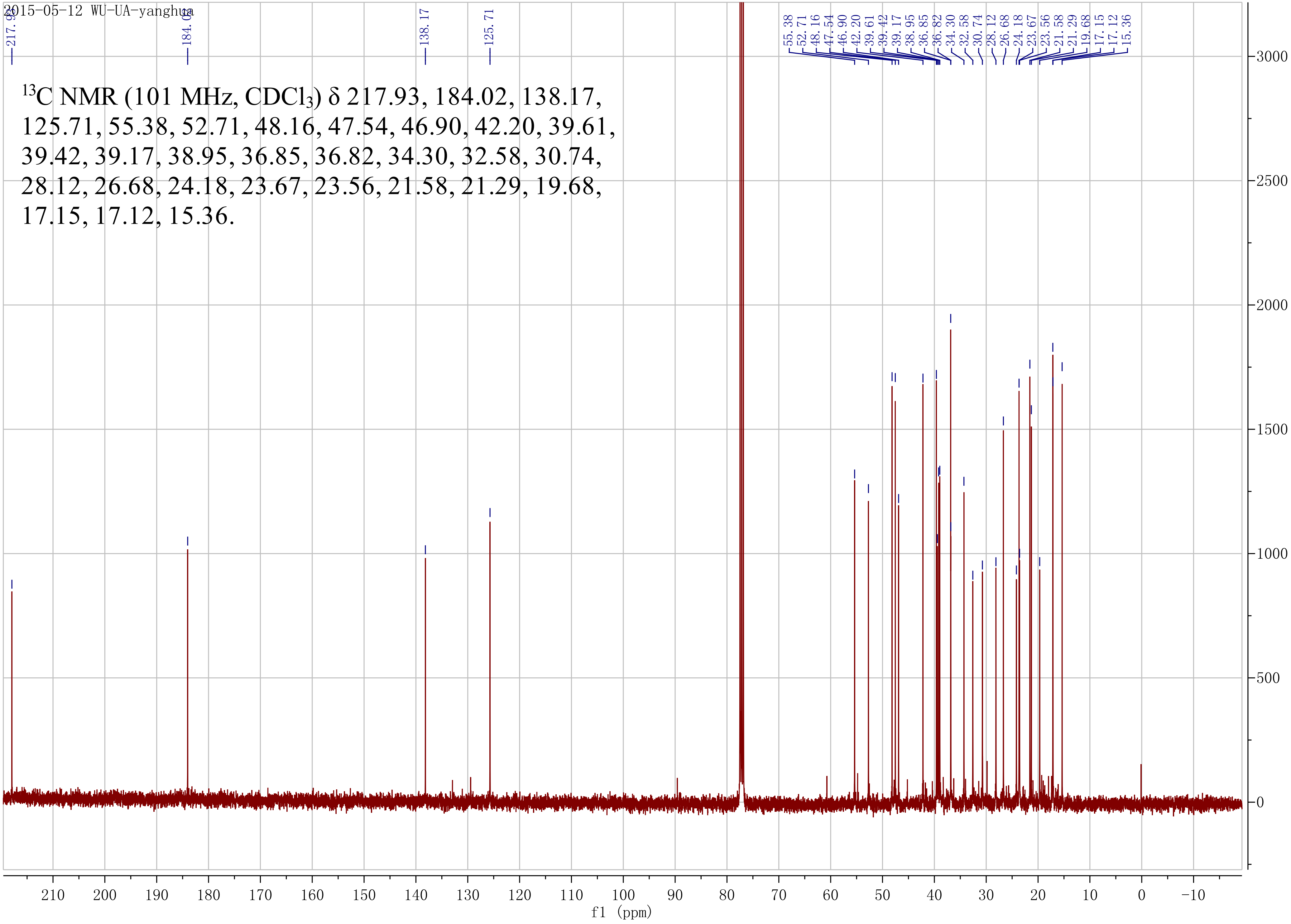

Supplement: S1 File — (PDF) [file pone.0138767.s001.pdf]
