# Supplementary material for: Synthesis and Evaluation of Novel Triterpene Analogues of Ursolic Acid as Potential Antidiabetic Agent
Source: PLoS One. 2015 Sep 25;10(9):e0138767. doi: 10.1371/journal.pone.0138767 (PMC4583267; doi:10.1371/journal.pone.0138767)

Synthesis and evaluation of novel triterpene analogues of ursolic acid as potential antidiabetic agent

Pan-Pan Wu, Tian-Ming Huang, Qing-Qing Hu, An-Ming Cheng, Zheng-Yun Jiang, Luo-Ying Jiao, Su-Qing Zhao*, Kun Zhang*

Supplementary Data

Copies of mass spectrum of compounds **10a**, **3b-10b** and **11**.

N-[3β-Acetoxy-urs-12-en-28-oyl]-p-methoxyamiline

(Compound **10a**)

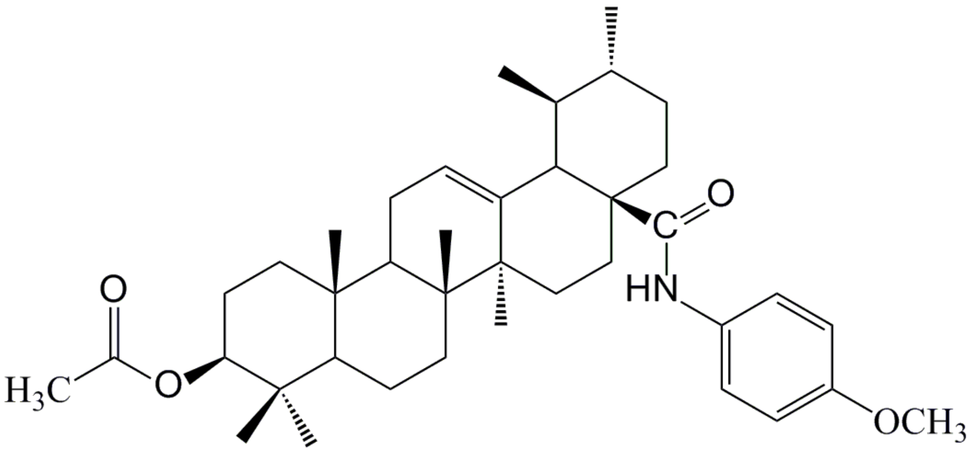

Figure 1. The structure of compound **10a**.

Mass spectrum of compound **10a**:

ESI-MS *m/z* 602.2 $[M-H]^-$

HRMS *m/z* 604.4383 $[M+H]^+$

Calcd for $C_{39}H_{58}NO_4$: 604.4360

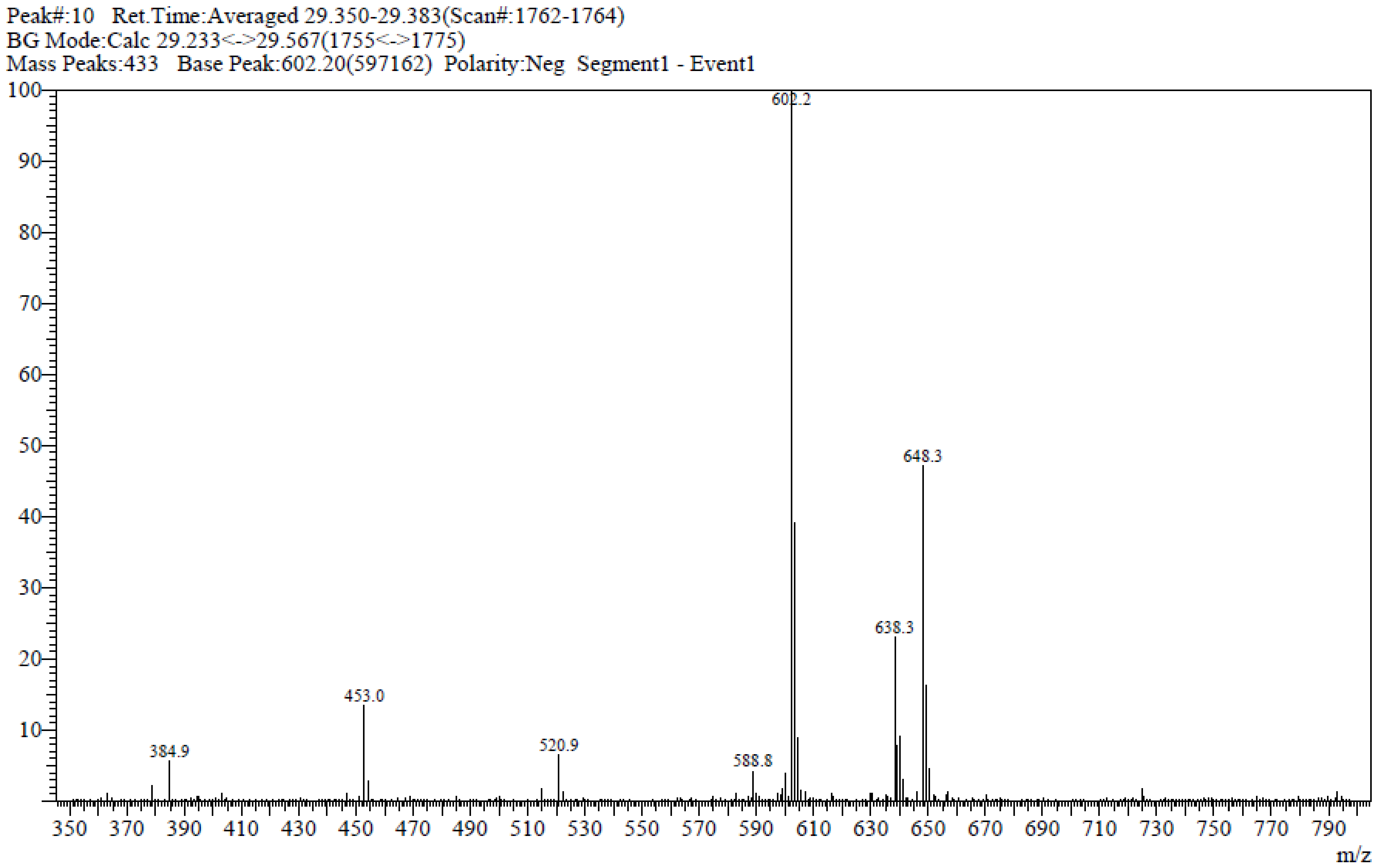

Peak#:10 Ret.Time:Averaged 29.350-29.383(Scan#:1762-1764)
BG Mode:Calc 29.233<->29.567(1755<->1775)
Mass Peaks:433 Base Peak:602.20(597162) Polarity:Neg Segment1 - Event1
602.2
648.3
638.3
453.0
384.9
520.9
588.8
100
90
80
70
60
50
40
30
20
10
350 370 390 410 430 450 470 490 510 530 550 570 590 610 630 650 670 690 710 730 750 770 790
m/z

Generic Display Report

Analysis Info

Analysis Name	D:\Data\201404\140414-01\140414-01-1_P1-A-1_01_284.d	Acquisition Date	4/15/2014 3:33:49 PM
Method	esi_pos_50-1000_with calibration_for 1min.m	Operator	BDAL@DE
Sample Name	140414-01-1	Instrument	maXis impact
Comment			

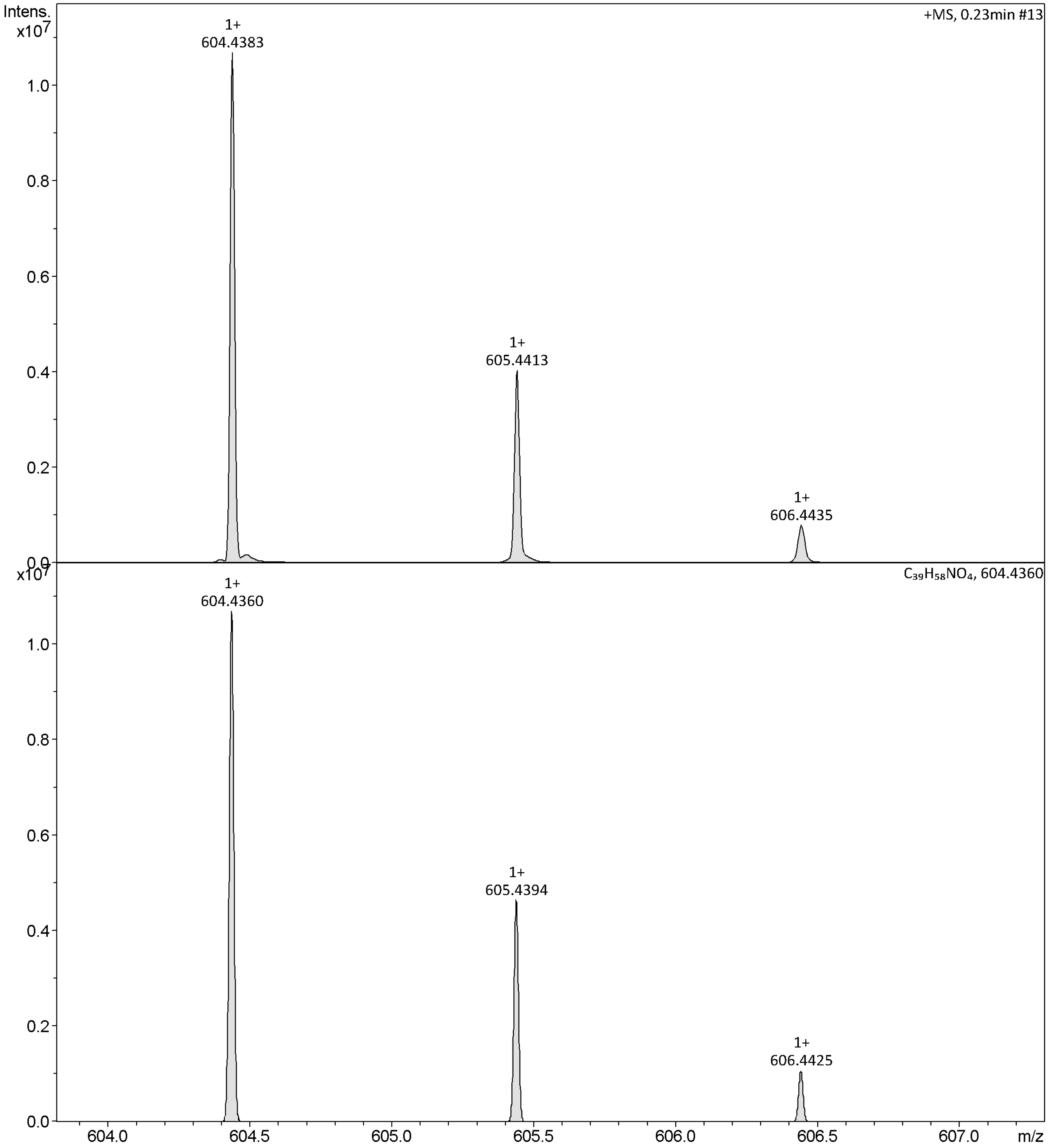

N-[3β-Hydroxy-urs-12-en-28-oyl]-aminobenzene

(Compound **3b**)

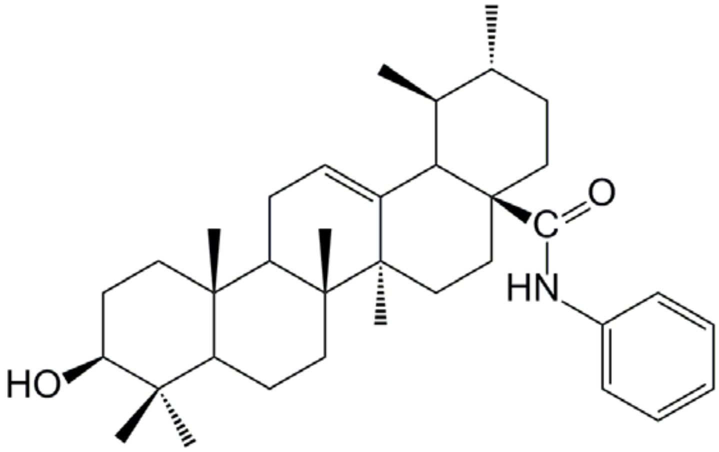

Figure 2. The structure of compound **3b**.

Mass spectrum of compound **3b**:

ESI-MS *m/z* 530.3 $[M-H]^-$

HRMS *m/z* 532.4163 $[M+H]^+$

Calcd for $C_{36}H_{54}NO_2$: 532.4149

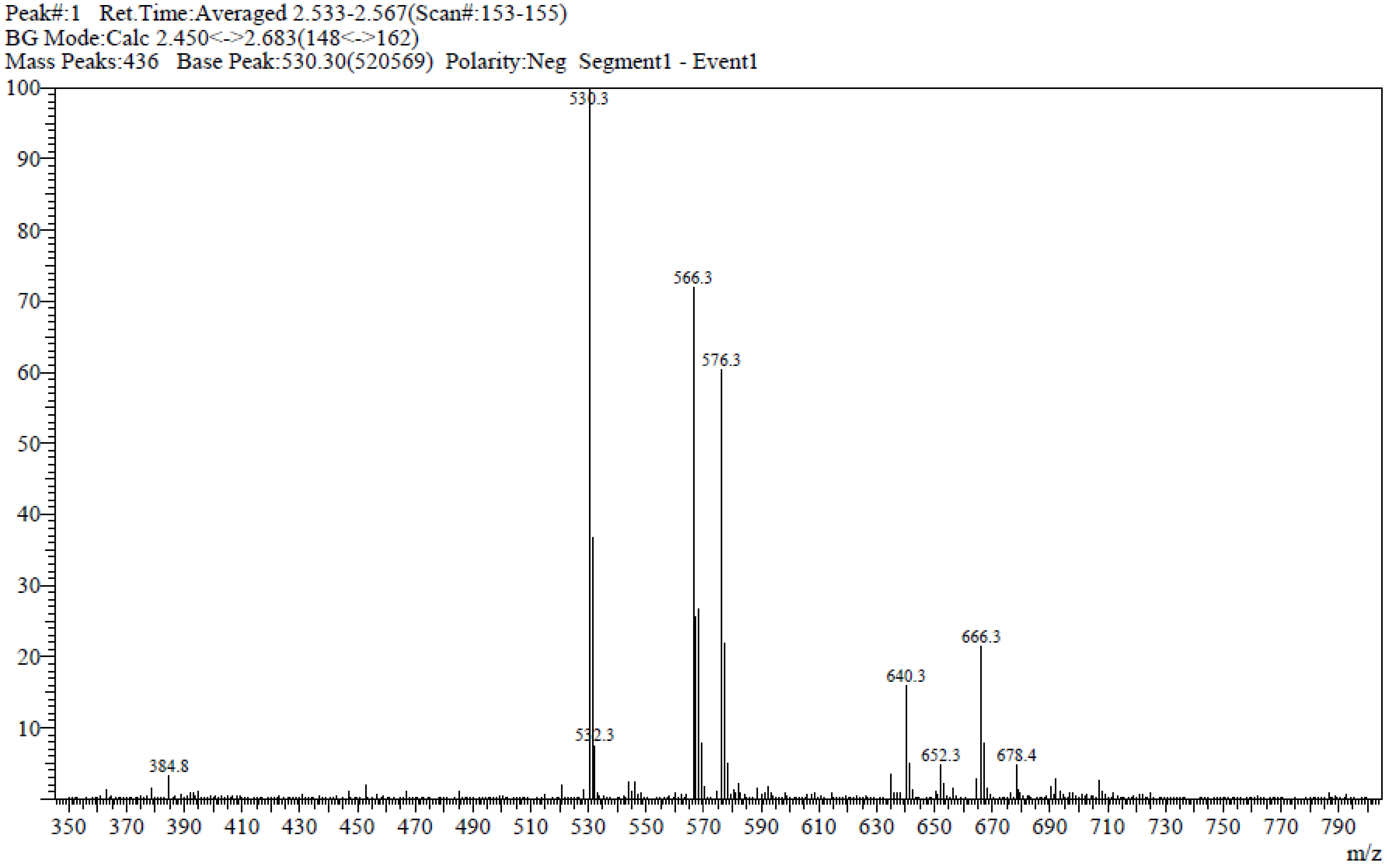
Peak#:1 Ret.Time:Averaged 2.533-2.567(Scan#:153-155)
BG Mode:Calc 2.450<->2.683(148<->162)
Mass Peaks:436 Base Peak:530.30(520569) Polarity:Neg Segment1 - Event1
530.3
566.3
576.3
666.3
640.3
532.3
384.8
652.3
678.4
m/z

Generic Display Report

Analysis Info

Analysis Name	D:\Data\201404\140414-01\140414-01-3_P1-A-3_01_286.d	Acquisition Date	4/15/2014 3:36:53 PM
Method	esi_pos_50-1000_with calibration_for 1min.m	Operator	BDAL@DE
Sample Name	140414-01-3	Instrument	maXis impact
Comment			

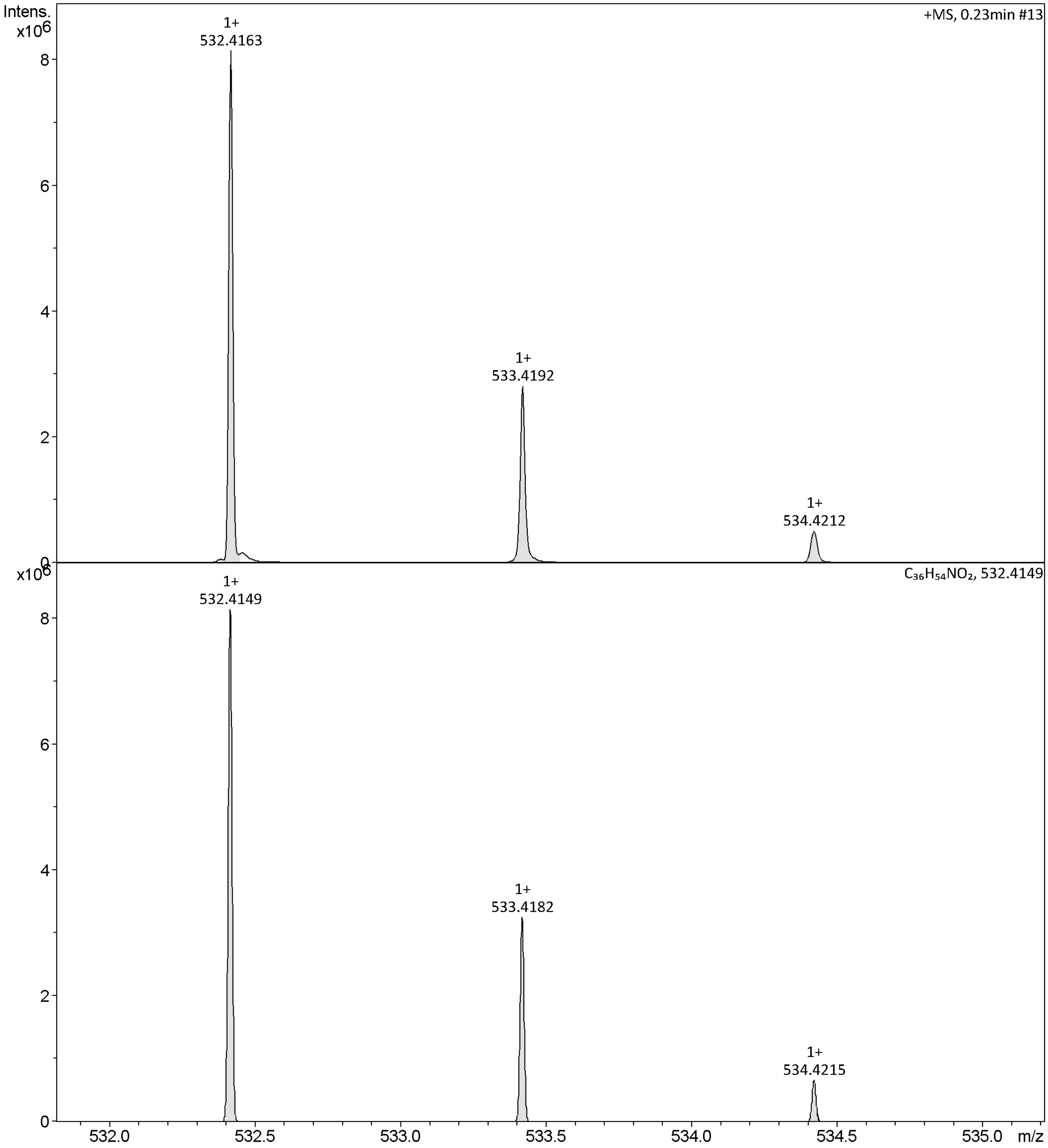

***N*-[3β-Hydroxy-urs-12-en-28-oyl]-*o*-fluoroaniline**

(Compound **4b**)

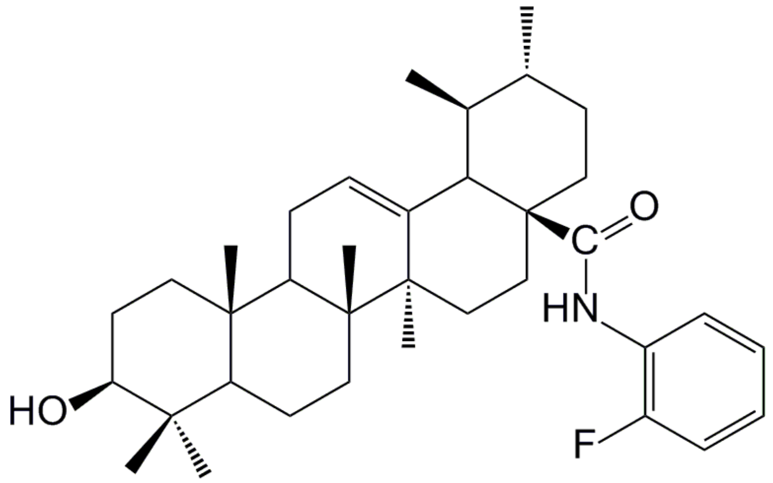

Figure 3. The structure of compound **4b**.

Mass spectrum of compound **4b**:

ESI-MS *m/z* 548.2 $[M-H]^-$

HRMS *m/z* 550.4067 $[M+H]^+$

Calcd for $C_{36}H_{53}FNO_2$: 550.4055

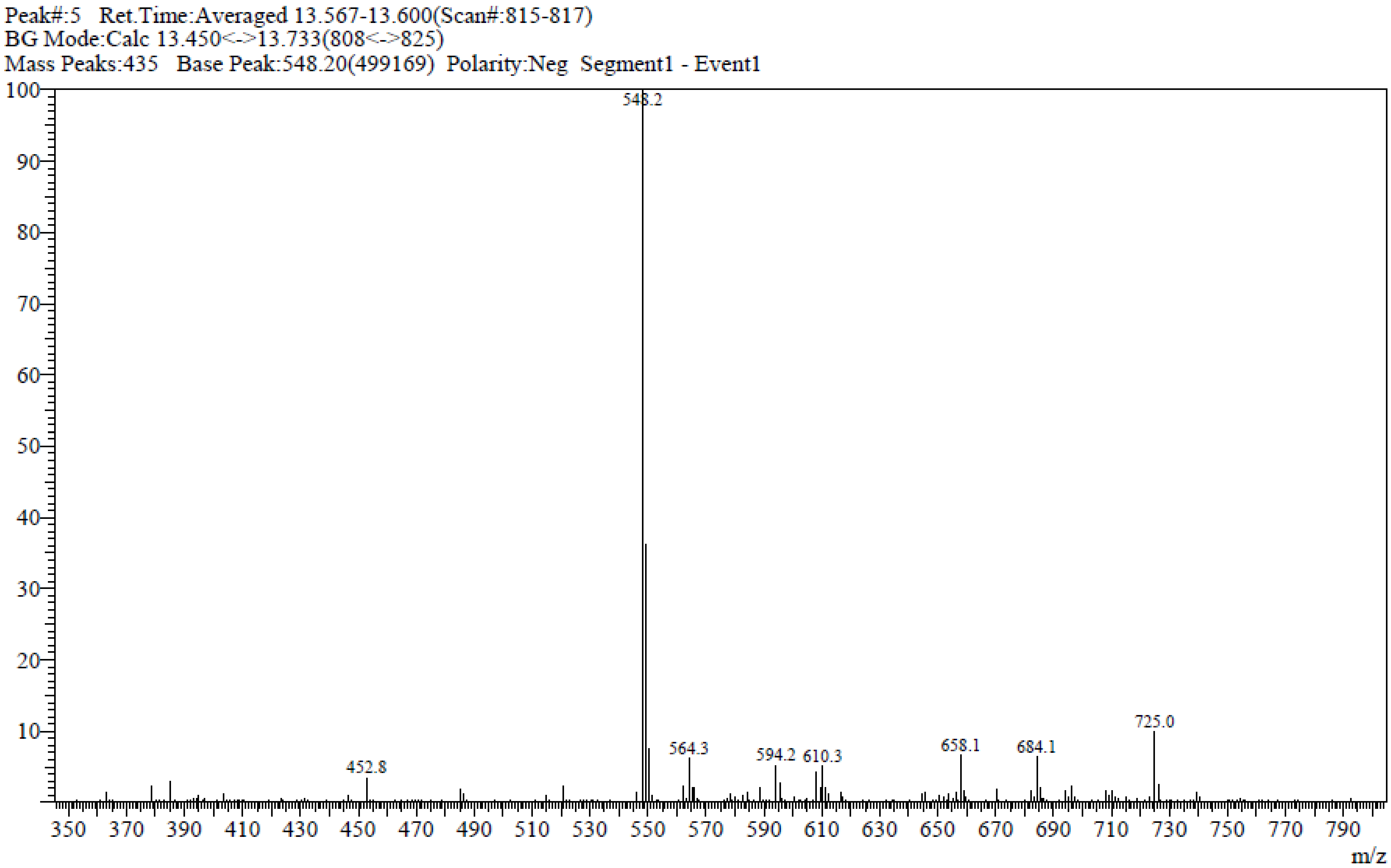
Peak#:5 Ret.Time:Averaged 13.567-13.600(Scan#:815-817)
BG Mode:Calc 13.450<->13.733(808<->825)
Mass Peaks:435 Base Peak:548.20(499169) Polarity:Neg Segment1 - Event1
548.2
452.8
564.3
594.2
610.3
658.1
684.1
725.0
m/z

Generic Display Report

Analysis Info

Analysis Name	D:\Data\201404\140414-01\140414-01-7_P1-A-7_01_290.d	Acquisition Date	4/15/2014 3:43:01 PM
Method	esi_pos_50-1000_with calibration_for 1min.m	Operator	BDAL@DE
Sample Name	140414-01-7	Instrument	maXis impact
Comment			

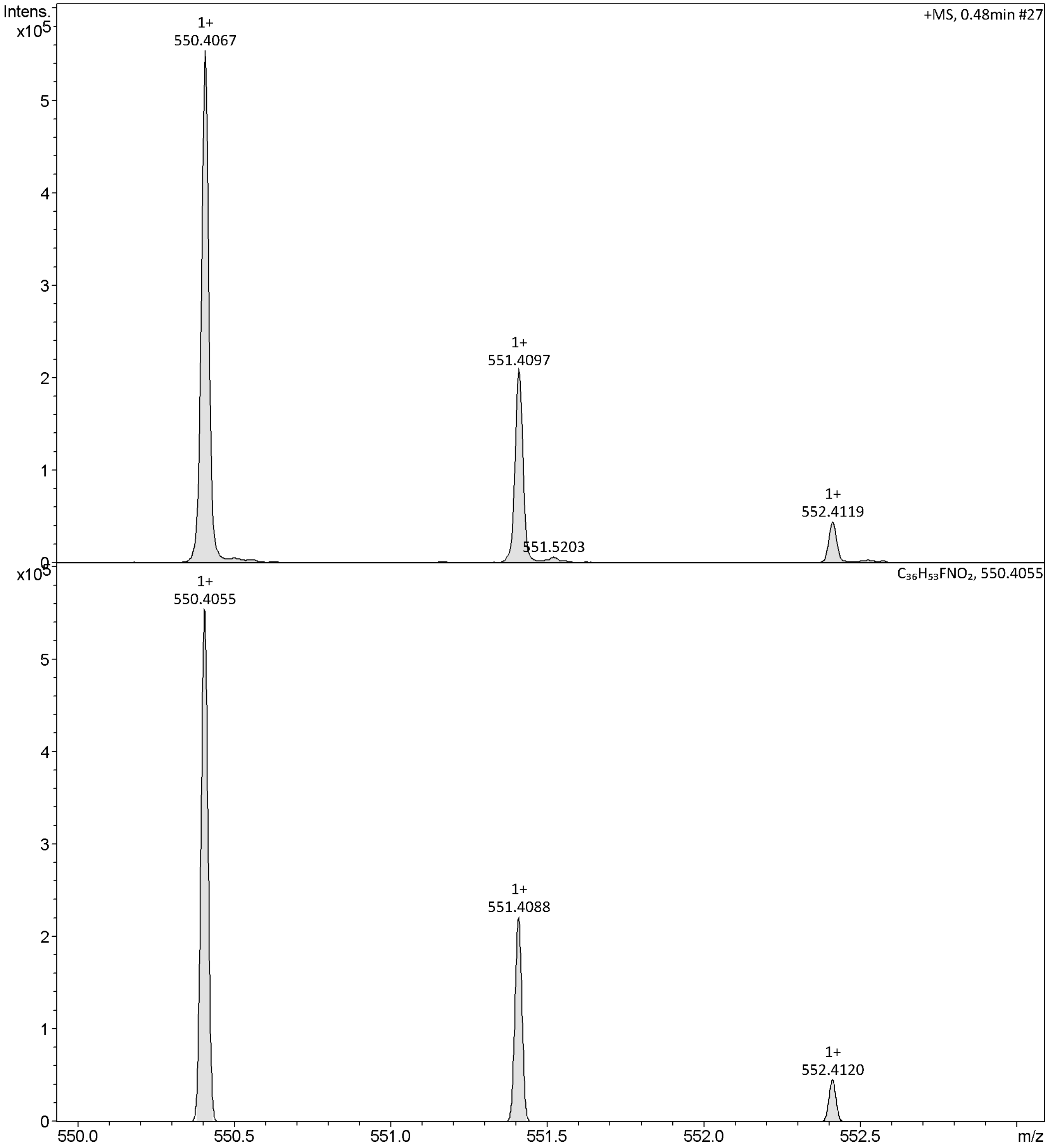

N-[3β-Hydroxy-urs-12-en-28-oyl]-o-chloroaniline

(Compound **5b**)

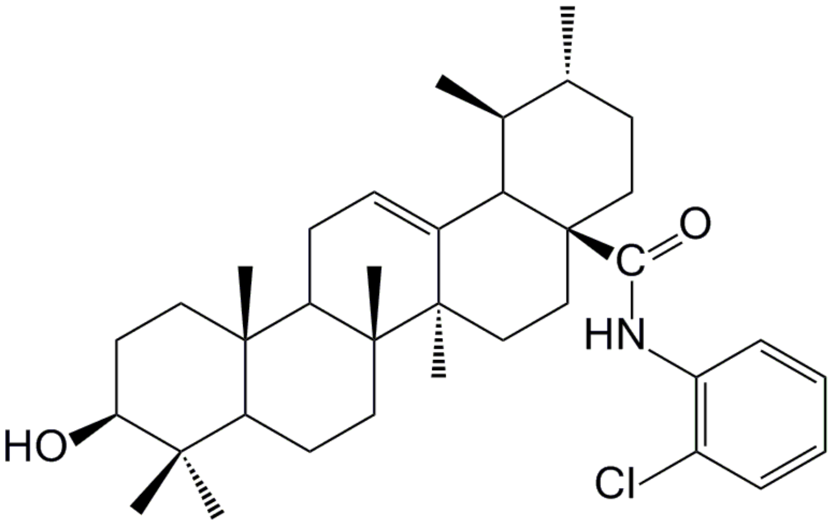

Figure 4. The structure of compound **5b**.

Mass spectrum of compound **5b**:

ESI-MS *m/z* 564.8 $[M-H]^-$

HRMS *m/z* 566.3768 $[M+H]^+$

Calcd for $C_{36}H_{53}ClNO_2$: 566.3759

Peak#:12 Ret.Time:Averaged 32.983-33.017(Scan#:1980-1982)
BG Mode:Calc 32.950<->33.100(1978<->1987)
Mass Peaks:405 Base Peak:564.80(33856) Polarity:Neg Segment1 - Event1

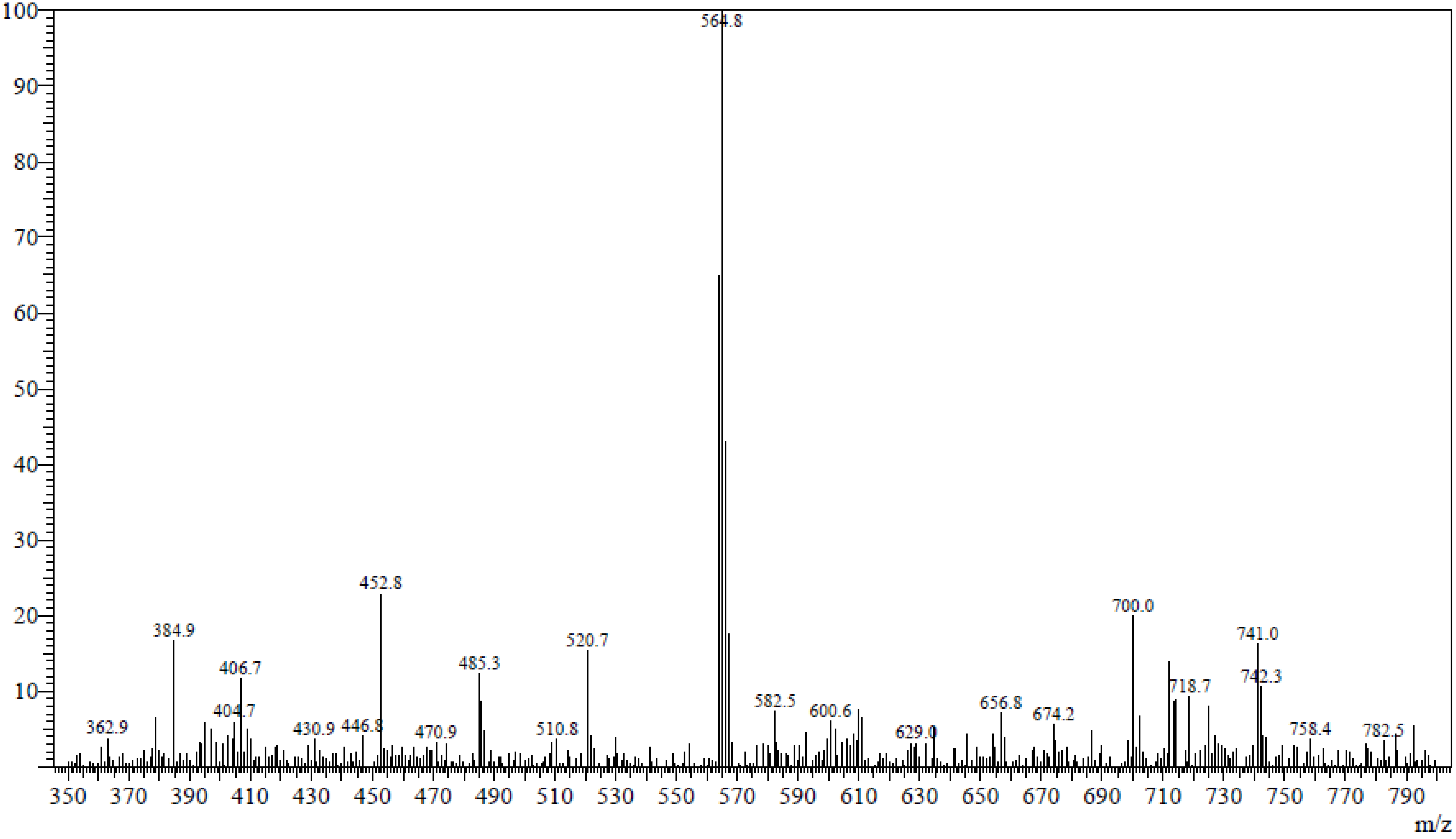

Generic Display Report

Analysis Info

Analysis Name	D:\Data\201404\140414-01\140414-01-8_P1-A-8_01_291.d	Acquisition Date	4/15/2014 3:44:32 PM
Method	esi_pos_50-1000_with calibration_for 1min.m	Operator	BDAL@DE
Sample Name	140414-01-8	Instrument	maXis impact
Comment			

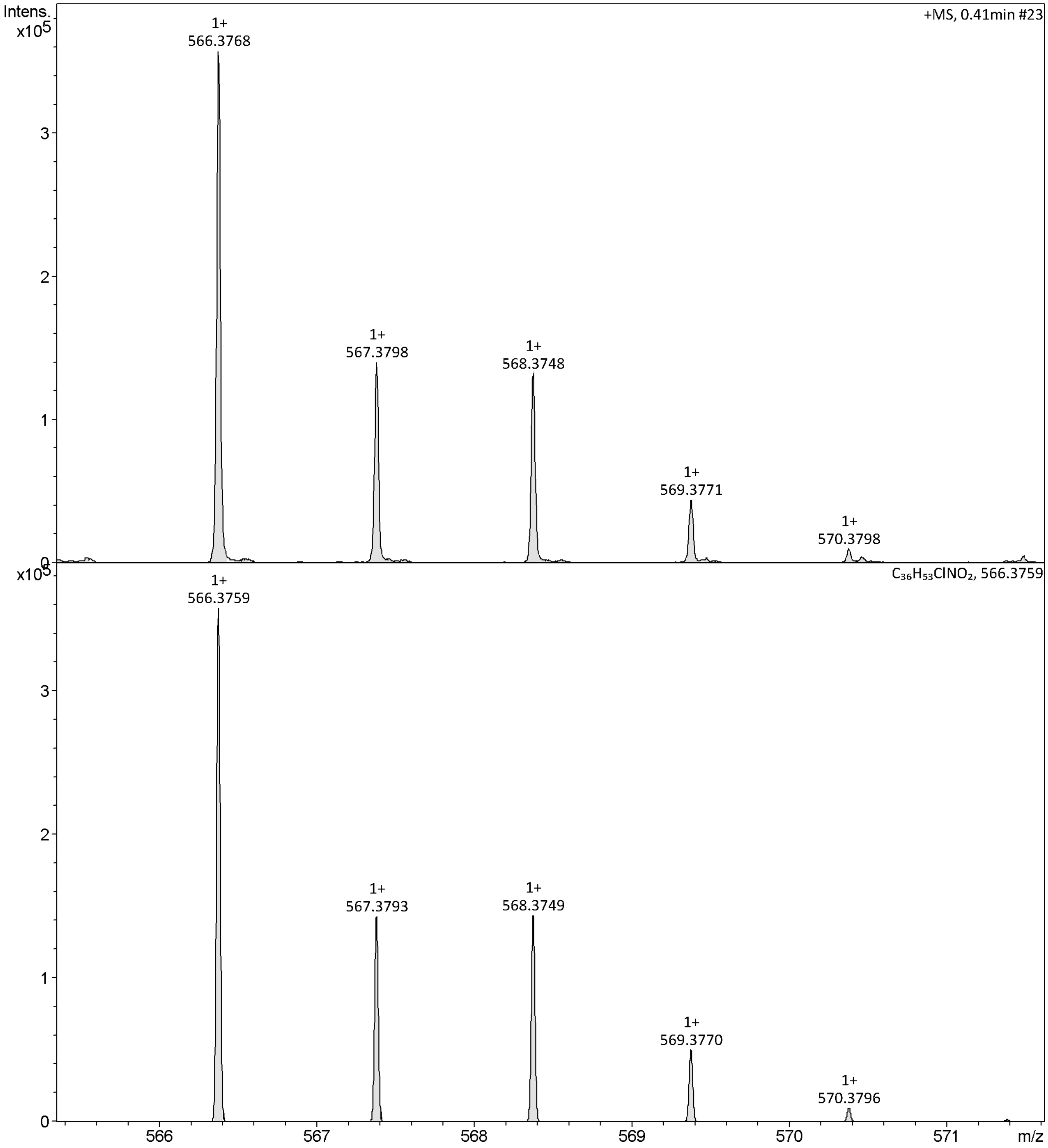

N-[3β-Hydroxy-urs-12-en-28-oyl]-o-bromoaniline

(Compound **6b**)

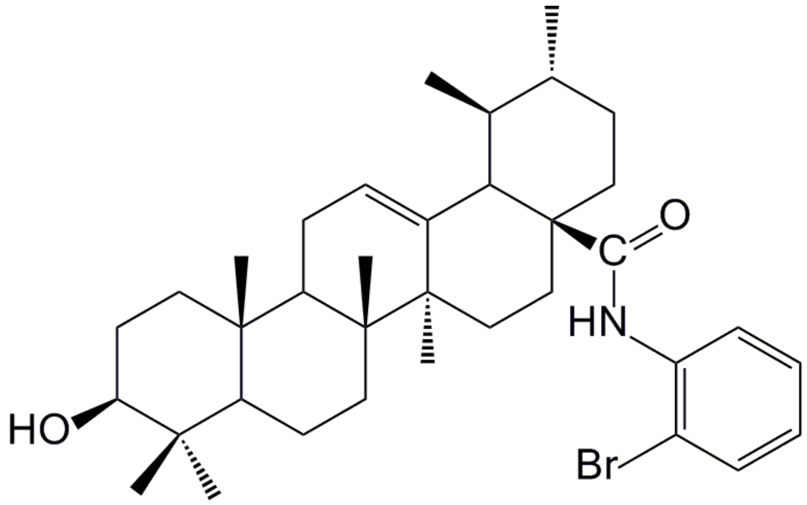

Figure 5. The structure of compound **6b**.

Mass spectrum of compound **6b**:

ESI-MS *m/z* 608.1 $[M-H]^-$

HRMS *m/z* 610.3262 $[M+H]^+$

Calcd for $C_{36}H_{53}BrNO_2$: 610.3254

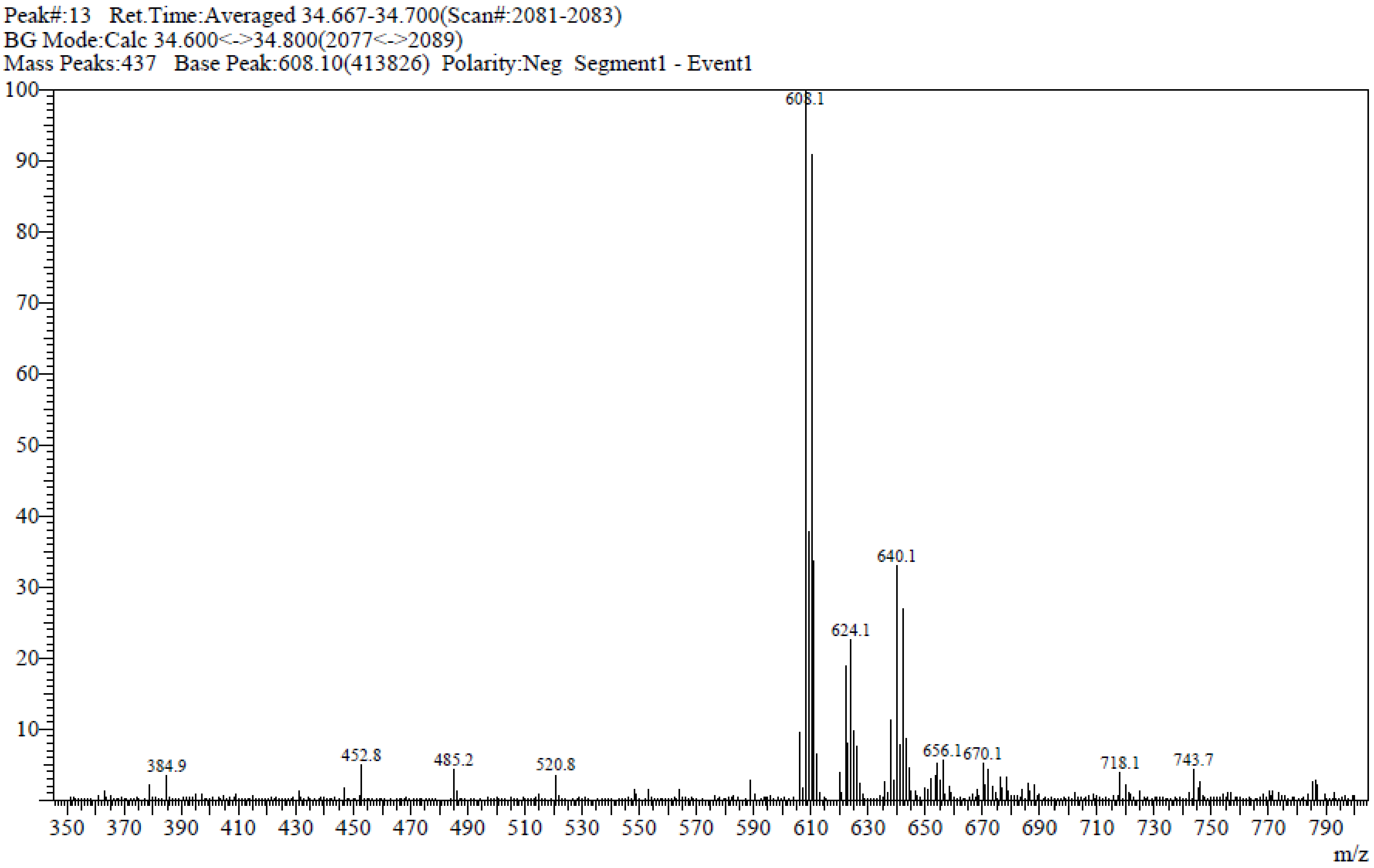
Peak#:13 Ret.Time:Averaged 34.667-34.700(Scan#:2081-2083)
BG Mode:Calc 34.600<->34.800(2077<->2089)
Mass Peaks:437 Base Peak:608.10(413826) Polarity:Neg Segment1 - Event1
608.1
640.1
624.1
384.9
452.8
485.2
520.8
656.1
670.1
718.1
743.7
m/z

Generic Display Report

Analysis Info

Analysis Name	D:\Data\201404\140414-01\140414-01-9_P1-A-9_01_292.d	Acquisition Date	4/15/2014 3:46:03 PM
Method	esi_pos_50-1000_with calibration_for 1min.m	Operator	BDAL@DE
Sample Name	140414-01-9	Instrument	maXis impact
Comment			

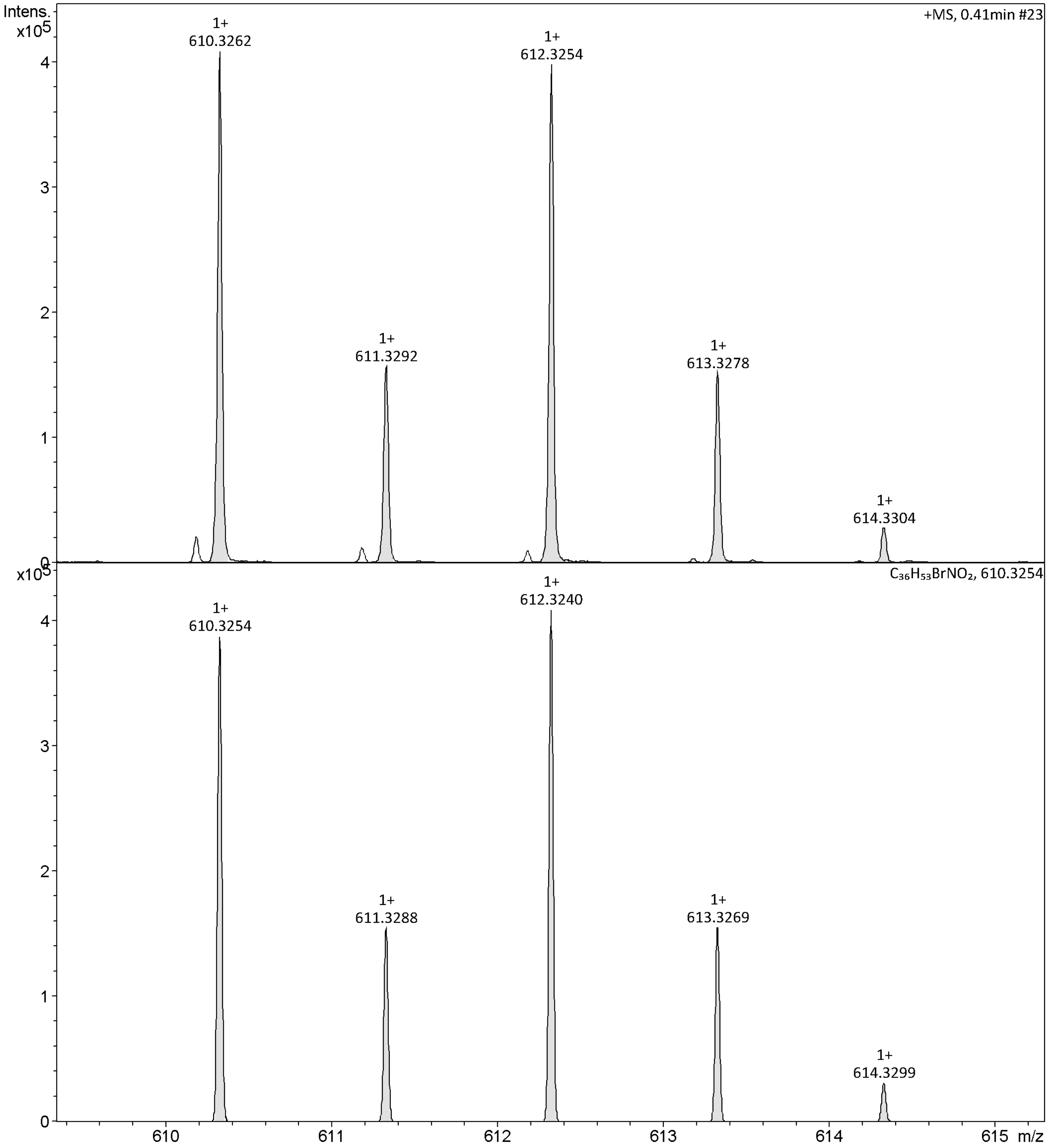

N-[3β-Hydroxy-urs-12-en-28-oyl]-p-fluoroaniline

(Compound **7b**)

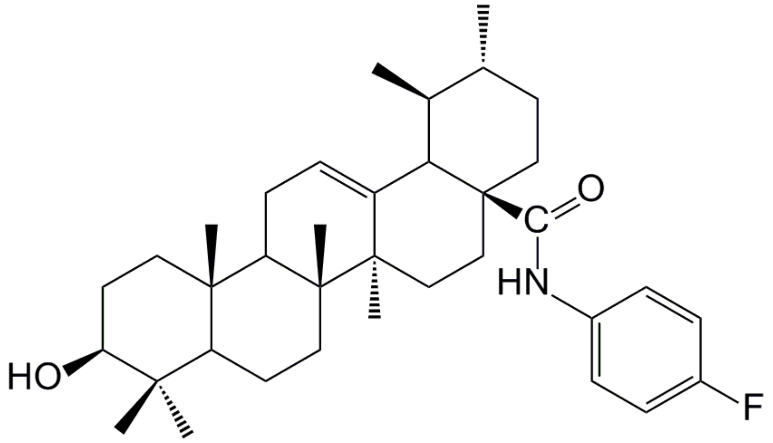

Figure 6. The structure of compound **7b**.

Mass spectrum of compound **7b**:

ESI-MS *m/z* 548.3 $[M-H]^-$

HRMS *m/z* 550.4065 $[M+H]^+$

Calcd for $C_{36}H_{53}FNO_2$: 550.4055

Peak#:2   Ret.Time:Averaged 5.500-5.533(Scan#:331-333)
BG Mode:Calc 5.383<->5.667(324<->341)
Mass Peaks:402   Base Peak:548.25(1267582) Polarity:Neg Segment1 - Event1

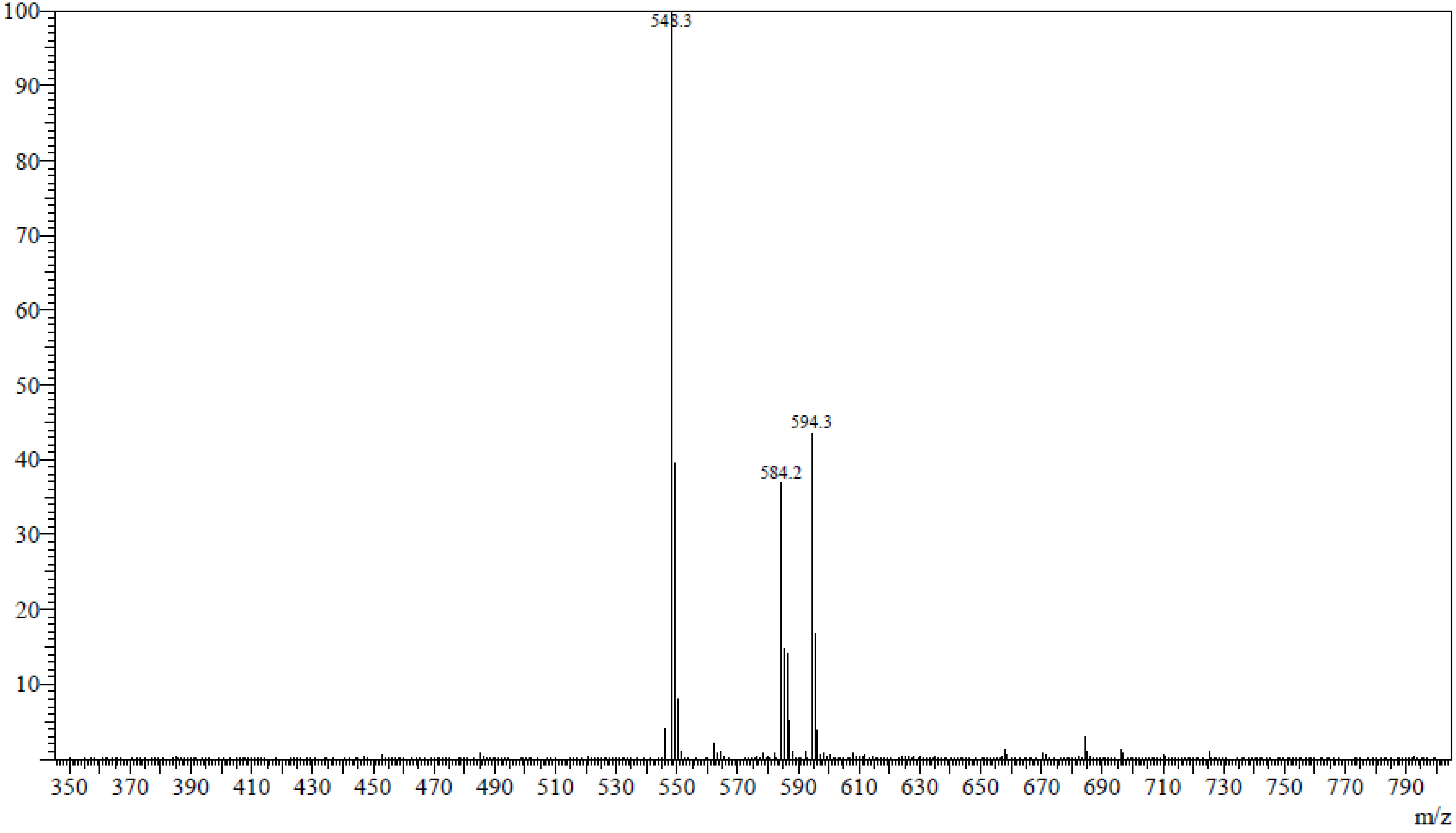

Generic Display Report

Analysis Info

Analysis Name	D:\Data\201404\140414-01\140414-01-4_P1-A-4_01_287.d	Acquisition Date	4/15/2014 3:38:24 PM
Method	esi_pos_50-1000_with calibration_for 1min.m	Operator	BDAL@DE
Sample Name	140414-01-4	Instrument	maXis impact
Comment			

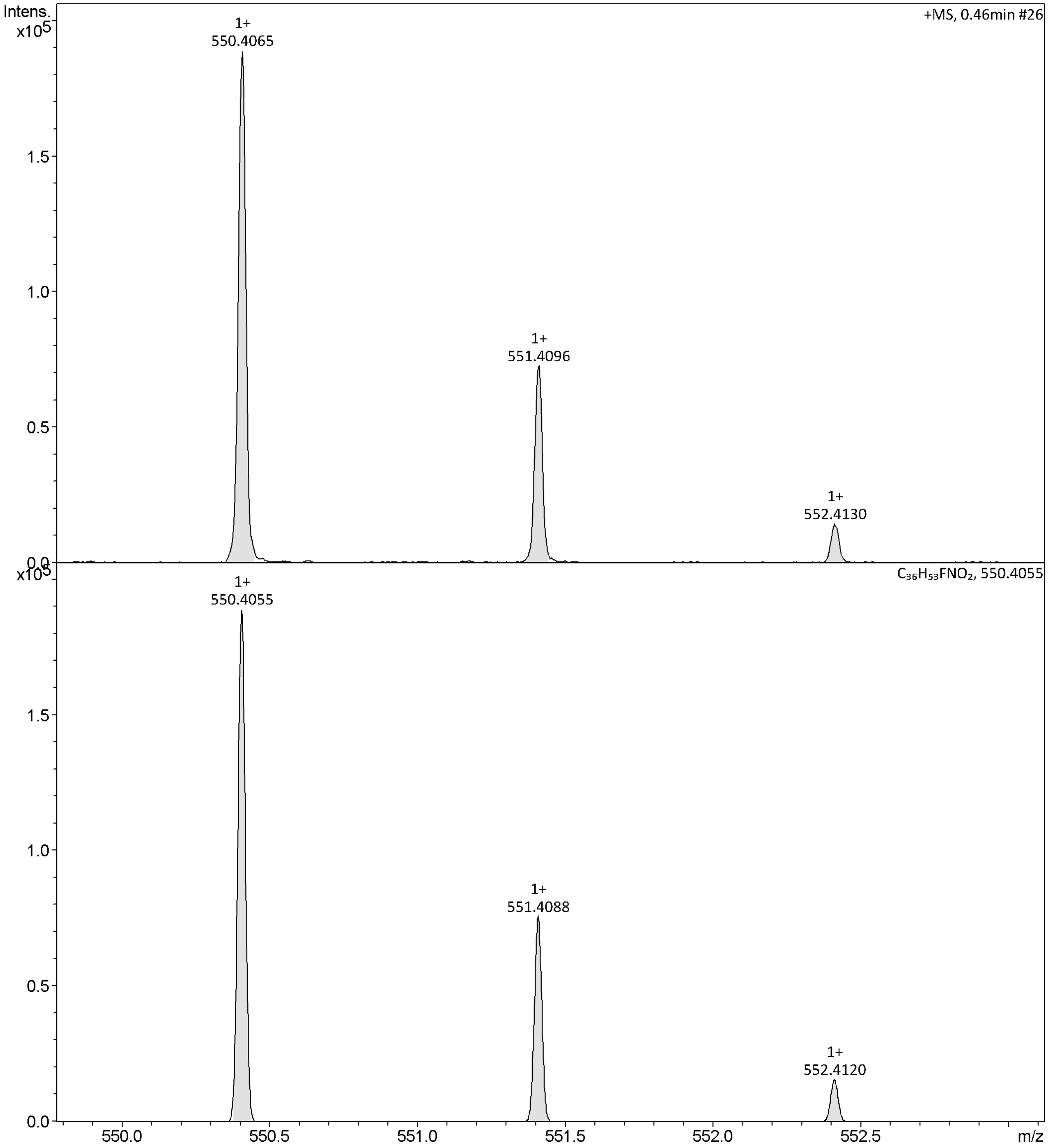

N-[3β-Hydroxy-urs-12-en-28-oyl]-p-chloroaniline

(Compound **8b**)

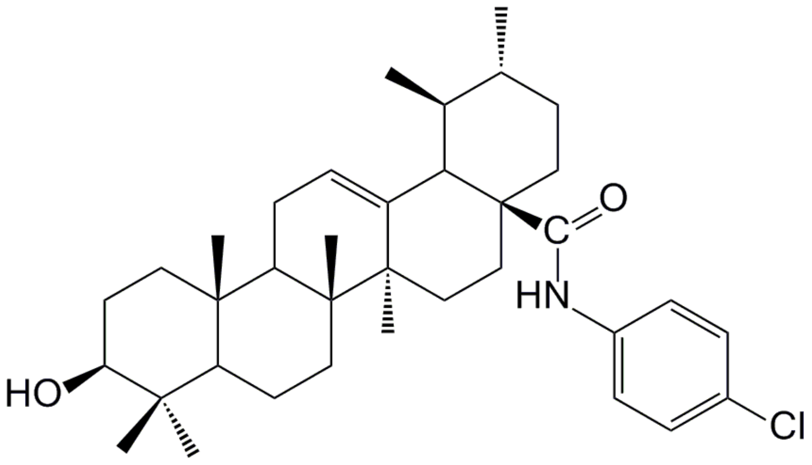

Figure 7. The structure of compound **8b**.

Mass spectrum of compound **8b**:

ESI-MS *m/z* 564.2 $[M-H]^-$

HRMS *m/z* 566.3767 $[M+H]^+$

Calcd for $C_{36}H_{53}ClNO_2$: 566.3759

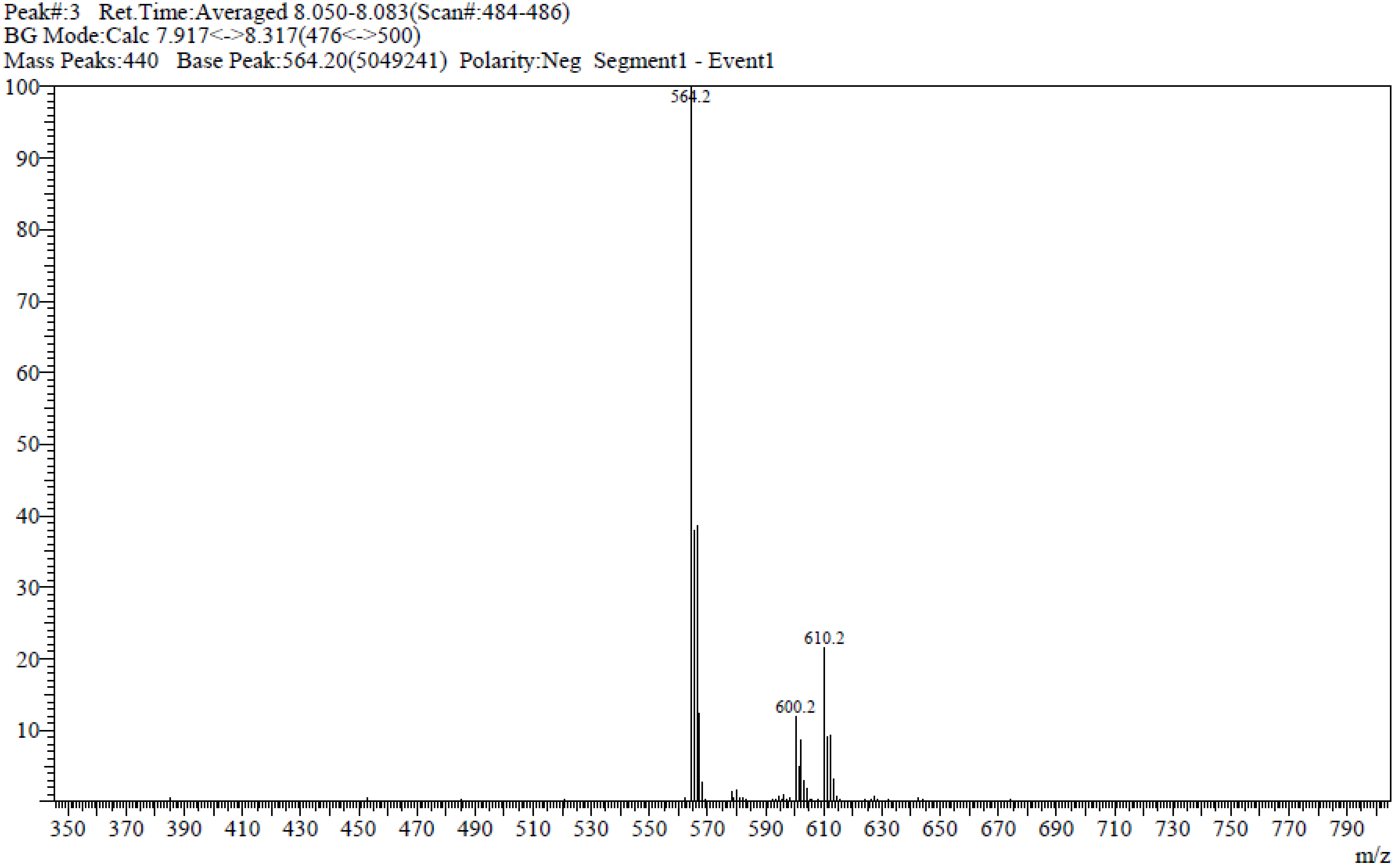
Peak#:3 Ret.Time:Averaged 8.050-8.083(Scan#:484-486)
BG Mode:Calc 7.917<->8.317(476<->500)
Mass Peaks:440 Base Peak:564.20(5049241) Polarity:Neg Segment1 - Event1
564.2
610.2
600.2
m/z

Generic Display Report

Analysis Info

Analysis Name	D:\Data\201404\140414-01\140414-01-5_P1-A-5_01_288.d	Acquisition Date	4/15/2014 3:39:56 PM
Method	esi_pos_50-1000_with calibration_for 1min.m	Operator	BDAL@DE
Sample Name	140414-01-5	Instrument	maXis impact
Comment			

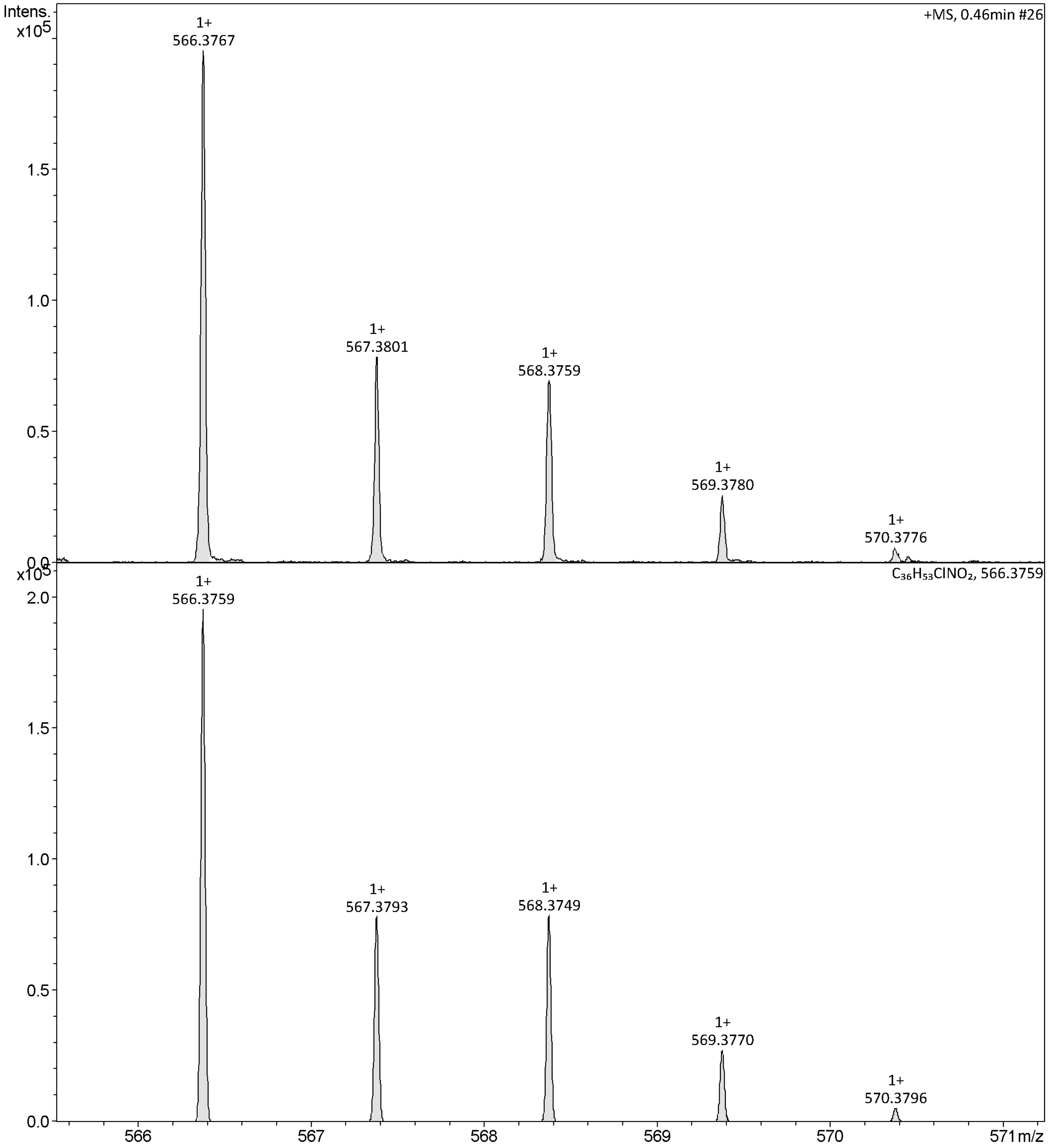

N-[3β-Hydroxy-urs-12-en-28-oyl]-p-bromoaniline

(Compound **9b**)

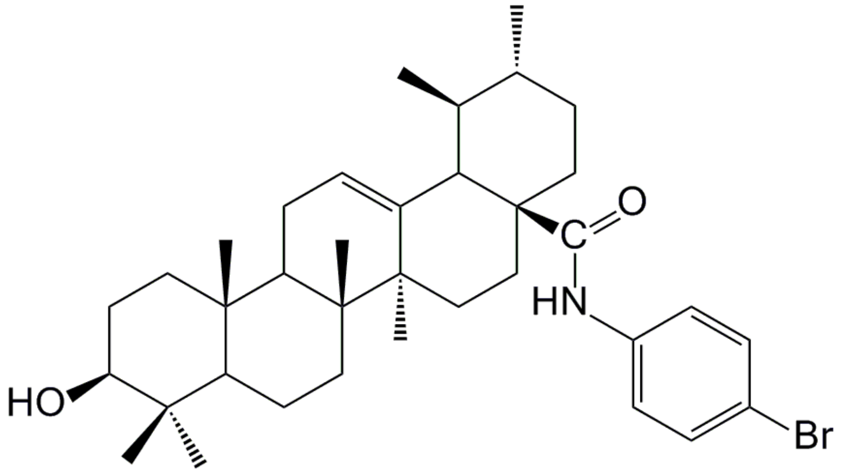

Figure 8. The structure of compound **9b**.

Mass spectrum of compound **9b**:

ESI-MS *m/z* 610.1 $[M-H]^-$

HRMS *m/z* 610.3260 $[M+H]^+$

Calcd for $C_{36}H_{53}BrNO_2$: 610.3254

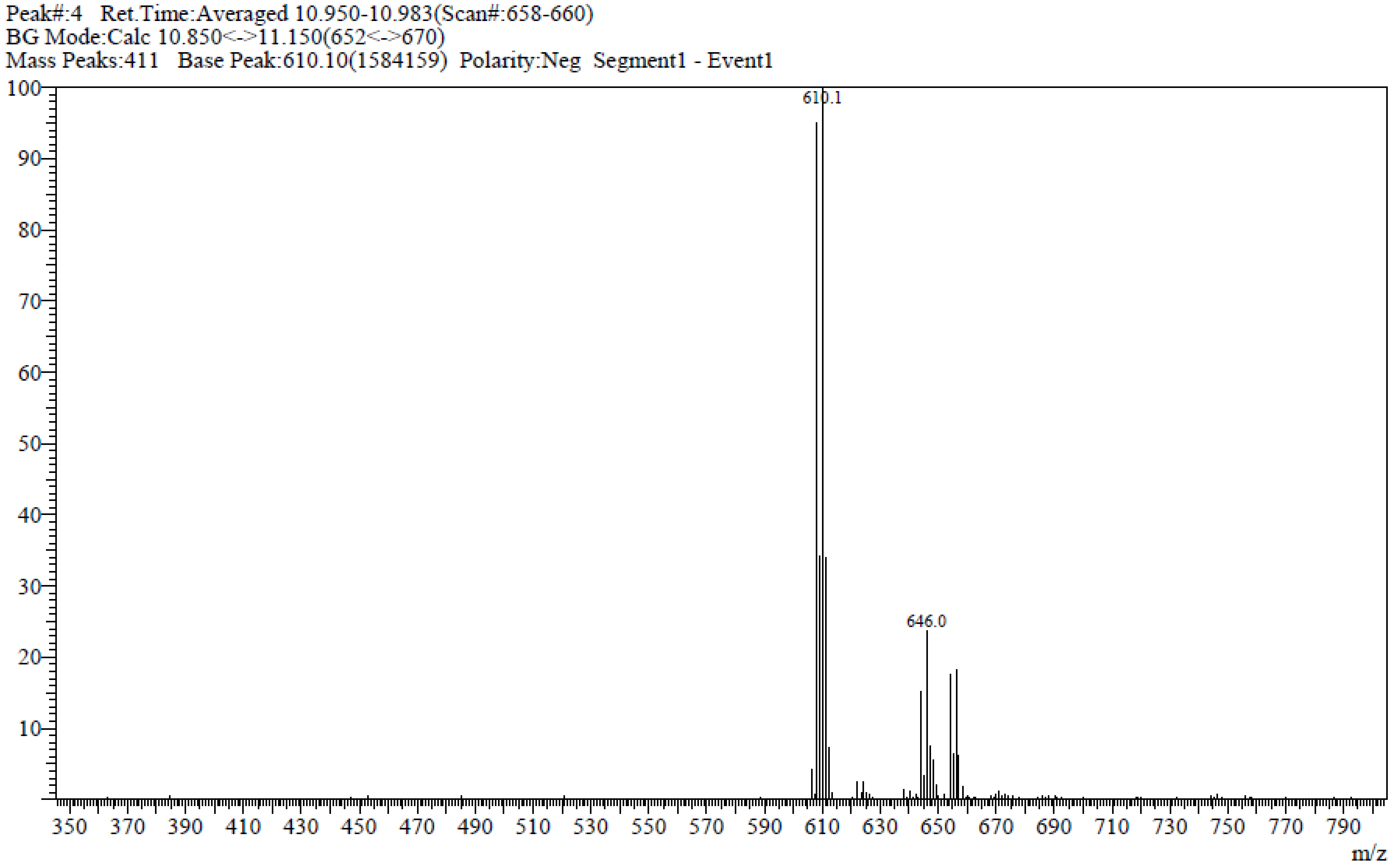

Peak#:4 Ret.Time:Averaged 10.950-10.983(Scan#:658-660)
BG Mode:Calc 10.850<->11.150(652<->670)
Mass Peaks:411 Base Peak:610.10(1584159) Polarity:Neg Segment1 - Event1
610.1
646.0
100
90
80
70
60
50
40
30
20
10
350 370 390 410 430 450 470 490 510 530 550 570 590 610 630 650 670 690 710 730 750 770 790
m/z

Generic Display Report

Analysis Info

Analysis Name	D:\Data\201404\140414-01\140414-01-6_P1-A-6_01_289.d	Acquisition Date	4/15/2014 3:41:29 PM
Method	esi_pos_50-1000_with calibration_for 1min.m	Operator	BDAL@DE
Sample Name	140414-01-6	Instrument	maXis impact
Comment			

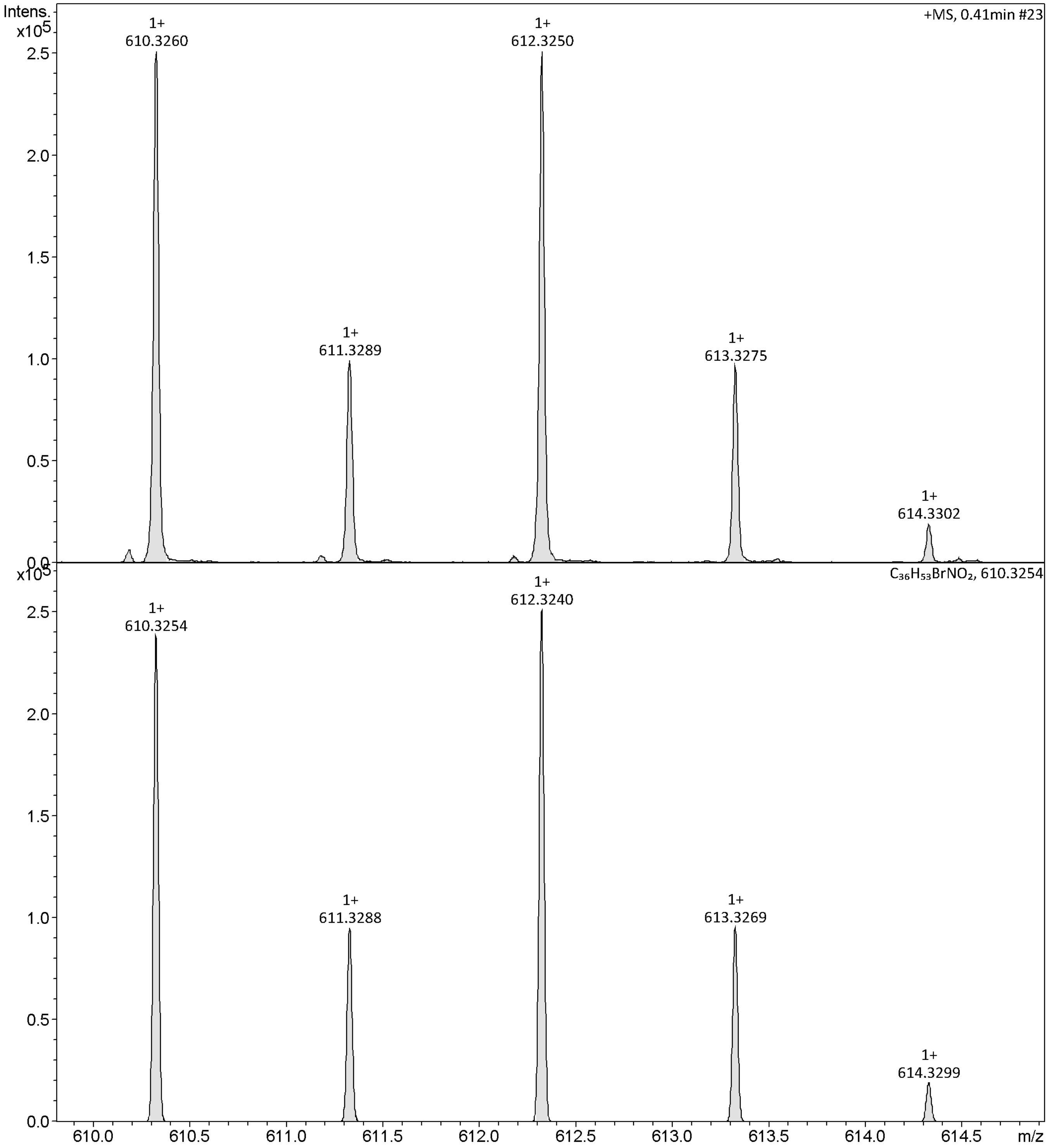

N-[3β-Hydroxy-urs-12-en-28-oyl]-p-methoxylaniline

(Compound **10b**)

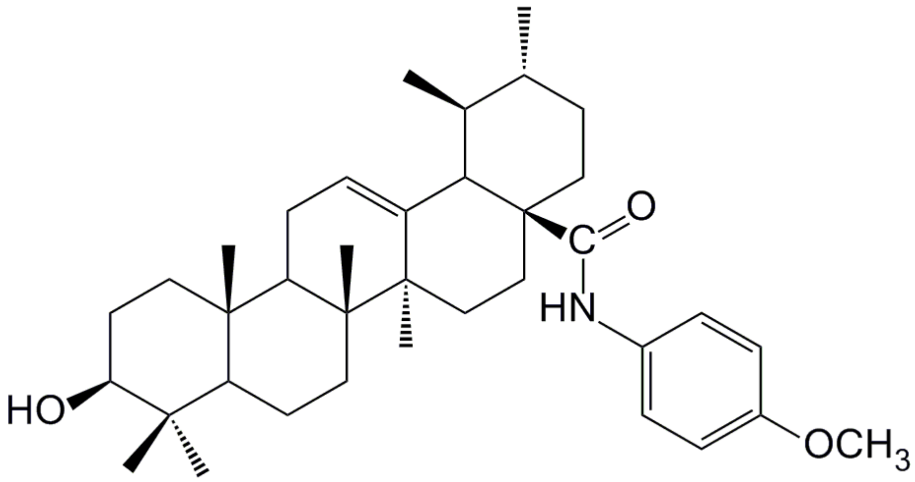

Figure 9. The structure of compound **10b**.

Mass spectrum of compound **10b**:

ESI-MS *m/z* 560.3 $[M-H]^-$

HRMS *m/z* 562.4274 $[M+H]^+$

Calcd for $C_{37}H_{56}NO_3$: 562.4255

Peak#:11 Ret.Time:Averaged 31.467-31.500(Scan#:1889-1891)
BG Mode:Calc 31.383<->31.633(1884<->1899)
Mass Peaks:421 Base Peak:560.25(457573) Polarity:Neg Segment1 - Event1

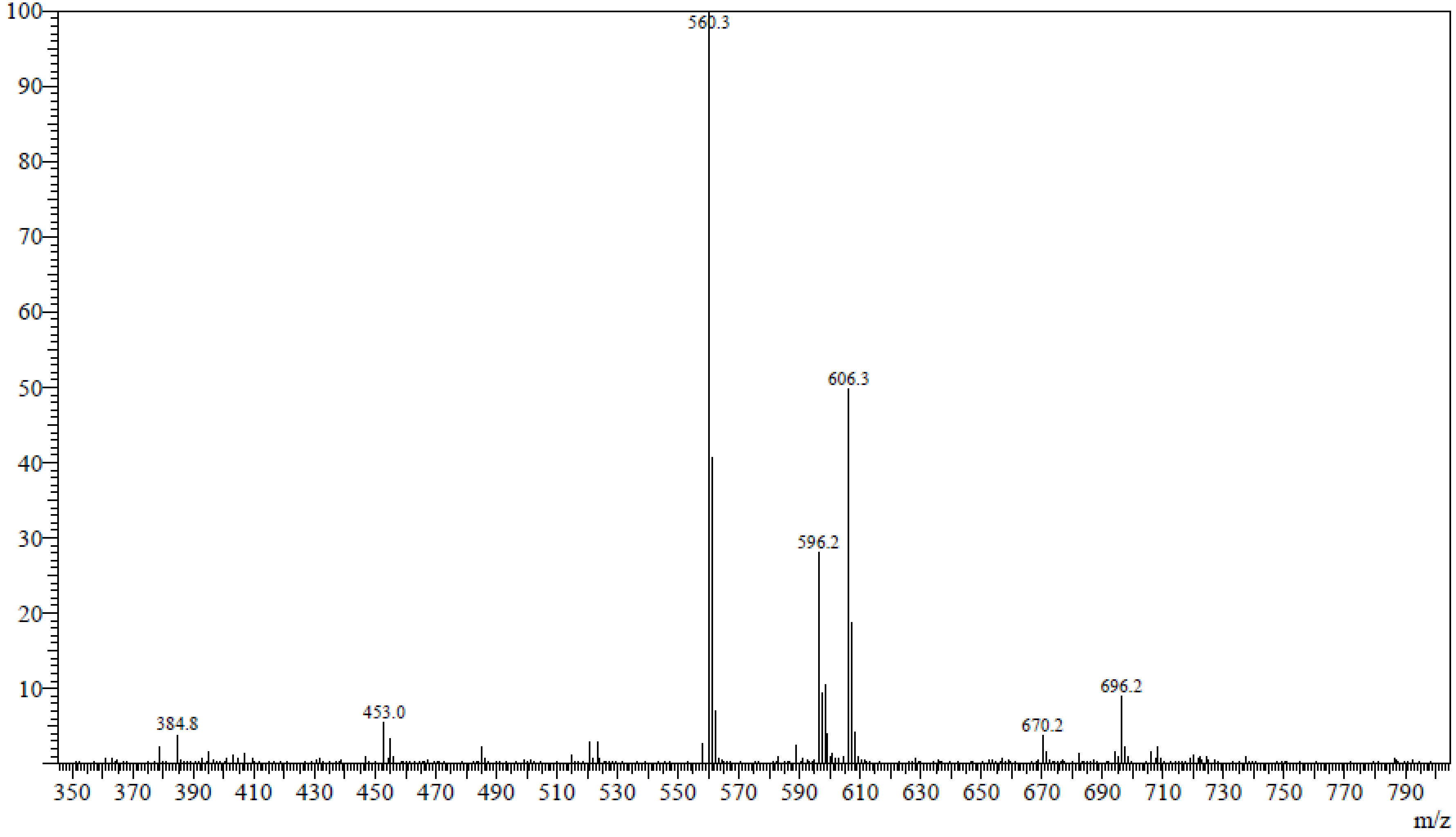

Generic Display Report

Analysis Info

Analysis Name	D:\Data\201404\140414-01\140414-01-2_P1-A-2_01_285.d	Acquisition Date	4/15/2014 3:35:22 PM
Method	esi_pos_50-1000_with calibration_for 1min.m	Operator	BDAL@DE
Sample Name	140414-01-2	Instrument	maXis impact
Comment			

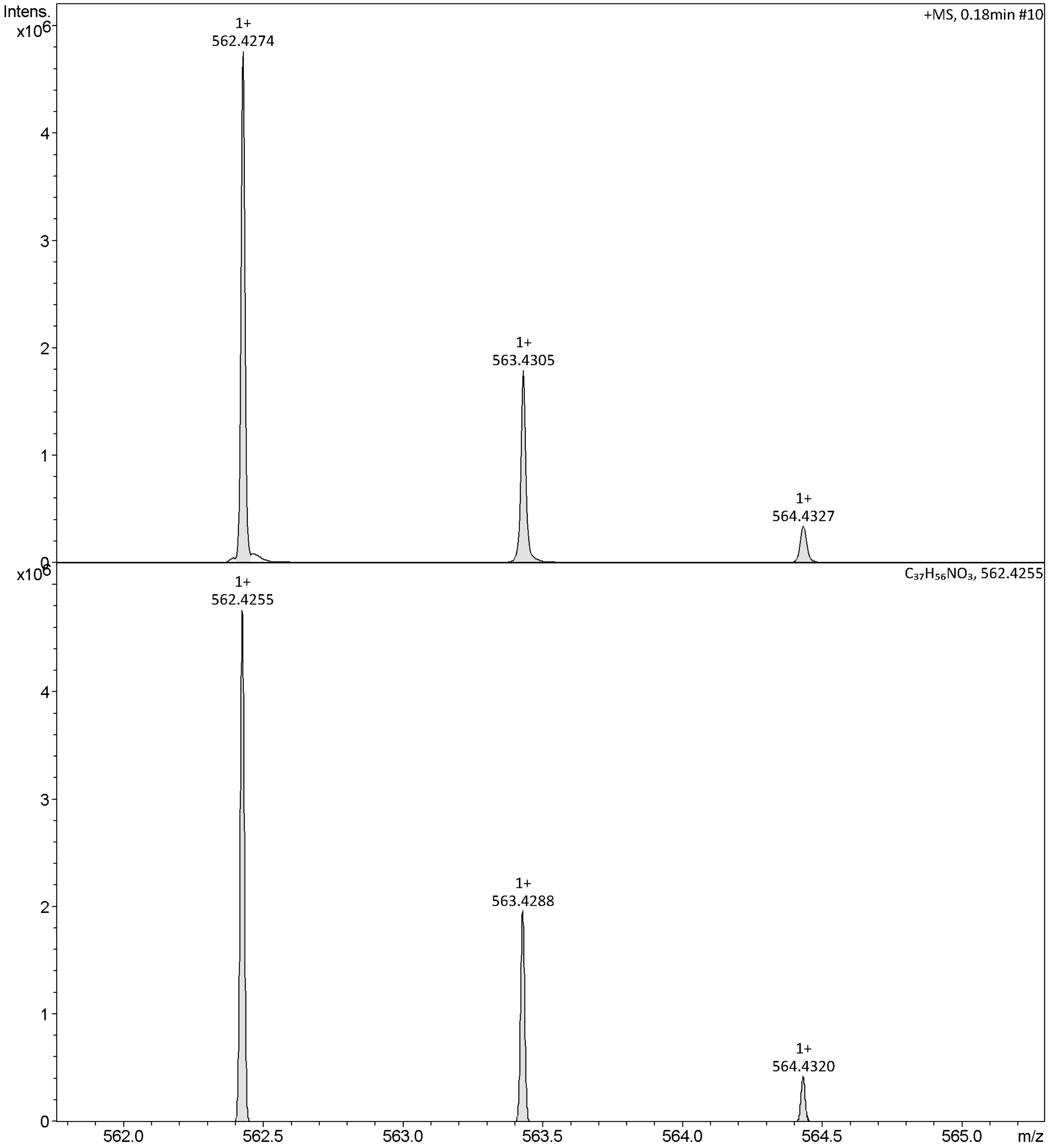

3-Oxo-urs-12-en-28-oic acid

(Compound **11**)

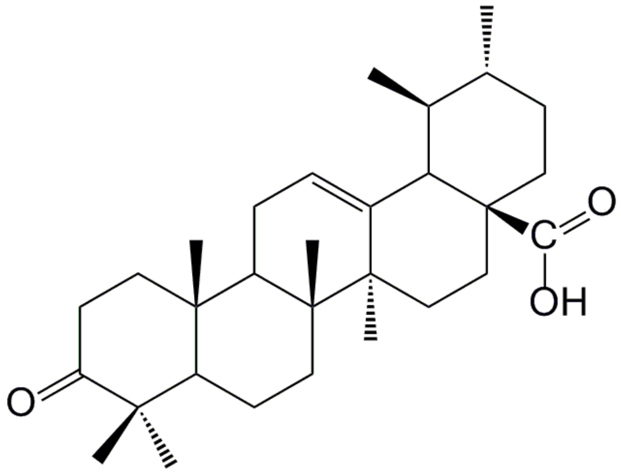

Figure 10. The structure of compound **11**.

Mass spectrum of compound **11**:

ESI-MS *m/z* 453.2 $[M-H]^-$

Peak#:8 Ret.Time:Averaged 25.517-25.550(Scan#:1532-1534)
BG Mode:Calc 25.417<->25.717(1526<->1544)
Mass Peaks:248 Base Peak:453.20(472736) Polarity:Neg Segment1 - Event1

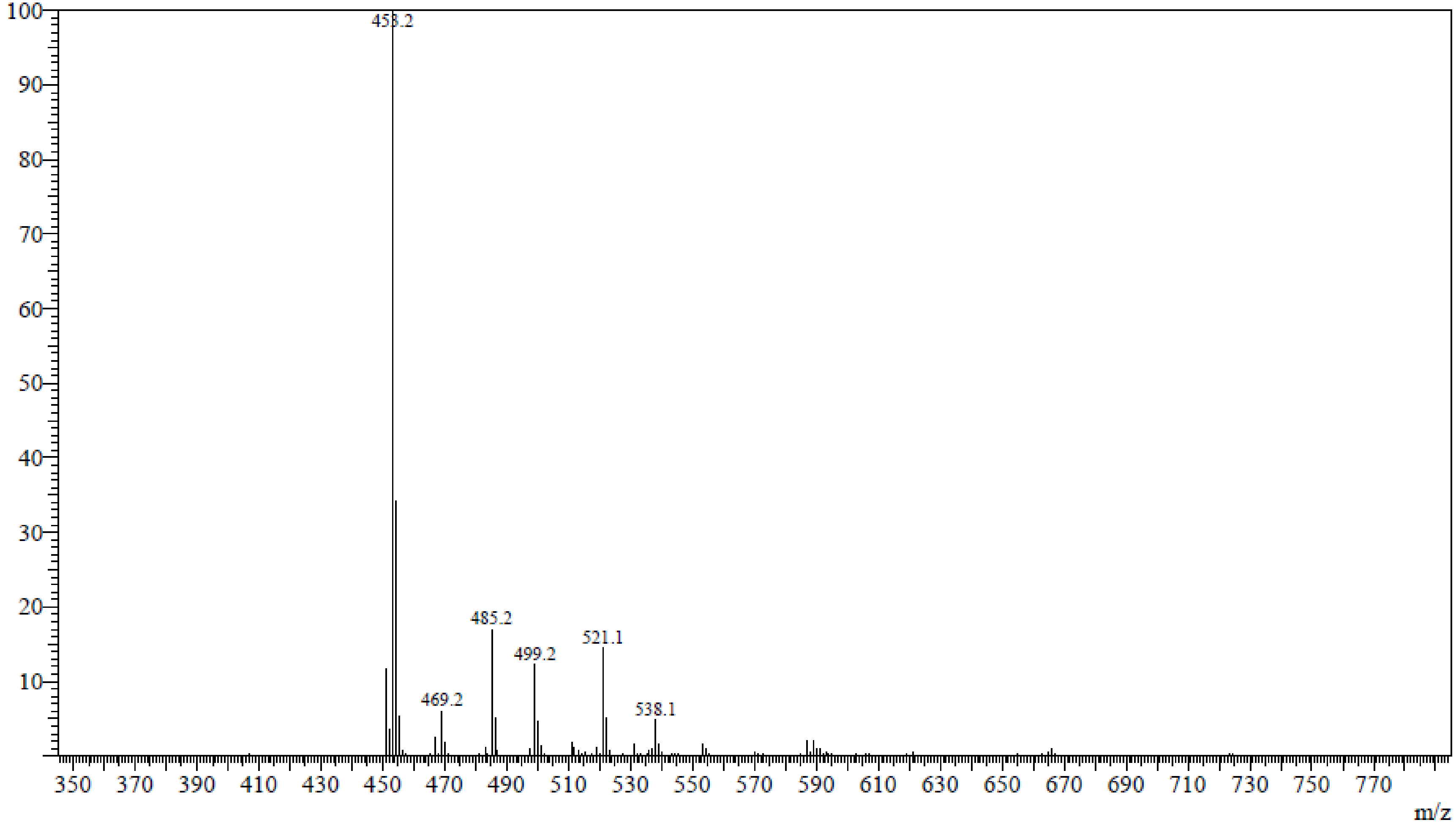

Supplement: S2 File — (PDF) [file pone.0138767.s002.pdf]
