# Supplementary material for: Synthesis and Evaluation of Novel Triterpene Analogues of Ursolic Acid as Potential Antidiabetic Agent
Source: PLoS One. 2015 Sep 25;10(9):e0138767. doi: 10.1371/journal.pone.0138767 (PMC4583267; doi:10.1371/journal.pone.0138767)
Supplement: S3 File — (DOCX) [file pone.0138767.s003.docx]

**S3 Fig. The structure of UA and its analogues which were studied in QSAR model.**

The structure of these analogues and their bioactivities could be obtained in this study or our previous work [18, 20].

18 Wu PP, Zhang K, Lu YJ, He P, Zhao SQ. In vitro and in vivo evaluation of the antidiabetic activity of ursolic acid derivatives. Eur J Med Chem. 2014; 80: 502-508.

20 Huang TM, Wu PP, Cheng AM, Qin Jing, Zhang K, Zhao SQ. A hydrophilic conjugate approach toward the design and synthesis of ursolic acid derivatives as potential antidiabetic agent. RSC Adv. 2015; 5(55): 44234-44246.
